# Outcomes of resin infiltration for white spot lesions at different time points: a systematic review and meta-analysis

**DOI:** 10.1093/ejo/cjag021

**Published:** 2026-05-15

**Authors:** Laura Ståhl, Spyridon N Papageorgiou, Anna Westerlund, Julia Naoumova

**Affiliations:** Department of Orthodontics, Institute of Odontology, Sahlgrenska Academy, University of Gothenburg, Box 450, 405 30 Gothenburg, Sweden; Clinic of Orthodontics, University Dental Care, Public Dental Service, Region Västra Götaland, Box 7163, 402 30 Gothenburg, Sweden; Clinic of Orthodontics and Pediatric Dentistry, Center for Dental Medicine, Plattenstrasse 11, 8032 Zürich, Switzerland; Department of Orthodontics, Institute of Odontology, Sahlgrenska Academy, University of Gothenburg, Box 450, 405 30 Gothenburg, Sweden; Department of Orthodontics, Institute of Odontology, Sahlgrenska Academy, University of Gothenburg, Box 450, 405 30 Gothenburg, Sweden; Clinic of Orthodontics, University Dental Care, Public Dental Service, Region Västra Götaland, Box 7163, 402 30 Gothenburg, Sweden

**Keywords:** dental caries, enamel demineralization, minimally invasive dentistry, esthetic dentistry, orthodontics, demineralized enamel, color stability, PROM

## Abstract

**Background:**

White spot lesions (WSLs) are a common clinical finding with implications for both individuals and society. Several treatment approaches have been proposed, among which resin infiltration (RI) has emerged as a promising option.

**Objective:**

Investigate clinical and patient-reported outcomes of RI on WSLs at different time points.

**Search methods:**

Unrestricted literature searches were conducted in six databases for human studies until September 2025.

**Selection criteria:**

Randomized and non-randomized clinical studies on patients with WSLs on at least one anterior tooth treated with RI assessing different clinical- and patient-reported outcomes with a minimum patient follow-up of 3 months.

**Data collection and analysis:**

Study selection, data extraction, and risk of bias assessment were performed in duplicate. Random-effects meta-analyses of pooled average changes between post-treatment and follow-up time points with their corresponding 95% confidence interval (CI) were performed, followed by sensitivity analyses.

**Results:**

Thirty-four reports pertaining to 29 studies were included covering 544 patients (ages 7–40 years; 44% male on average) and 1495 teeth with WSLs. The risk of bias assessment revealed concerns mainly due to the small sample sizes, short follow-up periods, absence of blinded measurements, and lack of analysis of confounding factors or within-patient clustering. For most outcomes, RI showed inconsistent and heterogeneous effects. In the largest meta-analysis (six studies) comparing the standardized color difference (Δ*E*) between WSL and sound adjacent enamel from post-treatment to 6 months, the pooled average change was 0 (95% CI −0.42 to 0.41; *P* = .98), with high heterogeneity (*I*^2^ = 84%). A small meta-analysis showed significant improvements in patient satisfaction through a Visual Analogue Scale from post-treatment to 1 month (two studies; +11.9 mm; 95% CI +5.9 to +17.8 mm; *P* = .02), which diminished to non-significant levels at the 3 (*P* = .09), 6 (*P* = .20) and 12 months (*P* = .94). Twelve months post-treatment, no significant differences were seen for Δ*Ε*, fluorescence, patient satisfaction, or WSL area (*P* > .05).

**Limitations:**

Among the included studies, high between-study heterogeneity was observed for most outcome measures, while most studies featured small sample sizes and short follow-up times.

**Conclusions:**

RI for WSLs shows highly inconsistent effects, compounded by limited follow-ups, highlighting the need for larger and standardized studies with extended follow-up to better evaluate treatment outcomes of resin-infiltrated WSLs.

**Registration:**

PROSPERO CRD42024611702.

## Introduction

### Background

Among oral diseases, dental caries of permanent teeth is the most common, affecting around two billion people globally [1]. White spot lesions (WSLs) or early caries lesions are an initial clinical sign of enamel demineralization, which are characterized as white, chalky opacities along the gingival margin or around bonded orthodontic brackets [2]. The estimated prevalence of WSL after orthodontic treatment ranges from 27.7% to 97.0% [3], even though their development is complex and multifactorial. Inadequate control of the dental biofilm combined with frequent intake of fermentable carbohydrates creates a dysbiotic biofilm, in which acid production by cariogenic bacteria is the immediate cause of subsurface enamel demineralization [4–6]. Additional risk factors include systemic diseases and medications, particularly those affecting salivary flow [7]. More recently, specific host genetic variants have also been implicated in caries development [8, 9]. Together with strain-dependent differences in the cariogenic potential of Streptococcus mutans [10], these genetic factors appear to shape the balance between microbial acid attack and protective host defense mechanisms in the dental biofilm [11].

The presence of orthodontic appliances exacerbate the risk by hindering effective oral hygiene, providing additional surfaces for bacterial adhesion [12], and reducing salivary flow and pH [13, 14]. Consequently, orthodontic patients often exhibit increased levels of cariogenic bacteria and a higher risk for caries development [15].

According to laypersons’ perceptions, WSLs are noticeable and negatively affect dental esthetics [16–18], overall attractiveness, and social as well as professional parameters [16, 19–21]. For affected individuals, enamel opacities can cause both symptomatic and esthetic consequences [22, 23], often accompanied by reduced self-esteem [24]. Even though preventive measures against WSLs can be taken [25], WSLs may still nevertheless progress to manifest caries requiring restorative treatment with substantial financial costs for patients and/or society [26, 27], and in severe cases, may lead to tooth loss or adversely affect general health [28].

Given the societal, economic, professional, and individual burden of dental caries, early intervention is essential. Numerous treatment modalities, presented in several systematic reviews, have been proposed to treat WSLs, including topical fluoride, casein phosphor peptides-amorphous calcium phosphate (CPP-ACP), self-assembled peptide (SAP) P11-4, nano-hydroxyapatite, and micro-abrasion [29–33]. Another treatment showing promising results in masking WSLs is the micro-invasive intervention resin infiltration (RI) [34–36]. RI consists of a light-flowing plastic (resin) that penetrates the demineralized areas. The procedure begins with pre-treatment of the tooth by etching with hydrochloric acid (15%), followed by drying with alcohol (99%), which allows the otherwise intact enamel prisms to be sufficiently opened to allow light-flowing resin (methacrylate-based resin matrix) to penetrate the enamel by capillary forces. Demineralized enamel has a refractive index of 1.00–1.33, whereas sound enamel has a refractive index of 1.63 [37–39]. RI, with a refractive index of 1.53, approximates that of sound enamel, rendering the WSL less visible. Additional benefits include an increased microhardness and further demineralization resistance [40].

Previous systematic reviews indicated that RI is effective in treating WSLs [30, 34, 41–43]. However, concerns remain regarding the long-term outcome of RI due to heterogeneous protocols among existing studies and small sample sizes. It has been recommended that future research focuses on long-term outcomes, more homogeneous methodology, and particular attention to color stability or discoloration risk [43, 44]. Furthermore, there is still a lack of high-quality systematic reviews to firmly establish the effectiveness of RI [45]. As this field is rapidly evolving and the latest systematic reviews, with searches covering up to mid-2024 [46, 47], did not perform quantitative meta-analyses, the present review aims to address this gap by conducting an extensive systematic search and providing a comprehensive quantitative synthesis across multiple outcomes and follow-up time points.

### Objective

The aim of this systematic review was to evaluate the effects of RI for WSLs at different time points, regarding both clinical and patient-reported outcomes.

## Materials and methods

### Protocol and registration

The review protocol was developed *a priori* and registered in PROSPERO (CRD42024611702). All changes to the initial protocol are reported (Supplementary Material S1). This systematic review was conducted and reported according to the Cochrane Handbook [48] and the Preferred Reporting Items for Systematic reviews and Meta-Analyses (PRISMA) 2020 statement guidelines [49], respectively.

### Research question and eligibility criteria

The Participants-Intervention-Comparison-Outcome-Study design framework for this review was based on the research question “What are the effects of resin infiltration on white spot lesions at different time points?”: P (Population): Individuals of any age, sex or ethnicity with at least one WSL buccally on anterior teeth or canines; I (Intervention): RI; C (Comparison): Comparisons across different time points, within resin infiltrated enamel, or RI compared with sound adjacent enamel (SAE); O (Outcome): Follow-up (of at least 3 months) results on color changes (primary outcome), or change in fluorescence, lesion size, caries progression, or patient-reported outcome measures (PROMs); S (Study design): Randomized clinical trials, non-randomized (single-group or comparative) cohort studies, case–control studies, and cross-sectional studies involving human participants. The full eligibility criteria are presented in Table 1.

**Table 1:** The eligibility criteria for the systematic review.

	Inclusion	Exclusion
P	At least one WSL buccally on anterior tooth or canine Any age, sex, or ethnicity who are systemically healthy No restriction regarding study population or sample size	WSL only on premolar or molar or approximal Only other diagnosis like fluorosis, MIH, amelogenesis imperfecta, or due to traumatic injury
I	Treated with RI	Other treatments or combination of treatments of WSLs Preventive treatment
C	Within RI, RI compared with sound enamel Different time points, baseline, direct after treatment (<10 days) and ≥3 months follow-up	Only results directly after treatment, follow-up less than 3 months, or no follow-up
O	Any type of outcome; change in color or fluorescence, lesion size, caries progression, patient-reported outcome measures	
S	Randomized clinical trials, non-randomized (single-group or comparative) cohort studies, case–control studies and cross-sectional studies involving human participants No restriction regarding language or publication status	Review articles, letters, case reports, opinion pieces, in-vitro and animal studies

MIH, molar incisor hypomineralization; RI, resin infiltration; WSL, white spot lesion.

### Information sources and search

Six databases were systematically searched (MEDLINE via PubMed, Embase, Scopus, Web of Science, Cochrane Library, LILACS) without restrictions on publication language, year, or status. The primary search was made 5 December 2024 by librarian Helen Sjöblom, Biomedical Library, Gothenburg University Library, Gothenburg, Sweden. An additional search (29 September 2025) was performed prior to manuscript submission to capture any newly published articles. Detailed search strategies for each database are provided in Supplementary Material S2. Furthermore, the reference lists and citations of eligible articles, as well as existing systematic reviews, were manually screened using Google Scholar to identify additional relevant studies.

### Selection process

The screening was performed using the Rayyan software program [50]. Two reviewers (L.S. and J.N.) independently screened the titles and/or abstracts of all studies retrieved from the searches to identify potential eligible articles. Thereafter, full texts of the potentially relevant articles were retrieved and independently screened by the same two authors. Reasons for exclusion of studies in this phase were carefully documented. Reviewers were blinded to each other’s decisions until both submitted their assessments. Any disagreements were resolved by discussion with a third reviewer (A.W.). The complete list of excluded articles with reasons for exclusion, as well as the included articles, is provided in Supplementary Material S4.

### Data collection process and items

The following data was extracted from the included articles: (i) study characteristics, including first author, year of publication, country where trial was conducted, and study design; (ii) sample size, in terms of both patients and teeth; (iii) participant demographics, i.e. gender, and age; (iv) localization of WSLs, i.e. jaw and specific teeth; (v) details of the intervention and control; (vi) follow-up time; (vii) methods used for data collection at baseline and follow-up; and (viii) outcomes assessed. Data extraction was performed independently by two reviewers (L.S. and A.W.), with any discrepancies resolved through discussion with a third reviewer (J.N.). When important data was missing or unclear, attempts were made to contact the corresponding author for clarification (Supplementary Material S5).

### Risk of bias of individual studies

All assessments were performed independently by two reviewers (L.S. and A.W.), with any discrepancies resolved through discussion with a third reviewer (J.N.). Even though randomized and non-randomized comparative clinical studies were included, these were included in this review to assess between-time points differences in the RI group only. As such, a single-group prospective cohort study was the ideal study design for this review and all included studies were evaluated for internal validity, completeness of reporting, and factors potentially affecting risk of bias using a customized tool designed for cohort studies, adapted from the Joanna Briggs Institute checklist for cohort studies [51]. The complete risk of bias assessment tool is provided in Supplementary Material S3.

### Effect measures and data synthesis

All available data was sought, but in randomized controlled trials only RI groups were analyzed, as our review focused exclusively on outcomes at different time points within resin-treated teeth, and comparative effectiveness against other interventions fell outside the scope. To maximize data yield, we attempted to include all studies, independent of reporting completeness, and data was calculated by ourselves, when necessary (Supplementary Material S1). A single-group meta-analysis for pooled average changes between post-treatment and follow-up time points with their corresponding 95% confidence interval (CI), was undertaken. As the effects of RI were expected to vary across patients (due to lesion characteristics, clinical protocol, oral hygiene levels, and oral habits), a random-effects model was chosen *a priori* to calculate their average distribution among the included studies. Between-study heterogeneity was assessed by inspecting forest plots and calculating tau-squared (absolute heterogeneity) and *I*^2^ (relative inconsistency) statistics, respectively. *I*^2^ defines the proportion of total variability in the result explained by heterogeneity and not chance. We considered the extent, direction of heterogeneity (localization on the forest plot), and uncertainty intervals around heterogeneity estimates [52]. Ninety-five percent predictive intervals were calculated for meta-analyses of ≥3 trials to incorporate existing heterogeneity and provide a range of possible effects for a future clinical setting, crucial for the correct interpretation of random-effects meta-analyses [52]. All *P*-values were two-sided (*α* = 0.05, except for between-study or between-subgroups heterogeneity tests in which *α* = 0.10). Since analyses were based on study-level summary statistics, and because primary studies did not adjust for clustering of multiple teeth within the same patient, no further adjustment for within-patient correlation was possible. All analyses were conducted by one author (S.N.P.) in R statistical software (version 4.4.1; R Foundation for Statistical Computing, Vienna, Austria), with an openly provided dataset through Zenodo (DOI: 10.5281/zenodo.17520391).

### Additional analyses, reporting bias assessment, and certainty assessment

Sources of heterogeneity were planned through subgroup/meta-regression analyses according to patient age, sex, or WSL severity for meta-analyses of at least five studies. Three sensitivity analyses were performed: (i) a *post hoc* sensitivity analysis by excluding the study of Gabr 2024 [53, 54] as their data differed considerably from all other studies; (ii) an *a priori* sensitivity analysis according to study design (randomized versus non-randomized study); and (iii) an *a priori* sensitivity analysis according to sample size (with the cut-off of 80 teeth/study that was used in the risk of bias assessment). Reporting biases (including the possibility of publication bias) were planned for meta-analyses with ≥10 studies with contour-enhanced funnel plots and Egger's test.

The GRADE (Grades of Recommendations, Assessment, Development, Evaluation) framework [55] was not applied, as current guidance mainly addresses comparative (two-group) meta-analyses, and there is limited consensus on how to grade evidence, particularly when it is derived solely from single-group pre-post data. Forest plots were augmented with contours denoting the magnitude of observed effects (Supplementary Material S1) to assess heterogeneity, clinical relevance, and imprecision [56].

## Results

### Study selection

**Figure 1:**
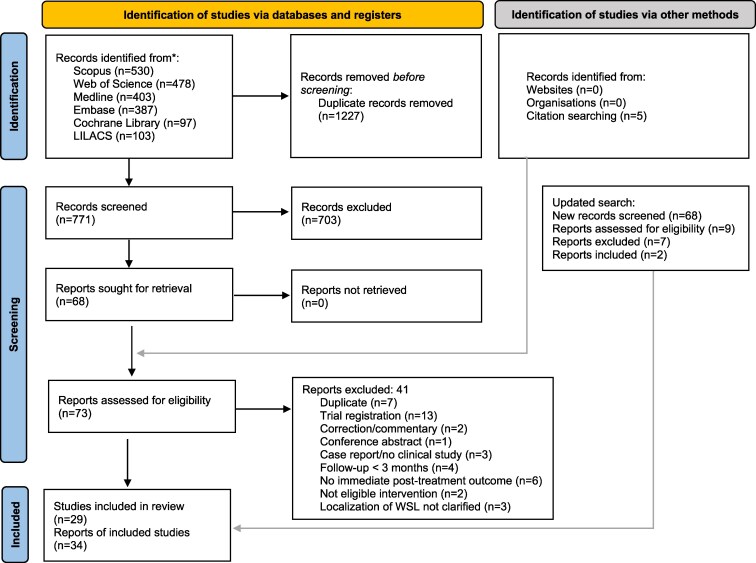
PRISMA 2020 flow diagram.

The initial electronic database search yielded 1998 records (Supplementary Material S4). After removing 1227 duplicate records, 771 unique records were screened for eligibility (Fig. 1). Additional five records were identified through manual searching. A total of 73 full text reports were assessed for eligibility. Corresponding authors of 11 studies were contacted to request additional data or datasets, and 7 responded, providing clarification of data (Supplementary Material S5) and three records were excluded because it could not be confirmed that they met the inclusion criteria. The later additional search identified 68 new records, and after screening for eligibility, 2 studies met the inclusion criteria. In total, 34 publications corresponding to 29 distinct clinical studies met the inclusion criteria. Of these, 22 studies were incorporated into the quantitative synthesis, while 7 could not be analyzed due to limited data (Supplementary Material S6). The majority were published as journal articles, with one being a preliminary report [57]. All studies were published in English, except for two in Chinese [58, 59] and one in Russian [60]. These articles were translated using digital translation tools and subsequently verified by bilingual colleagues to ensure accurate data extraction.

### Study characteristics

The characteristics of the included studies are summarized in Table 2. Among the 29 included studies, more than half (59%; 17/29) were randomized clinical trials comparing RI to at least one other treatment, with eight using a within-person randomization (split-mouth design). The remaining studies employed a prospective non-randomized design, with RI as the sole intervention (41%; 12/29). The reports were predominantly carried out in university clinics or hospitals across twelve countries: Turkey, China, Germany, Egypt, Iran, Italy, Bulgaria, India, Kosovo, Russia, Romania and Greece. In total, 544 patients (median 20 patients/study among the 26 studies reporting this) and 1495 teeth (median 45 teeth/study among the 26 studies reporting this) were treated with RI. The patients were predominantly adolescents and young adults, (range 7–40 years in 24 studies reporting this) with males representing 44% of all patients (from 18 studies reporting this). Most treated WSLs were idiopathic or post-orthodontic; however, two studies were conducted during orthodontic treatment with fixed appliances [71, 83]. In the included studies, RI was evaluated against a range of comparators, including SAE, topical fluorides, CPP-ACP, SAP, micro-abrasion, surface pre-reacted glass-ionomer filler, adhesive systems, composite resin sealants, or no comparator (Supplementary Material S7). Follow-up period varied, with 10 studies (34%) reporting up to 6 months, 14 studies (48%) up to 12 months, and only three studies providing longer follow-up of at least 2 years [75, 78, 84]. However, not all studies reported the same intermediate follow-up periods, even if the maximum follow-up period was the same. The most commonly assessed outcome was the change in L*a*b values (16/29 studies), followed by laser fluorescence (10/29 studies), International Caries Detection and Assessment System, Version II (ICDAS II) scores (8/29 studies), lesion grading (6/29 studies), WSL area (5/29 studies), patient-reported outcomes (4/29 studies), gray value (1/29 studies) and clinical parameters (1/29 studies).

**Table 2:** Characteristics of included studies.

Study (author year)	Country*; source	Design	Total patients (teeth) at baseline	Age in years	Male/female	Location of WSL	RI treatment	Follow-up after RI in months	Data collection	Outcomes
			*N*	Mean (range)	*N*	Max/man; ant/pre/mol; type of lesion	Patients (teeth) *N*			
Ciftci 2018 [61]	TUR; Uni	NrCS	66 (137) WSL: 39 (91)	NR (8–16)	27/41	Max; ant; post-ortho	21 (51)	0, 1, 3	CE; DD	ICDAS II; LF
Ding 2024 [62]	CHN; Uni, Hos	RCT_PAR_	33 (163)	NR (15–30)	NR	NR; ant/pre/mol; NR	10 (50)	0, 3, 6	CE; QLF	ICDAS II; LF
ElSayed 2021 [85]	EGY; Uni	RCT_WP_	12 (90)	18.3 (13–23.5)	NR	NR; ant/pre/mol; NR	12 (45)	0, 3, 6	CE; PROM	ICDAS II; PROM
Feng 2013 [58]	CHN; Uni	NrCS	8 (48)	NR (14–18)	NR	Max; ant; post-ortho	8 (48)	0, 6, 12	P	G; WSL area; gray value
Gabr 2022 [57]	EGY; Uni	RCT_PAR_	20 (40)	NR (20–40)	NR	Max; ant; NR	20 (20)	0, 3, 6, 12	DD	LF
Gabr 2024 [53]; Wakwak 2025 [54]	EGY; Uni	RCT_WP_	20 (80)	30.4 (20–40)	9/11	Max; ant; NR	20 (20)	0, 3, 6, 12	SP	L*a*b*
Gholami 2023 [103]	IRN; Uni	NrCS	18 (79) WSL (37)	NR (14–21)	6/12	NR; ant; post-ortho	NR (37)	0, 1, 3, 6	P; SP	L*a*b*
Giannetti 2018 [64]	ITA; Uni	NrCS	17 (38) Incipient caries (4) WSL (11)	14.7 (8–26)	7/10	Max; ant; idiopathic/post-ortho	NR [incipient caries (4)/WSL (11)]	0, 1, 12	P; PROM	G
Giray 2018 [35]	TUR; Uni	RCT_PAR_	23 (81)	10.8 (8–14)	13/10	Max; ant; idiopathic	12 (45)	0, 6	DD	LF
Giudice 2020 [65]	ITA; NR	NrCS	22 (22)	18.6 (12–29)	10/12	Max/man; ant/pre/mol; idiopathic/post-ortho	22 (22)	0, 12	SP	L*a*b*
Goda 2018 [63]	EGY; Uni	NrCS	20 (120)	NR (18-NR)	NR	Max; ant; NR	20 (60)	0, 3, 6, 12	SP	L*a*b*
Golia 2025 [66]	ROU; Uni	RCT_PAR_	47	NR (12-NR)	NR	Max/man; ant; idiopathic/post-ortho	23 (NR)	0, 3	P; PROM	G; PROM
Gu 2019 [67]	CHN; Uni	RCT_WP_	20 (128)	16.0 (12–19)	8/12	Max/man; ant; post-ortho	20 (64)	0, 6, 12	P; SP	L*a*b*; WSL area
Gözetici 2019 [68]	TUR; Uni	RCT_WP_	21 (84)	15.4 (12–21)	10/11	Max/man; ant/pre/molar; NR	21 (21)	0, 3, 6	CE; LF	ICDAS II; LF
Kabaktchieva 2014 [69]	BGR; NR	NrCS	6 (50)	NR (NR)	NR	Max/man; ant/pre/mol; NR	12 (90)	0, 6, 12	P(LIF)	G; LF
Kannan 2019 [70]	IND; Uni	RCT_WP_	12 (193)	NR (14–30)	5/7	Max/man; ant/pre/mol; post-ortho	6 (102)	0, 3, 6	DD; SP	L*a*b*; LF
Kashash 2024 [71]	DEU; Uni/PP	RCT_PAR_	38 (124)	14.3 (12–17)	21/15	Max/man; ant; during ortho	19 (60)	0, 1, 3, 6	P	ICDAS II; L*a*b*
Knaup 2023 [72]; Wierichs 2023a, c [36, 84]	DEU; Uni	NrCS	31 (239)	16.8 (11–36)	16/15	Max/man; ant/pre; post-ortho	31 (239)	0, 6, 12, 72	DD; P; QLF	G; ICDAS II; L*a*b*; LF; WSL area
Knösel 2013 [73]; Eckstein 2015 [74]; Knösel 2019 [75]	DEU; Uni	RCT_WP_	21 (231)	15.5 (12–19)	10/11	Max/man; ant; post-ortho	21 (114)	0, 1, 3, 6, 12, 24	SP	L*a*b*
Krasniqi 2024 [76]	XKX; Uni	RCT_PAR_	60 (173)	NR (8–15)	26/34	NR; ant; NR	30 (85)	0, 6	CE	ICDAS II
Krikheli 2020 [60]	RUS; Uni	RCT_PAR_	80 (NR)	NR (18–35)	NR	NR; ant/pre; NR	40 (NR)	0, 12	CE	CV
Ozgur 2023 [77]	TUR; Uni	NrCS	33 (100) WSL 27.4%	10.5 (7–15)	10/19	NR; ant; NR	NR (23)	0, 6	P; SP	L*a*b*
Puleio 2023 [78]	ITA; NR	NrCS	40 (40)	NR (NR)	NR	NR; ant/pre/mol; idiopathic/post-ortho	40 (40)	0, 12, 48	SP	L*a*b*
Rashid 2018 [79]	EGY; Uni	NrCS	15 (NR)	NR (NR)	NR	NR; ant; post-ortho	15 (NR)	0, 6, 12	P	L*a*b*
Rohym 2021 [80]	EGY; Uni	RCT_WP_	6 (72)	NR (15–25)	NR	Max/man; ant; post-ortho	6 (36)	0, 1, 3, 6, 12	DD; PROM; SP	L*a*b*; LF; PROM
Simon 2022 [81]	IND; Uni	RCT_PAR_	60 (60)	NR (13–15)	25/35	Max/man; ant; post-ortho	30 (30)	0, 3, 6, 12	P; SP	L*a*b*; WSL area
Tinghong 2019 [59]	CHN; Uni	RCT_PAR_	22 (88)	17 (16–26)	8/14	Max; ant; post-ortho	11 (44)	0, 1, 3, 6, 12	P; PROM	G; PROM; WSL area
Wierichs 2023b [83]	GRC; Hos	RCT_WP_	17 (76)	14.3 (12–18)	7/8	Max/man; ant/pre; during ortho	17 (38)	0, 6	P; DD	L*a*b*; ICDAS II; LF
Zaazou 2024 [82]	EGY; Hos	NrCS	57(>96)	16.1 (14–18)	19/30	NR; ant; NR	57 (>96)	0, 3, 6, 12	SP	L*a*b*

Ant, anterior; CE, clinical evaluation; CV, clinical values; DD, diagnodent; Hos, hospital; ICDAS, International Caries Detection and Assessment System; L*a*b*, CIE Commission Internationale de l’Éclairage L*a*b* color system, lightness (*L*), green–red axis (*a*), blue–yellow axis (*b*); LF, laser fluorescence; Man, mandibular; Max, maxillary; Mol, molar; NR, not reported; NrCS, non-randomized clinical study; ortho, orthodontic; PAR, parallel design; P, photos; P(LIF), light induced fluorescence, Soprolife camera; Pre, premolar; PP, private practice; PROM, patient-reported outcome measure; QLF, quantitative light-induced fluorescence; RI, resin infiltration; RCT, randomized controlled trial; SP, spectrophotometer; Uni, university; WSL, white spot lesion; WP, within-person randomization; * given with their ISO-3 code.

### Risk of bias of included studies

The risk of bias of included studies was assessed using a modified Joanna Briggs Institute’s tool for cohort studies and is summarized in Table 3, with the full Risk of Bias table provided in Supplementary Material S8. The most prominent limitation was the small sample size, as the majority of studies included fewer than 80 teeth treated with RI (76%; 22/29 studies). Only three studies conducted adjusted analyses to account for potential confounding factors [36, 65, 103]. Variation in RI protocols, particularly etching times, were noted (31%, 9/29 studies). Another limitation was that outcome measurements were not reported as being performed blinded (55%; 16/29 studies). In cases of dropouts during follow-up, differences in patient or WSL characteristics between different time points were not assessed (31%, 9/29 studies). In 17% of the reports, loss to follow-up exceeded 20%, yet only three reported reasons for attrition, and none adequately described strategies to address incomplete follow-up. Additionally, a substantial number of reports did not include follow-up periods of at least 1 year (41%; 12/29 studies). While some studies treated only one tooth with a WSL per patient, many included multiple WSLs per patient but failed to analyze or describe whether or how clustering was considered (76%; 22/29 studies). Finally, many studies (38%; 11/29 studies) either reported a conflict of interest or did not disclose whether one existed.

**Table 3:** Summary risk of bias of the included studies; modified JBI checklist for cohort studies.

Nr	Question	Total	Yes	No	NR	Partially
1	Sample size sufficient (≥80 teeth)	29	5 (17%)	22 (76%)	2 (7%)	—
2	Infiltration protocol described	29	27 (93%)	2 (7%)	—	—
3	Protocol similar for all WSLs	29	17 (59%)	9 (31%)	3 (10%)	—
4	Exclusion and inclusion criteria described	29	25 (86%)	3 (10%)	—	1 (3%)
5	Analysis of confounding factors	29	—	26 (90%)	—	3 (10%)
6	Outcomes measured in a reliable way	29	26 (90%)	2 (7%)	—	1 (3%)
7	Outcome assessors blinded	29	13 (45%)	16 (55%)	—	—
8	Same measurement at different time points	29	29 (100%)	—	—	—
9	Sufficient follow-up time	29	17 (59%)	12 (41%)	—	—
10	Difference in characteristics between time points	29	—	20 (69%)	9 (31%)	—
11	Loss of follow-up of concern	29	5 (17%)	24 (83%)	—	—
12	Reasons of loss at follow-up described	5^a^	1 (20%)	2 (40%)	—	2 (40%)
13	Strategies to address incomplete follow-up	5^a^	—	4 (80%)	—	1 (20%)
14	Appropriate statistical analysis	29	27 (93%)	2 (7%)	—	—
15	Clustering considered	29	7 (24%)	22 (76%)	—	—
16	Conflict of interest	29	3 (10%)	18 (62%)	8 (28%)	—

NR, not reported; WSL, white spot lesion.

^a^As 24 studies received a ‘No’ for question #11, they were therefore not rated on this item.

### Results of individual studies and data synthesis

The results of all included studies are given in the review’s openly available dataset (DOI: 10.5281/zenodo.17520391). The results of outcomes assessed by single studies and therefore not included in meta-analyses are given in Supplementary Material S9. Statistically significant (*P* < .05) differences from post-infiltration for single studies were seen at (i) 1 month [treatment efficiency (for WSL area ratio), WSL area, WSL area ratio]; (ii) 3 months [quantitative light-induced fluorescence of the WSL (*Q*_WSL_), treatment efficiency, WSL area, and WSL area ratio]; (iii) 6 months [QWSL, gray values, treatment efficiency, and WSL area]; (iv) 12 months [lightness of the WSL (*L*_WSL_), fluorescence of the WSL compared with SAE (*F*_WSL/SAE_), gray values, treatment efficiency, ICDAS II score, and visual score]; and (v) 72 months [WSL area, standardized color difference (Δ*E*_WSL/SAE_), *F*_WSL/SAE_, and visual score].

**Figure 2:**
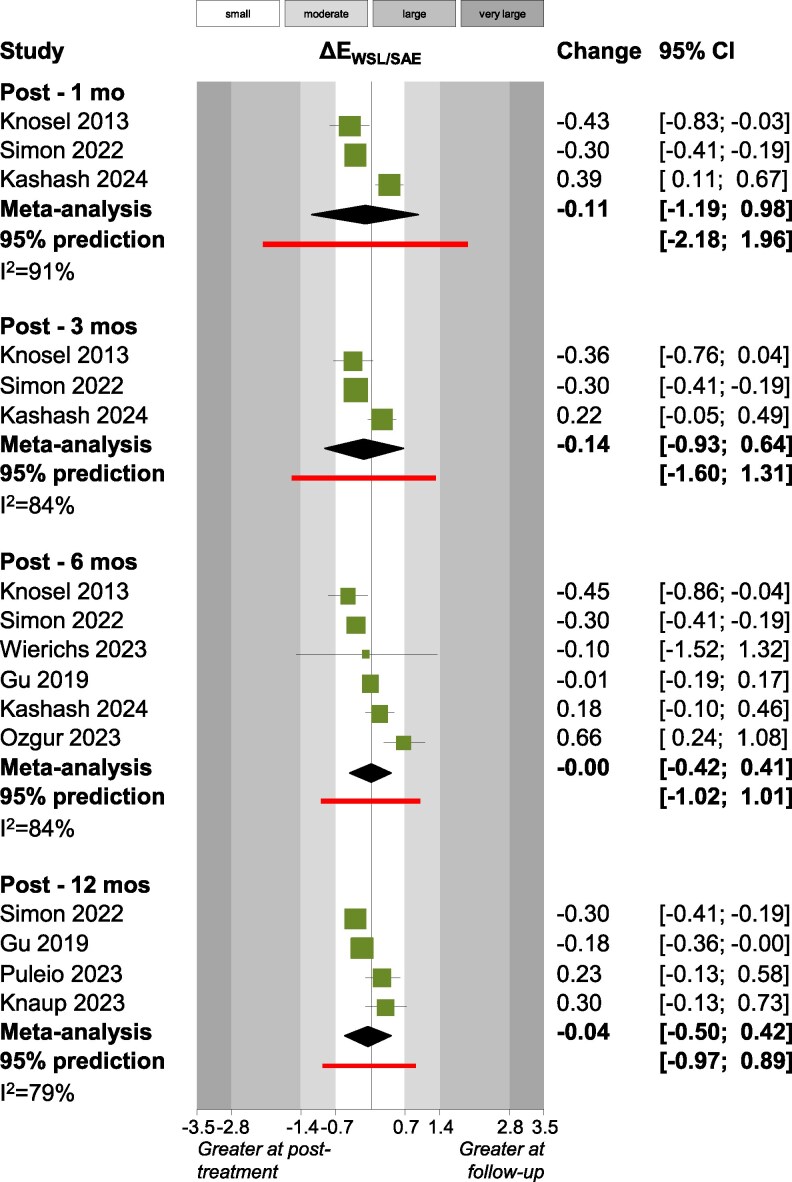
Forest plot for DE_WSL/SAE_.

**Figure 3:**
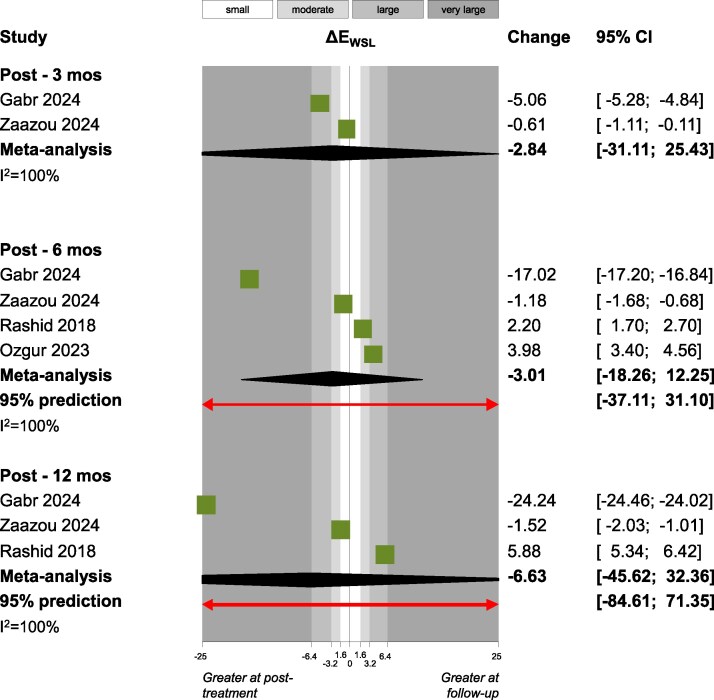
Forest plot for DE_WSL_.

**Figure 4:**
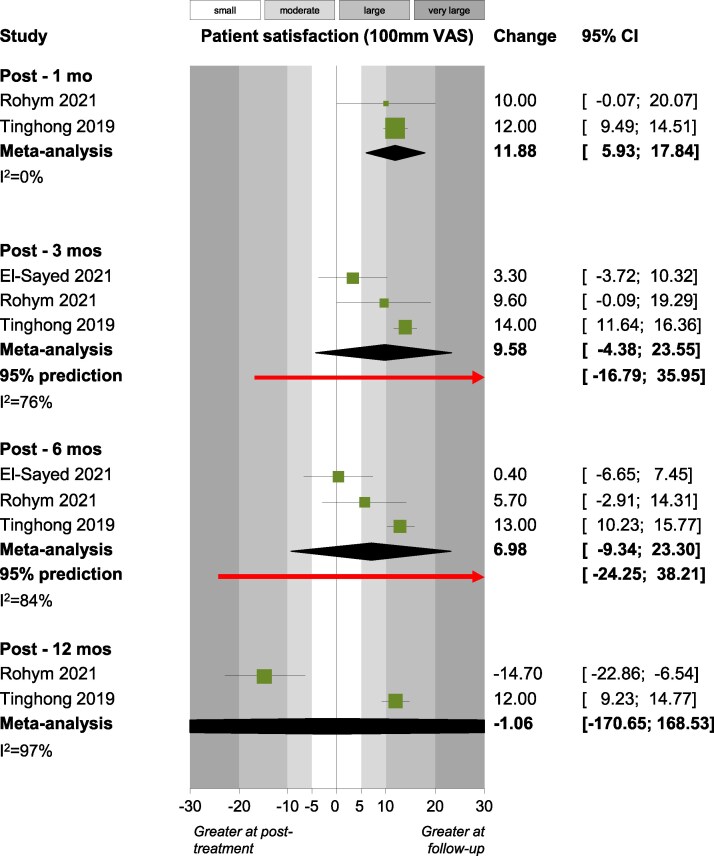
Forest plot for satisfaction_VAS_.

Outcomes that were assessed by two or more studies were synthesized, which led to very high heterogeneity and non-significant pooled estimates. This was mostly the case also for several crucial outcomes like Δ*Ε*_WSL/SAE_ (Fig. 2), Δ*Ε*_WSL_ (Fig. 3), patient satisfaction (Fig. 4) laser fluorescence, ICDAS II scores, WSL area and WSL area ratio (Table 4). However, there were some exceptions, where very small meta-analyses indicated statistically significant (*P* < .05) effects. Patient satisfaction [measured on a 100 mm Visual Analogue Scale (VAS)] improved post-infiltration at the first month (two studies; +11.9 mm; 95% CI +5.9 to +17.8 mm; *P* = .02), but this improvement reduced to non-significant levels at the third month (three studies; +9.6 mm; 95% CI −4.4 to 23.6 mm; *P* = .09), sixth month (three studies; +7.0 mm; 95% CI −9.3 to +23.3 mm; *P* = .20) and at the 12th month (two studies; −1.1 mm; 95% CI −170.7 to 168.5 mm; *P* = .94). Between the third and the sixth month post-infiltration, there were statistically significant improvements in the International Commission on Illumination (CIE) color space’s component for lightness (*L*) of the WSL (two studies; +1.2; 95% CI +0.1 to +2.3; *P* = .02) and the WSL’s fluorescence (two studies; +4.5; 95% CI +3.0 to 5.9; *P* < .001). Finally, statistically significant improvements were seen between the sixth and the 12th month post-infiltration for the CIE lightness component (*L*) of the WSL (two studies; +0.4; 95% CI +0.1 to +0.8; *P* = .01) and the color difference (CIE component *a*) between the WSL and the SAE (two studies; +0.6; 95% CI 0 to +1.2; *P* = .03).

**Table 4:** Meta-analyses performed.

Nr	Outcome	Time period	Studies	Change (95% CI)	*P*	Tau^2^ (95% CI)	*I* ^2^ (95% CI)	Prediction
1	DE_WSL/SAE_	Post-1 month	3	−0.11 (−1.19, 0.98)	.71	0.17 (0.03, 7.65)	91% (76%, 96%)	−2.18, 1.96
2	LF	Post-1 month	2	−0.12 (−0.42, 0.19)	.45	0 (–)	0% (–)	—
3	Satisfaction_VAS_	Post-1 month	2	11.88 (5.93, 17.84)	.02	0 (–)	0% (–)	—
4	*L*_WSL_	Post-3 months	2	0.87 (−0.71, 2.44)	.28	1.20 (–)	93% (–)	—
5	DE_WSL_	Post-3 months	2	−2.84 (−31.11, 25.43)	.42	9.86 (–)	100% (–)	—
6	DE_WSL/SAE_	Post-3 months	3	−0.14 (−0.93, 0.64)	.51	0.08 (0.01, 4.00)	84% (51%, 95%)	−1.60, 1.31
7	LF	Post-3 months	3	0.35 (−1.81, 2.50)	.75	3.08 (0.53, >100)	98% (96%, 99%)	−25.97, 26.66
8	ICDAS II	Post-3 months	2	0.04 (−0.09, 0.18)	.53	0.01 (–)	55% (–)	—
9	Satisfaction_VAS_	Post-3 months	3	9.58 (−4.38, 23.55)	.09	25.53 (0.94, >100)	76% (22%, 93%)	−16.79, 35.95
10	*L*_WSL_	Post-6 months	4	1.17 (−1.67, 4.01)	.28	3.07 (0.90, 43.89)	97% (94%, 98%)	−7.30, 9.63
11	*a*_WSL_	Post-6 months	3	0.09 (−0.51, 0.69)	.76	0.22 (0.03, 8.37)	89% (71%, 96%)	−7.02, 7.20
12	*b*_WSL_	Post-6 months	3	1.43 (−0.21, 3.08)	.08	1.79 (0.24, 86.16)	85% (55%, 95%)	−18.64, 21.51
13	DE_WSL_	Post-6 months	4	−3.01 (−18.26, 12.25)	.57	91.88 (29.46, >100)	100% (NC)	−37.11, 31.10
14	DE_WSL/SAE_	Post-6 months	6	0 (−0.42, 0.41)	.98	0.13 (0.03, 0.88)	84% (67%, 92%)	−1.02, 1.01
15	LF	Post-6 months	4	2.20 (−3.30, 7.71)	.29	11.73 (3.50, >100)	99% (99%, 99%)	−10.05, 14.45
16	ICDAS II	Post-6 months	3	−0.26 (−0.80, 0.28)	.35	0.22 (0.05, 9.11)	97% (93%, 98%)	−7.19, 6.68
17	Satisfaction_VAS_	Post-6 months	3	6.98 (−9.34, 23.30)	.20	36.94 (4.17, >100)	84% (50%, 95%)	−24.25, 38.21
18	WSL area ratio	Post-6 months	2	−4.04 (−9.29, 1.20)	.13	13.23 (–)	92% (–)	—
19	DE_WSL_	Post-12 months	3	−6.63 (−45.62, 32.36)	.54	246.34 (66.75, >1000)	100% (NC)	−84.61, 71.35
20	DE_WSL/SAE_	Post-12 months	4	−0.04 (−0.50, 0.42)	.80	0.06 (0.01, 1.20)	79% (42%, 92%)	−0.97, 0.89
21	LF	Post-12 months	2	4.98 (−2.52, 12.48)	.19	28.15 (–)	96% (–)	—
22	Satisfaction_VAS_	Post-12 months	2	−1.06 (−170.65, 168.53)	.94	346.78 (–)	97% (–)	—
23	WSL area	Post-12 months	2	−1.26 (−3.81, 1.28)	.33	3.26 (–)	97% (–)	—
24	WSL area ratio	Post-12 months	2	−3.99 (−9.16, 1.18)	.13	12.82 (–)	92% (–)	—
25	DE_WSL/SAE_	1–3 months	3	0 (−0.06, 0.05)	.87	0 (0, 0.58)	0% (0%, 90%)	−0.35, 0.34
26	LF	1–3 months	2	−1.17 (−4.08, 1.75)	.43	3.66 (–)	79% (–)	—
27	Satisfaction_VAS_	1–3 months	2	0.78 (−1.57, 3.13)	.52	1.47 (–)	51% (–)	—
28	*L*_WSL_	3–6 months	2	1.19 (0.12, 2.26)	.02	0.51 (–)	85% (–)	—
29	DE_WSL_	3–6 months	2	−6.27 (−17.43, 4.89)	.27	64.82 (NC)	100% (100%, 100%)	—
30	DE_WSL/SAE_	3–6 months	3	0 (−0.06, 0.05)	.90	0 (0, 0.06)	0% (0%, 90%)	−0.36, 0.35
31	LF	3–6 months	2	4.45 (2.95, 5.94)	<.001	0.67 (–)	36% (–)	—
32	Satisfaction_VAS_	3–6 months	3	−2.13 (−4.33, 0.08)	.05	0.27 (0, 82.85)	0% (0%, 90%)	−17.85, 13.60
33	*L*_WSL_	6–12 months	2	0.42 (0.08, 0.76)	.01	0 (–)	0% (–)	—
34	*L*_WSL/SAE_	6–12 months	2	−0.06 (−0.53, 0.41)	.81	0 (–)	0% (–)	—
35	*a*_WSL/SAE_	6–12 months	2	0.61 (0.03, 1.18)	.03	0.11 (–)	64% (–)	—
36	*b*_WSL/SAE_	6–12 months	2	0.76 (0, 1.53)	.05	0.14 (–)	45% (–)	—
37	DE_WSL_	6–12 months	3	−1.30 (−7.54, 4.94)	.68	30.36 (8.20, >100)	100% (–)	−82.16, 79.57
38	DE_WSL/SAE_	6–12 months	4	−0.11 (−0.41, 0.19)	.34	0.01 (0, 1.47)	59% (0%, 86%)	−0.76, 0.54
39	LF	6–12 months	2	−0.06 (−5.40, 5.27)	.98	13.71 (–)	92% (–)	—
40	Satisfaction_VAS_	6–12 months	2	−10.61 (−29.62, 8.40)	.27	184.16 (–)	98% (–)	—
41	WSL area ratio	6–12 months	2	0.04 (−0.48, 0.56)	.89	0 (–)	0% (–)	—

DE, *L*, *a*, *b*, CIE Commission Internationale de l’Éclairage L*a*b* color system, color difference, lightness (*L*), green–red axis (*a*), blue–yellow axis (*b*); CI, confidence interval; ICDASII, International Caries Detection and Assessment System II; LF, laser fluorescence; NC, non-calculable; SAE, sound adjacent enamel; VAS, visual analogue scale; WSL, white spot lesion.

### Additional analyses

Several additional analyses (subgroup analyses and meta-regressions) were initially planned but could not be performed due to the small numbers of studies included in the meta-analyses (<5 studies). In the only meta-analysis with at least five studies (Δ*Ε*_WSL/SAE_ changes post-infiltration to sixth month), no subgroup/meta-regression analyses could be performed since inadequate data were reported.

Careful consideration of the included studies indicated that the results of one study [53, 54] differed considerably from the results of all other studies. However, communication with the authors did not indicate any different measurement methods used by the authors (Supplementary Material S5). As such, a *post hoc* sensitivity analysis was considered, by excluding the present study from the analyses. This sensitivity analysis indicated robustness (Supplementary Material S10–S12), since its results were virtually identical to the original analysis.

Sensitivity analyses according to study design and sample size adequacy are seen in Supplementary Material S13. For the only meta-analysis with at least five studies (Δ*Ε*_WSL/SAE_; post-infiltration to sixth month) significant differences were seen according to study design. However, this was due to the fact that the single non-randomized study found significant post-infiltration Δ*Ε*_WSL/SAE_ differences at 6 months, while the remaining five randomized studies did not agree, and the latter was judged to be more robust. No significant differences were seen between studies with adequate sample size (more than 80 teeth) or those without (80 teeth or less; *P* > .10).

The GRADE framework was not used to assess the certainty of evidence, since no clear guidance exists for assessing changes based solely on single-group pre- and post-data.

Similarly, the risk of reporting biases (including the risk for publication bias) could not be formally assessed, since <10 studies were included in all meta-analyses.

## Discussion

### Evidence in context

In this systematic review, the existing evidence regarding the effect of RI on WSLs has been critically summarized. The objective was to evaluate outcomes following RI of WSLs at different time points. Data from twenty-nine distinct clinical studies, with 544 patients and 1495 teeth were included, assessing several clinical outcomes such as L*a*b values, laser fluorescence, WSL area and ICDAS II, as well as patient-reported outcomes related to satisfaction. However, the large variation in measurement methods and outcomes resulted in only a few studies being included in each meta-analysis. Moreover, very high between-study heterogeneity was observed for most of the outcomes. These findings potentially suggest that no standardized or reliable protocol currently seems to exist for evaluating the effects of RI on WSLs.

Several studies compared RI with other treatments and concluded that RI was more effective treatment for WSLs than fluoride varnish [35, 61, 62, 68, 71, 76, 83], CPP-ACP [62], micro abrasion [59, 67, 85], surface pre-reacted glass-ionomer [53], dental adhesive [63], SAP [68] and fluoride releasing coating [70, 80]. However, as the present review aimed to evaluate outcomes solely with RI, no comparative analyses with other treatment modalities were performed.

A variety of outcome measures were reported across the included studies. The most common outcome measure was the change in L*a*b* values, a standardized color measurement system that quantifies color objectively and numerically. The CIE L*a*b* color space, developed by the CIE Commission Internationale de l’Éclairage (International Commission on Illumination) [86], is a three-dimensional model in which L* represents lightness [ranging from 0 (black) to 100 (white)], a* represents the red–green axis (positive values indicate red and negative values indicate green), and b* represents the yellow–blue axis (positive values indicate yellow and negative values indicate blue). When two measurements of L*a*b* values are available, often obtained using a spectrophotometer, the Δ*E* can be calculated. Individuals with average color-matching ability can perceive a Δ*E* of ∼2.5–3.5 units, while a Δ*E* value of 1 is visually detectable in ∼50% of cases using a spectrophotometer, and Δ*E* value >2 is detectable in 100% of all cases [87, 88]. Within the oral environment, a mean *E* of ∼3.7 is perceived as an acceptable color match [89]. The most common approach among the included studies involved comparing the L*a*b values of treated WSLs with those of SAE on the same tooth [67, 71–73, 77, 78, 81, 83]. In all studies, the post-treatment Δ*Ε*_WSL/SAE_ values decreased relative to baseline, indicating that treated WSLs became more similar in color to the surrounding enamel. When comparing immediate post-treatment measurements with subsequent follow-ups, individual studies reported both negative changes (further reduction in color difference over time) and positive changes (increased color difference over time). The pooled mean from the meta-analysis showed a very small negative effect across time points; however, this was not statistically significant and is unlikely to be clinically relevant. Therefore, these findings suggest that Δ*Ε*_WSL/SAE_ remains consistent across different time points.

An alternative approach to L*a*b* measurement involved measuring color directly on the WSL, at a single point [53, 77, 79, 82]. In this method, the Δ*E* value obtained shortly after treatment is compared with the baseline measurement, and the pooled mean represents the change in Δ*E* between post-treatment and follow-up measurements. Considerable variability was observed among the individual studies, and the pooled mean of change indicates a Δ*E* exceeding the threshold for visual detection (Δ*E* > 2.5) [87, 88], suggesting that this outcome varies over time. However, the difference was not statistically significant, and the wide 95% CI implies substantial heterogeneity and an overall uncertain trend.

Three studies investigating PROMs using VAS for satisfaction were included in the analysis [59, 80, 85]. The pooled mean indicated a positive trend during the first months (one, three, and six) after treatment, with patients reporting greater satisfaction with their dental appearance; however, only the improvement at the 1-month follow-up was statistically significant. Furthermore, at 12 months, satisfaction showed a small decline, though, this change was not statistically significant. Similarly, one study on dental fluorosis treatment found no improvement in satisfaction up to 6 months after RI alone, while prior bleaching significantly enhanced patient satisfaction [90]. Overall, the results from this systematic review suggest that PROMs remain largely underexplored in the context of WSL treatment. Nevertheless, previous studies have shown that tooth color and color discrepancies influence satisfaction with dental appearance, and that individuals who are dissatisfied are more likely to seek color corrective treatments [18, 91]. This highlights the need for further research on patient-reported outcomes to ensure that clinical success aligns with patients’ perception and expectations of treatment.

Laser fluorescence is another method to investigate the enamel following RI, as it assesses changes in enamel mineral content. Lower fluorescence readings indicate improved mineralization and lesion stability, whereas higher readings suggest ongoing demineralization [92]. A study evaluating the validity of laser fluorescence for orthodontic WSLs found that visual examination demonstrated higher accuracy for detecting early WSLs, and no difference was observed for more severe lesions [93]. The meta-analysis revealed a slight increase in fluorescence values over time, possibly reflecting a minor reduction in enamel mineral or partial relapse; however, these changes were not statistically significant.

The ICDAS II outcome originates from a caries classification system [94] rather than serving as a direct outcome measure. The meta-analysis did not demonstrate a statistically significant reduction in ICDAS II scores [61, 76, 83, 85]. The assessment of lesions localized to the enamel corresponds to the early ICDAS codes and might therefore not be sensitive enough to detect subtle changes. In addition, the evaluation is operator-dependent, which may introduce subjectivity and variability in the results [94, 95].

Two different approaches were used to assess the area of WSLs, either as a reduction in lesion size [72, 81] or as a ratio relative to the total surface area of the tooth [59, 67]. The studies varied in software used for area calculations, and since these assessments often involves subjective interpretation, inter-rater reliability may be questionable [95]. Although both approaches showed a decreasing trend, the pooled mean at available time points were not statistically significant, warranting cautious interpretation of the findings.

To address heterogeneity among the included studies, a 95% prediction interval was calculated for all the outcomes with ≥3 studies, estimating the range within which the true effect for a future study may fall. This provides a more clinically meaningful interpretation of random-effects meta-analysis results [96]. For all outcomes included in the meta-analysis, the 95% prediction intervals showed wide variation, ranging from negative to positive values, indicating that future studies may vary substantially and that the true effects remain uncertain. However, for Δ*Ε*_WSL/SAE_, the predictive interval at 6 and 12 months were narrow, indicating that future studies are likely to find only minimal changes, suggesting consistent effects over these follow-up periods.

### Strengths and limitations

The present review has several strengths, including its pre-registration [97], an extensive and unrestricted literature search including gray literature [98] and the application of statistical and meta-analytical methods for data synthesis [99]. In addition, the dataset was made transparently available to ensure reproducibility [100]. Another strength was the careful examination of study-level consistency. When one study showed results that diverged markedly from the others, direct contact with the authors confirmed that no alternative measurement methods had been used. A subsequent *post hoc* sensitivity analysis excluding this study demonstrated the robustness of the overall findings, as the overall results remained essentially unchanged.

The current evidence base is constrained by several notable limitations. Since most primary studies did not adjust for within-patient clustering, the effective sample sizes may be overestimated and the corresponding standard errors underestimated, resulting in too narrow CIs in the meta-analyses. Another limitation is that most of the available follow-up periods represent only short- to intermediate-term observations, whereas true long-term data in dentistry typically extends beyond 5–10 years [101, 102]. As only a few studies have followed patients over extended periods, no conclusions about the long-term durability of RI can be drawn.

Furthermore, the substantial heterogeneity observed across studies may be attributed to differences in study design, as the included studies comprised both randomized controlled trials (parallel-group or split-mouth) and non-randomized clinical studies without control groups. In many studies, neither patients, operators, nor outcome assessors were blinded, which may have introduced bias and contributed to heterogeneity. Variation in follow-up also likely influence outcomes, as RI may evolve over time due to color change and discoloration; however, each time point was analyzed separately to minimize this effect. Furthermore, all studies utilized the same commercial RI material, Icon® (DMG, Hamburg, Germany). Nevertheless, variations in application protocols were observed, particularly regarding the number of etching cycles with hydrochloric acid. Several studies reported multiple etching steps based on visual assessment of the enamel after alcohol drying [36, 64, 65, 67, 71–75, 78, 82–84]. Although this approach aligns with manufacturer recommendations, it may have influenced treatment outcomes compared with studies applying fewer times. Moreover, many included studies had relatively small sample size of both patients and teeth, increasing random variation and the risk for type II errors. Seven studies could not be included in the meta-analysis because of missing data, specifically the absence of immediate post-treatment data or missing mean and standard deviation values [62–64, 66, 69], lack of relevant outcome [60], or analyses combining WSLs with other defect of the enamel [103].

A limitation of this review is that additional analyses, such as subgroup analyses and meta-regressions, could not be performed because most meta-analyses included fewer than five studies. To ensure meaningful subgroup analyses and stable estimates, a minimum of five studies per comparison is generally needed [48]. In the only meta-analysis meeting this threshold (Δ*Ε*_WSL/SAE_, changes post-infiltration to 6 months), insufficient data reporting precluded further analysis. Even though, differences in RI protocols, and the inclusion of two studies performed during ongoing orthodontic treatment [71, 83] underscore the methodological variability within the evidence base.

These limitations highlight the absence of standardized and reliable protocol for assessing the effects of RI on WSLs and emphasize the need for more consistent methodology and detailed reporting in future studies to facilitate more robust secondary analyses. Furthermore, all included studies were non-randomized or single-arm data derived from randomized controlled trials, reflecting the available evidence within the scope of this review. Although this approach allowed inclusion of the full body of longitudinal data, it should be considered when interpreting the certainty and strength of the evidence.

Finally, the present review included all available post-infiltration time points, as no guidance currently exists regarding follow-up after RI, which may increase the risk of false-positive findings. It is important to note that (i) multiplicity in meta-analyses is partially controlled by protocol-based pre-specification of analyses [48], (ii) meta-analyses typically emphasize effect sizes and CIs rather than hypothesis testing, and (iii) adjusting for all *P*-values in systematic reviews can be impractical or overly conservative [49, 104], particularly as such analyses are often exploratory or hypothesis-generating. However, future studies would benefit from standardized WSL treatment protocols with RI and predefined follow-up time points within a core outcome set, which may reduce unnecessary testing.

## Conclusion

Existing evidence indicates that, for most outcomes and time points, RI as treatment for WSLs shows highly inconsistent effects, resulting in substantial heterogeneity, and no consistent pattern for time changes. This inconsistency was observed across both clinical and patient-reported outcomes, and the limited number of studies with extended follow-up restricts the ability to draw firm conclusions regarding effects at different time points. Together, these findings highlight the need for larger, standardized studies with longer follow-up periods to better evaluate the effects of RI over time.

## Supplementary Material

cjag021_Supplementary_Data

## Data Availability

Dataset for all analyses is openly available for all at Zenodo (DOI: 10.5281/zenodo.17520391).
