# Supplementary material for: Outcomes of resin infiltration for white spot lesions at different time points: a systematic review and meta-analysis
Source: Eur J Orthod. 2026 May 15;48(3):cjag021. doi: 10.1093/ejo/cjag021 (PMC13178833; doi:10.1093/ejo/cjag021)
Supplement: cjag021_Supplementary_Data [file cjag021_supplementary_data.docx]

**Supplementary Material**

**Supplementary Material 1**

***Additional methods***

- The study by Ozgur et al. (2023) had merged data for hypomineralization and WSLs; however, the outcomes were presented separately in one of the figures, and the lesion types showed no substantial differences across time points. The study was therefore included in the analysis.
- Rationale for thresholds applied in the Risk of Bias assessment
  - When assessing whether the sample size was sufficient, guidance was derived from the sample size calculation described by Knösel et al. (2013). In that study, the authors performed a formal power analysis (ΔE threshold value = 3.0; SD = 4.5; α = 0.05; power = 80%), concluding that approximately 20 participants, each contributing an average of 8 teeth, distributed between two interventions, were needed. Based on this, studies including ≥ 80 teeth were considered to have an adequate sample size and were judged at low risk of bias for sample size sufficiency.
  - To ensure consistency in assessing study quality, we applied predefined thresholds for acceptable participant loss during follow-up. For studies with follow-up periods ≤12 months, losses ≤20% were considered acceptable. For studies with follow-up periods >12 months, losses ≤30% were considered acceptable. In cases where attrition exceeded these thresholds, potential bias related to loss to follow-up was noted.
- When results were provided as data post-infiltration and data at a follow-up time point a Pre/Post correlation of 0.75 (back-calculated from studies providing appropriate data) was used to calculate follow-up-induced changes, according to the methods described in the Cochrane Handbook (Higgins et al., 2022).
- The Hartung-Knapp correction (Langan et al., 2019) was used for meta-analyses with >3 studies and for all cumulative meta-analyses provided in forest plots. This does not follow guidance indicating that this approach should be considered for meta-analyses with at least 5 studies (Veroniki et al., 2019), but this was chosen in an attempt to be more conservative, due to the large number of performed comparisons.
- Contour-enhanced forest plots were constructed based on the Standard Deviation (SD) of each outcome, as reported in the included studies of each meta-analysis at the post-infiltration stage (averaged). Conventional cut-offs were used for small effects (half an SD), moderate effects (1 SD), and large effects (2 SDs).

***Changes to initial protocol***

- The original research question was stated as: “What is the long-term stability of the effects of resin infiltration on white spot lesions?”. However, it was considered that the term “long-term” could be misinterpreted in relation to the predefined inclusion criteria, which permitted studies with follow-up periods as short as 3 months. To ensure alignment between the research question and the temporal scope of the included studies, the research question was revised to: “What are the effects of resin infiltration on white spot lesions at different time points?”. This modification clarifies that the review evaluates outcomes across the range of reported follow-up periods rather than exclusively long-term effects.
- According to the initial protocol, the risk of bias was to be assessed using the Cochrane RoB 2 tool for comparative randomized trials, the ROBINS-I tool for non-randomized comparative studies, and Joanna Briggs Institute (JBI) checklist for cohort studies for single-group clinical studies. However, since the analysis focused exclusively on patients treated with resin infiltration and thus considered these as a single-group cohort, a modified JBI checklist for cohort studies was used. Questions were adapted to ensure clinical relevance and to reflect factors directly related to the research question.
- Although the initial protocol planned to assess the quality of evidence using the GRADE approach and summarize it in Summary of Findings tables, this was not applied, as no clear guidance exists for evaluating changes based solely on one group from randomized trials.
- Several subgroup/meta-regression analyses were planned (subsets according to the patient characteristics (patient age, sex, ethnicity, jaw, tooth type, level of oral hygiene / subsets according to type of interventions / subsets according to the observation duration / subsets according to the measurement method), but could ultimately not be performed for most of the cases, only one meta-analysis included at least 5 studies and incomplete reporting did not allow for these analyses.

**References to Supplementary Material 1**

Ozgur B, Unverdi GE, Ertan AA, Cehreli ZC. Effectiveness and Color Stability of Resin Infiltration on Demineralized and Hypomineralized (MIH) Enamel in Children: Six-month Results of a Prospective Trial. Operative dentistry. 2023;48(3):258-67.

Knösel M, Eckstein A, Helms HJ. Durability of esthetic improvement following Icon resin infiltration of multibracket-induced white spot lesions compared with no therapy over 6 months: a single-center, split-mouth, randomized clinical trial. Am J Orthod Dentofacial Orthop 2013;144(1):86-96.

Higgins JPT, Thomas J, Chandler J, Cumpston M, Li T, Page MJ, Welch VA (editors). Cochrane Handbook for Systematic Reviews of Interventions version version 6.5 (updated August 2024). Cochrane, 2024. Available from [www.training.cochrane.org/handbook](http://www.training.cochrane.org/handbook).

Langan D, Higgins JPT, Jackson D, Bowden J, Veroniki AA, Kontopantelis E, Viechtbauer W, Simmonds M. A comparison of heterogeneity variance estimators in simulated random-effects meta-analyses. Res Synth Methods 2019;10(1):83-98.

Veroniki AA, Jackson D, Bender R, Kuss O, Langan D, Higgins JPT, Knapp G, Salanti G. Methods to calculate uncertainty in the estimated overall effect size from a random-effects meta-analysis. Res Synth Methods. 2019;10(1):23-43.

**Supplementary Material 2.** Databases and search terms

Scopus

| Interface: Elsevier  Date of Search: 2024-12-05  Number of hits: 530  Comment: | Field labels   - TITLE = title - ABS = abstract - KEY = keywords - W/x = within x words, regardless of order - * = truncation of word for alternate endings |
| --- | --- |
| \| **#** \| **Searches** \| **Results** \| \| --- \| --- \| --- \| \| 10 \| 3 AND 9 \| 530 \| \| 9 \| 4 OR 5 OR 6 OR 7 OR 8 \| 40928 \| \| 8 \| TITLE-ABS-KEY ((initial OR incipient OR "early stage" OR "early-stage") W/3 enamel W/3 lesion*) \| 275 \| \| 7 \| TITLE-ABS-KEY (incipient W/3 (caries OR carious)) \| 548 \| \| 6 \| TITLE-ABS-KEY (("early stage" OR "early-stage") W/3 (caries OR carious)) \| 221 \| \| 5 \| TITLE-ABS-KEY (initial W/3 (caries OR carious)) \| 936 \| \| 4 \| TITLE-ABS-KEY (deminerali* OR decalcifi* OR "white spot*" OR "white-spot") \| 39794 \| \| 3 \| 1 OR 2 \| 22052 \| \| 2 \| TITLE-ABS-KEY (icon) \| 20620 \| \| 1 \| TITLE-ABS-KEY (resin* W/3 infiltra*) \| 1700 \| | |

Cochrane Library

| Interface: Cochrane Library  Date of Search: 2024-12-05  Number of hits: 97  Comment: If using wildcards, use NEXT operator, phrase search does not support the use of wildcards. | Field labels   - ti,ab,kw = title, abstract and author keywords - NEAR/x = within x words, regardless of order - * = truncation of word for alternate endings |
| --- | --- |
| \| **#** \| **Searches** \| **Results** \| \| --- \| --- \| --- \| \| 10 \| #3 AND #9 \| 97 \| \| 9 \| #4 OR #5 OR #6 OR #7 OR #8 \| 2513 \| \| 8 \| ((initial OR incipient OR “early stage” OR “early-stage”) NEAR/3 enamel NEAR/3 lesion*):ti,ab,kw \| 49 \| \| 7 \| (incipient NEAR/3 (caries OR carious)):ti,ab,kw \| 109 \| \| 6 \| ((“early stage” OR “early-stage”) NEAR/3 (caries OR carious)):ti,ab,kw \| 7 \| \| 5 \| (initial NEAR/3 (caries OR carious)):ti,ab,kw \| 199 \| \| 4 \| (deminerali* OR decalcifi* OR white NEAR spot* OR white NEAR spot*):ti,ab,kw \| 2274 \| \| 3 \| #1 OR #2 \| 763 \| \| 2 \| (ICON):ti,ab,kw \| 656 \| \| 1 \| (resin* NEAR/3 infiltra*):ti,ab,kw \| 168 \| | |

Web of Science Core Collection

| Interface: Clarivate Analytics  Date of Search: 2024-12-05  Number of hits:478 | Field labels   - TS/Topic = title, abstract, author keywords and Keywords Plus - NEAR/x = within x words, regardless of order - * = truncation of word for alternate endings |
| --- | --- |
| \| **#** \| **Searches** \| **Results** \| \| --- \| --- \| --- \| \| 10 \| #3 AND #9 \| 478 \| \| 9 \| #4 OR #5 OR #6 OR #7 OR #8 \| 29256 \| \| 8 \| TS=((initial OR incipient OR "early stage" OR "early-stage") NEAR/3 enamel NEAR/3 lesion*) \| 183 \| \| 7 \| TS=(incipient NEAR/3 (caries OR carious)) \| 377 \| \| 6 \| TS=(("early stage" OR "early-stage") NEAR/3 (caries OR carious)) \| 55 \| \| 5 \| TS=(initial NEAR/3 (caries OR carious)) \| 577 \| \| 4 \| TS=(deminerali* OR decalcifi* OR "white spot*" OR "white-spot") \| 28569 \| \| 3 \| 1 OR 2 \| 12774 \| \| 2 \| TS=(ICON) \| 11665 \| \| 1 \| TS=(resin* NEAR/3 infiltra*) \| 1307 \| | |

Medline
Ovid MEDLINE(R) and In-Process, In-Data-Review & Other Non-Indexed Citations

| Interface: Ovid  Date of Search: 2024-12-05 Number of hits: 403  Comment: In Ovid, two or more words are automatically searched as phrases; i.e. no quotation marks are needed | Field labels   - exp/ = exploded MeSH term - / = non exploded MeSH term - .ti,ab,kf. = title, abstract and author keywords - adjx = within x words, regardless of order - * = truncation of word for alternate endings - /FREQ=n occurrence of a term, records only retrieved if  term/s occur at least n number of times |
| --- | --- |
| \| **#** \| **Searches** \| **Results** \| \| --- \| --- \| --- \| \| 10 \| 3 AND 9 \| 403 \| \| 9 \| 4 OR 5 OR 6 OR 7 OR 8 \| 22704 \| \| 8 \| ((initial OR incipient OR early stage OR early-stage) adj3 enamel adj3 lesion*).ti,ab,kf \| 205 \| \| 7 \| (incipient adj3 (caries OR carious)).ti,ab,kf \| 424 \| \| 6 \| ((early stage OR early-stage) adj3 (caries OR carious)).ti,ab,kf \| 54 \| \| 5 \| (initial adj3 (caries OR carious)).ti,ab,kf \| 660 \| \| 4 \| (deminerali* OR decalcifi* OR white spot* OR white-spot).ti,ab,kf \| 21903 \| \| 3 \| 1 OR 2 \| 2612 \| \| 2 \| ICON.ti,ab,kf \| 2001 \| \| 1 \| (resin* adj3 infiltra*).ti,ab,kf \| 813 \| | |

Embase

| Interface: Elsevier  Date of Search: 2024-12-05  Number of hits: 387  Comment: use ' for phrase searching | Field labels   - /exp = exploded Emtree term - /de = non exploded Emtree term - :ti,ab,kw. = title, abstract and author keywords - NEAR/n = within x words, regardless of order - * = truncation of word for alternate endings - * = variable wildcard, i.e one or more letters - ' = phrase |
| --- | --- |
| \| **#** \| **Searches** \| **Results** \| \| --- \| --- \| --- \| \| 10 \| 3 AND 9 \| 387 \| \| 9 \| 4 OR 5 OR 6 OR 7 OR 8 \| 25958 \| \| 8 \| ((initial OR incipent OR 'early stage' OR 'early-stage') NEAR/3 enamel NEAR/3 lesion*):ti,ab,kw \| 193 \| \| 7 \| (incipient NEAR/3 (caries OR carious)):ti,ab,kw \| 403 \| \| 6 \| (('early stage' OR 'early-stage') NEAR/3 (caries OR carious)):ti,ab,kw \| 54 \| \| 5 \| (initial NEAR/3 (caries OR carious)):ti,ab,kw \| 653 \| \| 4 \| (deminerali* OR decalcifi* OR 'white spot*' OR 'white-spot'):ti,ab,kw \| 25152 \| \| 3 \| 1 OR 2 \| 3650 \| \| 2 \| ICON:ti,ab,kw \| 3060 \| \| 1 \| (resin* NEAR/3 infiltra*):ti,ab,kw \| 775 \| | |

LILACS – Virtual Health Library

| Interface: <https://bvsalud.org/en/>  Date of Search: 2024-12-05  Number of hits: 103  Comment: *As the search functionality differs from that of the other databases, the search string was simplified.* |  |
| --- | --- |
| (("resin infiltration" OR "infiltrating resin" OR "resin infiltrant" OR "resin infiltrants" OR "resinous infiltrant" OR "resinous infiltrants")) AND ((incipient OR "early-stage" OR initial OR incipent OR demineralization OR demineralisation OR decalcification OR decalcified * OR "white spot" OR "white-spot" OR "white spots" OR "white-spots")) | |

**Supplementary Material 3.** JBI checklist for Cohort studies (modified); questions in italics are the added ones.

| **Question** | **Yes** | **No** | **Partially** | **Not Applicable** |
| --- | --- | --- | --- | --- |
| *1. Is the sample size sufficient?* |  |  |  |  |
| *2. Was the RI protocol adequately described and appropriate or referred to the manufacturer’s instructions?* |  |  |  |  |
| *3.Was the treatment RI protocol similar for all WSLs?* |  |  |  |  |
| *4. Were the study's inclusion and exclusion criteria clearly described?* |  |  |  |  |
| *5. Was analysis made to determine whether confounding factors affected the outcome?* |  |  |  |  |
| 6. Were the outcomes measured in a valid and reliable way? |  |  |  |  |
| *7. Were the outcome measurements reported performed blinded?* |  |  |  |  |
| *8. Was the outcome measurement done in the same manner at all time points?* |  |  |  |  |
| 9. Was the follow-up time reported and adequate to evaluate outcomes at extended follow-up? |  |  |  |  |
| 10. *Are there any reported differences in patient or WSL characteristics between immediate post-treatment assessments and subsequent follow-up periods?* |  |  |  |  |
| *11. Were patients lost at follow-up a significant concern?* |  |  |  |  |
| 12*.* Were the reasons to loss to follow-up described and explored?  *Answered if yes to question 11* |  |  |  |  |
| 13. Were strategies reported to address incomplete follow-up?  *Answered if yes to question 11* |  |  |  |  |
| 14. Was appropriate statistical analysis used? |  |  |  |  |
| 15. *Was clustering of WSLs/patient taken into account?* |  |  |  |  |
| *16. Are there any conflicts of interest?* |  |  |  |  |

RI, resin infiltration; WSL, white spot lesion.

**Supplementary Material 4.** Reports screened and assessed for eligibility.

**Supplementary Material 4a.** Articles excluded by Title and/or abstract, search date 2025-12-05.

| **Author. (Year). Title. Journal.** |
| --- |
| Bright duties for plate exchangers. (2003). Process Engineering, 84(1), 24.  Effect of resin infiltration on the color and microhardness of bleached white(-)spot lesions in bovine enamel (an in vitro study). (2017). British dental journal, 223(2), 87.  Congress: “Current Practice in Croatian Pediatric Dentistry - 2022”. (2023). Acta Stomatologica Croatica, 57(1).  SPEAK 2024 International Dental Conference. (2024). Journal of Clinical and Diagnostic Research, 18.  Abang Ibrahim, et al. (2024). Resin infiltration – a narrative review of properties. Journal of Health and Translational Medicine, 2024, 153-161.  Abbas, et al. (2018). Treatment of various degrees of white spot lesions using resin infiltration—in vitro study. Progress in Orthodontics, 19(1).  Abdelaziz, et al. (2018). Non-invasive proximal adhesive restoration (NIPAR) compared to resin infiltration for treating initial proximal carious lesions. American Journal of Dentistry, 31(5), 255-260.  Abdelaziz, et al. (2016). Comparing different enamel pretreatment options for resin-infiltration of natural non-cavitated carious lesions. American Journal of Dentistry, 29(1), 3-9.  Abdullah, et al. (2016). Minimally invasive treatment of white spot lesions - a systematic review. Oral Health and Preventive Dentistry, 14(3), 197-205.  Agee, et al. (2015). Water distribution in dentin matrices: Bound vs. unbound water. Dental Materials, 31(3), 205-216.  Ahmed, et al. (2024). Physio-Mechanic and Microscopic Analyses of Bioactive Glass-Based Resin Infiltrants. Microscopy Research and Technique.  Ahmed, et al. (2024). Physico-chemical assessments of ion-doped bioactive glasses-based resin infiltrants. Bioinspired, Biomimetic and Nanobiomaterials.  Akimoto, et al. (2001). Remineralization across the resin-dentin interface: In vivo evaluation with nanoindentation measurements, EDS, and SEM. Quintessence International, 32(7), 561-570.  Al Mamoori, et al. (2022). Esthetic Improvements of Various Degrees of White Spot Lesion Using Resin Infiltration and Micro-Abrasion (An <i>In Vitro</i> Study). Journal of Research in Medical and Dental Science, 10(1), 27-33.  Al Tuwirqi, et al. (2019). Comparison of penetration depth and microleakage of resin infiltrant and conventional sealant in pits and fissures of permanent teeth in vitro. Journal of Contemporary Dental Practice, 20(11), 1339-1344.  Alagha, et al. (2021). Comparing impact of two resin infiltration systems on microhardness of demineralized human enamel after exposure to acidic challenge. Open Access Macedonian Journal of Medical Sciences, 9, 92-97.  Alamoudi, et al. (2022). Patient Satisfaction with Resin Infiltration Treatment for Masking Noncavitated White Spot Lesions on Anterior Maxillary Teeth: Two Case Reports. Case Reports in Dentistry, 2022.  Albassam. (2023). Management of post orthodontic extensive root canal calcification and crown white-spot-lesions using resin infiltration technique. Annals of Dental Specialty, 11(4), 52-55.  Al-Blaihed, et al. (2024). White Spot Lesions in Fixed Orthodontics: A Literature Review on Etiology, Prevention, and Treatment. Cureus Journal of Medical Science, 16(7), 14.  Alfaya, et al. (2013). Tratamento de cárie proximal com infiltrante de resina em paciente adolescente. Rev. Assoc. Paul. Cir. Dent, 67(1), 34-37.  Alghawe, et al. (2024). Management of permanent incisors affected by Molar-Incisor-Hypomineralisation (MIH) using resin infiltration: a pilot study. European Archives of Paediatric Dentistry, 25(1), 105-116.  Alhammad, et al. (2020). Knowledge and Perception of Saudi Dental Professionals Regarding the Use of Resin Infiltration in their Clinical Practice; A Survey-Based Study in Riyadh, KSA. Journal of Pharmaceutical Research International, 32(37), 89-95.  Alkhazaleh, et al. (2023). Management of Multifactorial Stained Enamel Defects on Anterior Dentition Using a Combination Treatment of Whitening, Microabrasion, Resin Infiltration and Resin-based Composite Restoration. Operative Dentistry, 48(3), 251-257.  Alkhudhayri, et al. (2024). Resin Infiltration for the Esthetic Improvement of Dental Fluorosis and White Spots: A Case Report. Cureus, 16(10), e72493.  Allabban. (2020). Evaluation of dental enamel color after fixed orthodontic therapy using two types of protectors to prevent white spot lesions (A prospective clinical study). Journal of Baghdad College of Dentistry, 32(2), 17-21.  Allen, et al. (2021). Resin Infiltration Therapy: A micro-invasive treatment approach for white spot lesions. Journal of Dental Hygiene, 95(6), 31-35.  Al-Mamoori, et al. (2022). Effect of Resin Infiltration and Microabrasion on the Microhardness of the Artificial White Spot Lesions (An in Vitro Study). Journal of Baghdad College of Dentistry, 34(1), 44-50.  Almansouri, et al. (2023). Evaluation of Resin Infiltration, Fluoride and the Biomimetic Mineralization of CPP-ACP in Protecting Enamel after Orthodontic Inter-Proximal Enamel Reduction. Biomimetics, 8(1).  Almaz, et al. (2024). Remineralization effect of NaF, NaF with TCP, NaF with CPP-ACP and NaF with CXP varnishes on newly erupted first permanent molars: A randomized controlled trial. International Journal of Dental Hygiene, 22(3), 703-710.  Almuallem, et al. (2018). Molar incisor hypomineralisation (MIH) - an overview. British Dental Journal, 225(7), 601-609.  Almulhim, et al. (2021). Effect of ageing process and brushing on color stability and surface roughness of treated white spot lesions: An in vitro analysis. Clinical, Cosmetic and Investigational Dentistry, 13, 413-419.  Almutairi, et al. (2021). In vitro, influence of in-office dental whitening on color of teeth treated with resin infiltration. Annals of Dental Specialty, 9(4), 6-11.  Alqahtani, et al. (2021). Effect of topical fluoride application and diode laser-irradiation on white spot lesions of human enamel. Saudi Dental Journal, 33(8), 937-943.  Alqahtani, et al. (2022). Colour stability of resin infiltrated white spot lesion after exposure to stain-causing drinks. Saudi Journal of Biological Sciences, 29(2), 1079-1084.  Alrebdi, et al. (2022). Microabrasion plus resin infiltration in masking white spot lesions. European Review for Medical and Pharmacological Sciences, 26(2), 456-461.  Al-Saeed, et al. (2022). Properties, Success, and Applications of Resin Infiltration for Minimal Invasive Restoration: A Scoping Review. Archives of Pharmacy Practice, 13(2), 110-115.  Alsafi, et al. (2023). Microhardness and surface roughness of resin infiltrated bleached enamel surface using atomic force microscopy: An in vitro study. Saudi Dental Journal, 35(6), 692-698.  Altan, et al. (2023). Clinical evaluation of resin infiltration treatment masking effect on hypomineralised enamel surfaces. Bmc Oral Health, 23(1), 9.  Altarabulsi, et al. (2014). Clinical safety, quality and effect of resin infiltration for proximal caries. European Journal of Paediatric Dentistry, 15(1), 39-44.  Altarabulsi, et al. (2013). Clinical applicability of resin infiltration for proximal caries. Quintessence International, 44(2), 97-104.  Alverson, et al. (2021). Esthetic management of white spot lesions by using minimal intervention techniques of bleaching and resin infiltration: A clinical report. Journal of Prosthetic Dentistry, 126(4), 455-458.  Alwafi. (2017). Resin Infiltration May Be Considered as a Color-Masking Treatment Option for Enamel Development Defects and White Spot Lesions. Journal of Evidence-Based Dental Practice, 17(2), 113-115.  Amend, et al. (2024). Influence of different pre-treatments on the resin infiltration depth into enamel of teeth affected by molar-incisor hypomineralization (MIH). Dental Materials, 40(7), 1015-1024.  Ammari. (2015). Infiltração em lesões cariosas proximais em molares decíduos: ensaio clínico randomizado controlado. https://doi.org/doi:  Ammari, et al. (2018). Efficacy of resin infiltration of proximal caries in primary molars: 1-year follow-up of a split-mouth randomized controlled clinical trial. Clin Oral Investig, 22(3), 1355-1362.  Ammari, et al. (2014). Is non-cavitated proximal lesion sealing an effective method for caries control in primary and permanent teeth? A systematic review and meta-analysis. Journal of Dentistry, 42(10), 1217-1227.  Anauate-Netto, et al. (2015). Infiltrant used to prevent incipient occlusal caries: a 3-year follow-up. Journal of dental research, 94.  Andrade Neto, et al. (2016). Novel hydroxyapatite nanorods improve anti-caries efficacy of enamel infiltrants. Dental Materials, 32(6), 784-793.  Andrade, et al. (2020). Clinical evaluation of the immediate masking effect of enamel white spot lesions treated with an infiltrant resin. The international journal of esthetic dentistry, 15(3), 306-316.  Andrade, et al. (2019). Use of Resin Infiltrate for the Treatment of White spot Lesions on Dental Enamel: Case Report. J. health sci. (Londrina), 21(2), https://seer.pgsskroton.com/index.php/JHealthSci/article/view/6448-https://seer.pgsskroton.com/index.php/JHealthSci/article/view/6448.  Anicic, et al. (2020). The influence of resin infiltration pretreatment on orthodontic bonding to demineralized human enamel. Applied Sciences (Switzerland), 10(10).  Araújo, et al. (2015). Influence of staining solution and bleaching on color stability of resin used for caries infiltration. Operative Dentistry, 40(6), E250-E256.  Araújo, et al. (2024). Infiltração resinosa em lesões brancas cariosas e não cariosas: relato de casos. Rev. Ciênc. Plur; 10 (1) 2024, 10(1), 34213-34213.  Aref, et al. (2022). Casein phosphopeptide amorphous calcium phosphate and universal adhesive resin as a complementary approach for management of white spot lesions: an in-vitro study. Progress in Orthodontics, 23(1).  Aref, et al. (2023). Surface topography and spectrophotometric assessment of white spot lesions restored with nano-hydroxyapatite-containing universal adhesive resin: an in-vitro study. BMC Oral Health, 23(1).  Arjomand, et al. (2021). Effect of tea on color stability of enamel lesions treated with resin infiltrant. Frontiers in Dentistry, 18.  Arnold, et al. (2014). Resin infiltration into differentially extended experimental carious lesions. Open Dentistry Journal, 8(1), 251-256.  Arnold, et al. (2012). Light- and electronmicroscopic study of infiltration of resin into initial caries lesions-a new methodological approach. Journal of Microscopy, 245(1), 26-33.  Arnold, et al. (2015). Enamel surface alterations after repeated conditioning with HCl. Head & Face Medicine, 11, 7.  Arnold, et al. (2016). Surface roughness of initial enamel caries lesions in human teeth after resin infiltration. Open Dentistry Journal, 10, 505-515.  Arora, et al. (2019). An In-Vitro evaluation of resin infiltration system and conventional pit and fissure sealant on enamel properties in white spot lesions. Journal of Indian Society of Pedodontics and Preventive Dentistry, 37(2), 133-139.  Arrais, et al. (2002). Morphology and thickness of the diffusion of resin through demineralized or unconditioned dentinal matrix. Pesquisa odontológica brasileira = Brazilian oral research, 16(2), 115-120.  Arslan, et al. (2018). Effects of different resin sealing therapies on nanoleakage within artificial non-cavitated enamel lesions. Dental Materials Journal, 37(6), 981-987.  Arslan, et al. (2015). Effect of resin infiltration on enamel surface properties and Streptococcus mutans adhesion to artificial enamel lesions. Dental Materials Journal, 34(1), 25-30.  Arthur, et al. (2018). Proximal carious lesions infiltration—a 3-year follow-up study of a randomized controlled clinical trial. Clinical Oral Investigations, 22(1), 469-474.  Ashraf. (2022). Effect of Resin Infiltrate Modified with Bioactive Glass on Hardness and Color Stability of White Spot Lesions. Journal of Research in Medical and Dental Science, 10(5), 66-71.  Asiri. (2023). Resin infiltration: case series. Romanian Journal of Stomatology, 69(3), 163-168.  Askar, et al. (2018). Modified resin infiltration of non-, micro- and cavitated proximal caries lesions in vitro. Journal of Dentistry, 74, 56-60.  Asthana, et al. (2023). Efficacy of microabrasion and resin infiltration techniques for masking of fluorotic white spot lesions: A randomized clinical study. Journal of Conservative Dentistry and Endodontics, 26(6), 677-681.  Aswani, et al. (2019). Resin Infiltration of Artificial Enamel Lesions: Evaluation of Penetration Depth, Surface Roughness and Color Stability. International Journal of Clinical Pediatric Dentistry, 12(6), 520-523.  Athayde, et al. (2022). Impact of masking hypomineralization opacities in anterior teeth on the esthetic perception of children and parents: A randomized controlled clinical trial. J Dent, 123, 104168-104168.  Athayde, et al. (2020). Esthetic Management of Incisors with Diffuse and Demarcated Opacities: 24 Month Follow-up Case Report. Operative Dentistry, 45(6), 569-574.  Attal, et al. (2014). White spots on enamel: Treatment protocol by superficial or deep infiltration (part 2). International Orthodontics, 12(1), 1-31.  Attin, et al. (2021). Effect of Caries Infiltrant on Margin Integrity of Composite Fillings Placed Adjacent to Demineralised Primary Enamel. Oral health & preventive dentistry, 19(1), 603-608.  Attin, et al. (2012). Shear bond strength of brackets to demineralize enamel after different pretreatment methods. Angle Orthodontist, 82(1), 56-61.  Auschill, et al. (2015). Resin Infiltration for Aesthetic Improvement of Mild to Moderate Fluorosis: A Six-month Follow-up Case Report. Oral Health & Preventive Dentistry, 13(4), 317-322.  Ayad, et al. (2022). Effect of different surface treatments on the microhardness and colour change of artificial enamel lesions. Australian Dental Journal, 67(3), 230-238.  Aziznezhad, et al. (2017). Comparison of the effect of resin infiltrant, fluoride varnish, and nano-hydroxy apatite paste on surface hardness and streptococcus mutans adhesion to artificial enamel lesions. Electronic Physician [Electronic Resource], 9(3), 3934-3942.  Babu, et al. (2024). Evaluation of Penetration Depth and Caries Progression using Resin Infiltrant in Natural White Spot Lesions of Primary Molars. International Journal of Clinical Pediatric Dentistry, 17(4), 390-394.  Bagher, et al. (2018). Radiographic Effectiveness of Resin Infiltration in Arresting Incipient Proximal Enamel Lesions in Primary Molars. Pediatric dentistry, 40(3), 195-200.  Bagheri, et al. (2020). Color properties of artificial white spot lesions treated by experimental resin infiltrants containing bioactive glass and nano-fluorohydroxyapatite. The international journal of esthetic dentistry, 15(3), 334-343.  Bak, et al. (2014). Color change of white spot lesions after resin infiltration. Color Research and Application, 39(5), 506-510.  Baka, et al. (2016). Effects of remineralization procedures on shear bond strengths of brackets bonded to demineralized enamel surfaces with self-etch systems. Angle Orthodontist, 86(4), 661-667.  Bakdach, et al. (2020). Effectiveness of different adjunctive interventions in the management of orthodontically induced white spot lesions: A systematic review of systematic reviews and meta-analyses. Dental and Medical Problems, 57(3), 305-325.  Bakhshandeh, et al. (2015). Infiltration and sealing versus fluoride treatment of occlusal caries lesions in primary molar teeth. 2-3 years results. International Journal of Paediatric Dentistry, 25(1), 43-50.  Baldion, et al. (2021). Dataset on the effect of flavonoids on the stabilization of the resin-dentin interface. Data in Brief, 35.  Baptista-Sánchez, et al. (2022). Changes in the Color and Brightness of White Spots Associated with Orthodontic Treatment 6 Months after the Application of Infiltrative Resins: Systematic Review and Meta-Analysis. International Journal of Environmental Research and Public Health, 19(15).  Barbosa, et al. (2018). Tratamento de lesão de mancha branca com infiltrante resinoso: relato de caso. ROBRAC, 27(83), 252-256.  Barboza, et al. (2019). Colour Change and Surface Gloss of Unpolished Infiltrated Bovine Enamel Submitted to Toothbrushing, pH Cycling and Artificial Ageing. Journal of Clinical and Diagnostic Research, 13(6), ZC23-ZC26.  Basak, et al. (2024). Evaluation of Etching Times to Determine the Penetration Depth of Resin Infiltration on Artificially Demineralised Teeth. Journal of Clinical and Diagnostic Research, 18, 8-9.  Basir, et al. (2020). Microhardness of early enamel lesions in deciduous teeth treated with an infiltrant resin, fluoride varnish and GC tooth mousse. Medical Science, 24(105), 2852-2860.  Beerens, et al. (2010). Effects of casein phosphopeptide amorphous calcium fluoride phosphate paste on white spot lesions and dental plaque after orthodontic treatment: a 3-month follow-up. European Journal of Oral Sciences, 118(6), 610-617.  Behrouzi, et al. (2020). Effect of Two Methods of Remineralization and Resin Infiltration on Surface Hardness of Artificially Induced Enamel Lesions. Journal of Dentistry, 21(1), 12-17.  Belli, et al. (2011). Wear and morphology of infiltrated white spot lesions. Journal of Dentistry, 39(5), 376-385.  Bertolo, et al. (2021). Electric current effects on bond strength, nanoleakage, degree of conversion and dentinal infiltration of adhesive systems. J Mech Behav Biomed Mater, 119, 104529-104529.  Betancourt, et al. (2019). Resin-dentin bonding interface: Mechanisms of degradation and strategies for stabilization of the hybrid layer. International Journal of Biomaterials, 2019.  Bhandari, et al. (2021). Evaluation of Aloe vera as matrix metalloproteinase inhibitor in human dentin with and without dentin-bonding agent: An in vitro study. Journal of Conservative Dentistry, 24(5), 491-495.  Blanchet, et al. (2023). Microabrasion in the management of enamel discolorations in paediatric dentistry: a systematic review. Journal of Clinical Pediatric Dentistry, 47(1), 17-26.  Bock, et al. (2024). White spot lesions after fixed appliance treatment-Can we expect spontaneous long-term (≥15 years) improvement? European Journal of Orthodontics, 46(1), 12.  Bolat, et al. (2021). Efficacy of ICON infiltration resin treatment for white spot lesions – case report. Romanian Journal of Oral Rehabilitation, 13(3), 163-166.  Borelli Neto, et al. (2024). Does the Transillumination Technique Using a Diagnostic White Tip Influence the Degree of Conversion of the Infiltrant Resin? A Case Report With In Vitro Insights. Journal of Esthetic and Restorative Dentistry.  Borges. (2014). The concept of resin infiltration technique and its multiple applications. J Contemp Dent Pract, 15(3), i-i.  Borges, et al. (2019). Adhesive systems effect over bond strength of resin-infiltrated and de/remineralized enamel. F1000Research, 8.  Borges, et al. (2014). Color stability of resin used for caries infiltration after exposure to different staining solutions. Operative Dentistry, 39(4), 433-440.  Borges, et al. (2017). Is resin infiltration an effective esthetic treatment for enamel development defects and white spot lesions? A systematic review. Journal of Dentistry, 56, 11-18.  Borges, et al. (2011). Update on nonsurgical, ultraconservative approaches to treat effectively non-cavitated caries lesions in permanent teeth. Eur J Dent, 5(2), 229-236.  Bortolotto, et al. (2009). Morphology of the smear layer after the application of simplified self-etch adhesives on enamel and dentin surfaces created with different preparation methods. Clinical Oral Investigations, 13(4), 409-417.  Botti, et al. (1995). The evaluation of acrylic resins for the study of nondecalcified human teeth with the light and electronic microscopes. Minerva stomatologica, 44(4), 145-153.  Bourouni, et al. (2021). Efficacy of resin infiltration to mask post-orthodontic or non-post-orthodontic white spot lesions or fluorosis — a systematic review and meta-analysis. Clinical Oral Investigations, 25(8), 4711-4719.  Bramhecha, et al. (2021). Resin infiltration. International Journal of Dentistry and Oral Science, 8(8), 3770-3774.  Breschi, et al. (2010). Use of a specific MMP-inhibitor (galardin) for preservation of hybrid layer. Dental Materials, 26(6), 571-578.  Brescia, et al. (2022). Management of Enamel Defects with Resin Infiltration Techniques: Two Years Follow Up Retrospective Study. Children-Basel, 9(9), 11.  Brunton, et al. (2013). Treatment of early caries lesions using biomimetic self-assembling peptides - a clinical safety trial. British Dental Journal, 215(4), 6.  Bulanda, et al. (2022). Management of Teeth Affected by Molar Incisor Hypomineralization Using a Resin Infiltration Technique—A Systematic Review. Coatings, 12(7).  Cabalén, et al. (2022). Nonrestorative Caries Treatment: A Systematic Review Update. International Dental Journal, 72(6), 746-764.  Campanella, et al. (2020). Dentinal substrate variability and bonding effectiveness: Sem investigation. Journal of Biological Regulators and Homeostatic Agents, 34(1), 49-54.  Carmo, et al. (2021). What is the best etching timing in a resin infiltrant used on enamel surface after remineralization with CPP-ACP? Annals of Medicine, 53, S68.  Carvalho, et al. (2005). A challenge to the conventional wisdom that simultaneous etching and resin infiltration always occurs in self-etch adhesives. Biomaterials, 26(9), 1035-1042.  Carvalho, et al. (1995). Bonding mechanism of VariGlass to dentin. American journal of dentistry, 8(5), 253-258.  Cazzolla, et al. (2018). Efficacy of 4-year treatment of icon infiltration resin on postorthodontic white spot lesions. BMJ Case Reports, 2018.  Ceci, et al. (2017). Resin infiltrant for non-cavitated caries lesions: Evaluation of color stability. Journal of Clinical and Experimental Dentistry, 9(2), e231-e237.  Çehreli. (2023). Resin Infiltration: Ultraconservative Treatment Options for Carious and Non-carious Enamel Lesions. Current Oral Health Reports, 10(2), 23-27.  Chabuk, et al. (2023). Surface roughness and microhardness of enamel white spot lesions treated with different treatment methods. Heliyon, 9(7).  Chandrapooja, et al. (2019). Filling without drilling - A review on resin infiltration technique for enamel and early cavitated lesion. Indian Journal of Public Health Research and Development, 10(11), 3445-3449.  Chaple Gil, et al. (2017). Resin infiltration as a minimally invasive treatment for incipient dental caries lesions. Revista Cubana de Estomatologia, 54(1), 100-105.  Chay, et al. (2014). The effect of resin infiltration and oxidative pre-treatment on microshear bond strength of resin composite to hypomineralised enamel. International Journal of Paediatric Dentistry, 24(4), 252-267.  Chellapandian, et al. (2020). Comparison and evaluation of caries preventive efficacy of resin infiltrant, casein phosphopeptide amorphous calcium phosphate and nanohydroxyapatite using vickers microhardness tester- an in vitro study. European Journal of Molecular and Clinical Medicine, 7(10), 637-645.  Chellapandian, et al. (2020). Comparative efficacy of resin infiltrant and two remineralizing agents on demineralized enamel: An in vitro study. Journal of Contemporary Dental Practice, 21(7), 792-797.  Chen, et al. (2019). Accelerated aging effects on color, microhardness and microstructure of ICON resin infiltration. European Review for Medical and Pharmacological Sciences, 23(18), 7722-7731.  Cheng. (2022). Resin infiltration may help mask enamel white spot lesions or fluorosis. Journal of Evidence-Based Dental Practice.  Cheng, et al. (2021). Restoration effect and stability of resin infiltration combined with bioactive glass on demineralized tooth enamel. Chinese Journal of Tissue Engineering Research, 25(22), 3522-3526.  Chiaraputt, et al. (2008). Changes in resin-infiltrated dentin stiffness after water storage. Journal of Dental Research, 87(7), 655-660.  Chiba, et al. (2016). The effects of ethanol on the size-exclusion characteristics of type i dentin collagen to adhesive resin monomers. Acta Biomaterialia, 33, 235-241.  Chindane, et al. (2022). Effect of CPP-ACPF, resin infiltration, and colloidal silica infiltration on surface microhardness of artificial white spot lesions in primary teeth: An in vitro study. Dental Research Journal, 19, 52.  Çiloğlu, et al. (2024). Visualization of etching cycles efficacy at the resin infiltration into artificial enamel caries: in-vitro study on bovine teeth. Journal of Clinical Pediatric Dentistry, 48(1), 191-197.  Clarke-Martin, et al. (1989). Scanning microscopy of resin infiltration patterns in acid-etched incisor enamel. Clinical preventive dentistry, 11(3), 3-5.  Cocco, et al. (2016). Treatment of fluorosis spots using a resin infiltration technique: 14-Month follow-up. Operative Dentistry, 41(4), 357-362.  Cocco, et al. (2017). Enamel microabrasion associated with resin infiltration technique: A clinical report. Brazilian Dental Science, 20(2), 139-145.  Cochrane, et al. (2012). An X-ray Microtomographic Study of Natural White-spot Enamel Lesions. Journal of Dental Research, 91(2), 185-191.  Cohen-Carneiro, et al. (2014). Color stability of carious incipient lesions located in enamel and treated with resin infiltration or remineralization. International Journal of Paediatric Dentistry, 24(4), 277-285.  Ctri. (2019). Effectiveness Of Recent Remineralizing Agents On Defects Of Permanent Incisors. https://trialsearch.who.int/Trial2.aspx?TrialID=CTRI/2019/01/017345.  Ctri. (2022). Comparision of two different techniques for removing flourotic white spot lesions. https://trialsearch.who.int/Trial2.aspx?TrialID=CTRI/2022/09/046029.  Ctri. (2024). A clinical trial to study the effects of resin modified glass ionomer varnish and resin infiltrant on initial carious lesions in primary teeth. https://trialsearch.who.int/Trial2.aspx?TrialID=CTRI/2024/03/064408.  Cuppini, et al. (2021). Ionic liquid-loaded microcapsules doped into dental resin infiltrants. Bioact Mater, 6(9), 2667-2675.  Da Cunha, et al. (2023). Treatment protocol of dental bleaching and resin infiltration for white spot lesions. International Journal of Esthetic Dentistry, 18(2), 200-206.  da Freiria, et al. (2022). Nano-hydroxyapatite-induced remineralization of artificial white spot lesions after bleaching treatment with 10% carbamide peroxide. Journal of Esthetic and Restorative Dentistry, 34(8), 1290-1299.  Dai, et al. (2022). Novel nanostructured resin infiltrant containing calcium phosphate nanoparticles to prevent enamel white spot lesions. Journal of the Mechanical Behavior of Biomedical Materials, 126.  Damian, et al. (2022). Impact of Dentistry Materials on Chemical Remineralisation/Infiltration versus Salivary Remineralisation of Enamel—In Vitro Study. Materials, 15(20).  Daneshkazemi, et al. (2021). Shear bond strength of orthodontic brackets on intact and demineralized enamel after application of resin infiltrant, fluoride varnish and casein phosphopeptide-amorphous calcium phosphate remineralizing agents: in-vitro study. International Orthodontics, 19(2), 259-268.  Daood, et al. (2021). PLGA nanoparticles loaded with quaternary ammonium silane and riboflavin for potential applications in adhesive dentistry. International Journal of Adhesion and Adhesives, 105.  Daood, et al. (2015). In vitro analysis of riboflavin-modified, experimental, two-step etch-and-rinse dentin adhesive: Fourier transform infrared spectroscopy and micro-Raman studies. International Journal of Oral Science, 7(2), 110-124.  Daood, et al. (2013). Effect of chitosan/riboflavin modification on resin/dentin interface: Spectroscopic and microscopic investigations. Journal of Biomedical Materials Research - Part A, 101(7), 1846-1856.  de Albuquerque, et al. (2022). Resin infiltrant protects deproteinized dentin against erosive and abrasive wear. Restor Dent Endod, 47(3), e29-e29.  De Alencar, et al. (2015). Resin infiltration of early carious lesion in early childhood: A case report. General Dentistry, 63(5), 48-51.  de Almeida, et al. (2023). Non-Invasive Methods and the Use of Infiltrating Resins for the Control of Caries Progression in Deciduous Teeth: A Systematic Review and Meta-Analysis. Pesquisa Brasileira em Odontopediatria e Clinica Integrada, 23.  de Carvalho, et al. (2019). Influence of infiltrant application time on the reduction of opaqueness of proximal enamel caries. Indian J Dent Res, 30(1), 52-56.  de Cerqueira, et al. (2023). Roughness and Microhardness of Demineralized Enamel Treated with Resinous Infiltrants and Subjected to an Acid Challenge: An in vitro Study. Open Dentistry Journal, 17(1).  de Holanda Ferreira, et al. (2023). Organic volume and permeability variations in the surface layer of artificial and natural enamel carious lesions. Archives of Oral Biology, 148.  de Lacerda, et al. (2016). Adhesive systems as an alternative material for color masking of white spot lesions: Do they work? Journal of Adhesive Dentistry, 18(1), 43-50.  De Lacerda, et al. (2016). Influence of de/remineralization of enamel on the tensile bond strength of etch-and-rinse and self-etching adhesives. American Journal of Dentistry, 29(5), 289-293.  de Oliveira Correia, et al. (2020). Color masking prediction of posterior white spot lesions by resin infiltration in vitro. Journal of Dentistry, 95.  de Oliveira, et al. (2022). Enamel Developmental Defect Masking on Central Incisor with Infiltrant Resin. Operative dentistry, 47(5), 476-480.  de Oliveira, et al. (2010). Micromorphology of resin-dentin interfaces using one-bottle etch&rinse and self-etching adhesive systems on laser-treated dentin surfaces: a confocal laser scanning microscope analysis. Lasers Surg Med, 42(7), 662-670.  de Sousa, et al. (2013). Natural Enamel Caries: A Comparative Histological Study on Biochemical Volumes. Caries Research, 47(3), 183-192.  Decha, et al. (2019). Synthesis and characterization of new hydrolytic-resistant dental resin adhesive monomer HMTAF. Designed Monomers and Polymers, 22(1), 106-113.  Demirbuga, et al. (2019). Microshear bond strength of universal adhesives for use with artificially demineralized enamel, with and without pre-etching. Journal of Adhesion Science and Technology, 33(4), 346-354.  Desai, et al. (2021). Minimally Invasive Therapies for the Management of Dental Caries-A Literature Review. Dentistry Journal, 9(12), 27.  Dhillon, et al. (2020). Comparative evaluation of microhardness and enamel solubility of treated surface enamel with resin infiltrant, fluoride varnish, and casein phosphopeptide-amorphous calcium phosphate: An in vitro study. International Journal of Clinical Pediatric Dentistry, 13, S14-S25.  Di Giovanni, et al. (2018). Interventions for dental fluorosis: A systematic review. Journal of Esthetic and Restorative Dentistry, 30(6), 502-508.  Di Hipólito, et al. (2005). SEM evaluation of contemporary self-etching primers applied to ground and unground enamel. J Adhes Dent, 7(3), 203-211.  Dilber, et al. (2015). Effects of Different Demineralization-Inhibiting Methods on the Shear Bond Strength of Glass-Ceramics. Journal of Prosthodontics, 24(5), 407-413.  Diniz, et al. (2021). The Evaluation of Different Treatments of Incipient Caries Lesions: An in Situ Study of Progression Using Fluorescence-based Methods. Operative Dentistry, 46(1), 87-99.  Dogra, et al. (2020). Spectrophotometric evaluation of color change in tooth enamel defects using resin infiltrate: An in vivo study. International Journal of Clinical Pediatric Dentistry, 13(2), 150-154.  Dogra, et al. (2020). Avminimally invasive technique of masking nonpitted fluorosis on young permanent incisors: A clinical trial. World Journal of Dentistry, 11(1), 41-46.  Doğu Kaya, et al. (2024). Effect of the use of remineralization agents before resin infiltration on the treatment of initial enamel lesions: an in-vitro study. BMC Oral Health, 24(1).  Doméjean, et al. (2015). Resin Infiltration of Non-Cavitated Caries Lesions: A Systematic Review. Medical Principles and Practice, 24(3), 216-221.  Dorri, et al. (2015). Micro-invasive interventions for managing proximal dental decay in primary and permanent teeth. Cochrane Database of Systematic Reviews, 2015(11).  Dziaruddin, et al. (2022). Resin Infiltration of Non-Cavitated Enamel Lesions in Paediatric Dentistry: A Narrative Review. Children, 9(12).  Eden. (2016). Evidence-based caries prevention. Springer International Publishing. https://doi.org/doi:10.1007/978-3-319-40034-1  Edunoori, et al. (2022). Comparison of the efficacy of Icon resin infiltration and Clinpro XT varnish on remineralization of white spot lesions: An in-vitro study. Journal of Orthodontic Science, 11(1), 12.  Eick, et al. (1996). Quantitative analysis of the dentin adhesive interface by Auger spectroscopy (Vol. 75). Intern. and American Associations for Dental Research. https://doi.org/doi:10.1177/00220345960750040501  Eissa, et al. (2022). Effect of sodium fluoride plus tricalcium phosphate with and without CO2 laser on remineralization of white spot lesions. Heliyon, 8(10), 8.  Ekizer, et al. (2012). Effects of demineralizaton-inhibition procedures on the bond strength of brackets bonded to demineralized enamel surface. Korean Journal of Orthodontics, 42(1), 17-22.  El Meligy, et al. (2021). Effect of resin infiltration application on early proximal caries lesions in vitro. Journal of Dental Sciences, 16(1), 296-303.  El Tanany, et al. (2023). Effects of Icon Treatment and Bleaching on the Color of Enamel with Induced White-Spot Lesio ns. Journal of Clinical Orthodontics, 57(5), 298-305.  Elembaby, et al. (2022). The Impact of Nano-Hydroxyapatite Resin Infiltrant on Enamel Remineralization: An in Vitro Study. International Journal of Periodontics and Restorative Dentistry, 42(2), E43-E50.  Elgamily, et al. (2024). Influence of pre-treatment with diode laser and Nano silica coating crosslinking matrix metalloproteinase inhibitor on the stabilization of resin-dentine interfaces. Lasers in Dental Science, 8(1).  Elhiny, et al. (2016). The influence of resin infiltration system on sound enamel microhardness and shear-bond strength of orthodontic bands: An in-vitro study. Der Pharma Chemica, 8(18), 100-106.  Eliades, et al. (2001). Heterogeneous distribution of single-bottle adhesive monomers in the resin-dentin interdiffusion zone. Dental Materials, 17(4), 277-283.  Enan, et al. (2019). Resistance of resin-infiltrated enamel to surface changes in response to acidic challenge. Journal of Esthetic and Restorative Dentistry, 31(4), 353-358.  Esmaeilnia Shirvani, et al. (2022). Laboratory Evaluation of Color Change and Surface Roughness of White Spot Lesions Treated with Resin Infiltration and Fluoride Therapy. Journal of Babol University of Medical Sciences, 24(1), 338-346.  Espigares, et al. (2018). Enamel margins resealing by low-viscosity resin infiltration. Dental Materials Journal, 37(2), 350-357.  Esteves-Oliveira, et al. (2022). Randomized in situ evaluation of surface polishing protocols on the caries-protective effect of resin Infiltrant. Scientific Reports, 12(1).  Faghihian, et al. (2019). Efficacy of the Resin Infiltration Technique in Preventing Initial Caries Progression: A Systematic Review and Meta-Analysis. Pediatric dentistry, 41(2), 88-94.  Farias, et al. (2022). Microinvasive esthetic approach for deep enamel white spot lesion. Dental Research Journal, 19, 29.  Farina, et al. (2020). Removal of water binding proteins from dentin increases the adhesion strength of low-hydrophilicity dental resins. Dental Materials, 36(10), e302-e308.  Fattah, et al. (2021). Nanotechnology in tooth-tissue regenerative therapy. Elsevier. https://doi.org/doi:10.1016/B978-0-323-90282-3.00011-2  Favero, et al. (2024). Different effects of whitening treatment on natural teeth and teeth treated with Icon resin: an experimental study in vitro. European Journal of Paediatric Dentistry, 25(2), 132-136.  Fawzy, et al. (2019). Potential of high-intensity focused ultrasound in resin-dentine bonding. Dental Materials, 35(7), 979-989.  Fawzy, et al. (2009). Probing nano-scale adhesion force between AFM and acid demineralized intertubular dentin: Moist versus dry dentin. Journal of Dentistry, 37(12), 963-969.  Feng, et al. (2013). Effect of infiltration resin on the color masking of labial enamel white spot lesions. Hua xi kou qiang yi xue za zhi = Huaxi kouqiang yixue zazhi = West China journal of stomatology, 31(6), 597-599.  Fernández-Ferrer, et al. (2018). Enamel remineralization therapies for treating postorthodontic white-spot lesions A systematic review. Journal of the American Dental Association, 149(9), 778-+.  Ferreira, et al. (2018). High Amount of Organic Matter during Caries Formation Reduces Remineralization and Resin Infiltration of Enamel Caries. Caries Research, 52(6), 580-587.  Ferreira, et al. (2019). The Use of Resinous Infiltrants for the Management of Incipient Carious Lesions: a Literature Review. J. health sci. (Londrina), 21(4), https://seer.pgsskroton.com/index.php/JHealthSci/article/view/6808-https://seer.pgsskroton.com/index.php/JHealthSci/article/view/6808.  Fischer, et al. (2021). Assessment of the potential ability to penetrate into the hard tissues of the root of an experimental preparation with the characteristics of a dental infiltrate, enriched with an antimicrobial component—preliminary study. Materials, 14(19).  Florenzano, et al. (2023). Fact-finding investigation on the approach to post-orthodontic white enamel lesions. Dental Cadmos, 91(9), 752-761.  Flor-Ribeiro, et al. (2019). Effect of iodonium salt and chitosan on the physical and antibacterial properties of experimental infiltrants. Brazilian Oral Research, 33.  Freitas, et al. (2018). In vitro effect of a resin infiltrant on different artificial caries-like enamel lesions. Archives of Oral Biology, 95, 118-124.  Freitas. (2015). Efeito de um infiltrante resinoso no tratamento de lesões de mancha branca: análise in vitro e in situ. https://doi.org/doi:  Fuentes, et al. (2004). Tensile strength and microhardness of treated human dentin. Dental Materials, 20(6), 522-529.  Furuse, et al. (2020). Color evaluation of white spot lesions treated with resin infiltration after water or grape juice storage. Braz. j. oral sci, 19, e201674-e201674.  g. (2020). Aesthetic treatment of stains on anterior teeth caused by hypomineralization. https://trialsearch.who.int/Trial2.aspx?TrialID=RBR-92g625.  Gaglianone, et al. (2020). Can composition and preheating improve infiltrant characteristics and penetrability in demineralized enamel? Brazilian Oral Research, 34.  Gan, et al. (2015). Enhanced transport of materials into enamel nanopores via electrokinetic flow. Journal of Dental Research, 94(4), 615-621.  García, et al. (2013). Tratamiento estético de manchas blancas de esmalte posortodoncia: asociación de blanqueamiento dental e infiltración resinosa. Rev. Asoc. Odontol. Argent, 101(1), 17-20.  Garcia, et al. (2005). Effects of solvents on the early stage stiffening rate of demineralized dentin matrix. Journal of Dentistry, 33(5), 371-377.  Garcia, et al. (2021). Magnetic motion of superparamagnetic iron oxide nanoparticles- loaded dental adhesives: physicochemical/biological properties, and dentin bonding performance studied through the tooth pulpal pressure model. Acta Biomater, 134, 337-347.  Garg, et al. (2020). Color Masking White Fluorotic Spots by Resin Infiltration and Its Quantitation by Computerized Photographic Analysis: A 12-month Follow-up Study. Operative Dentistry, 45(1), 1-9.  Gelani, et al. (2014). In vitro progression of artificial white spot lesions sealed with an infiltrant resin. Operative Dentistry, 39(5), 481-488.  Gencer, et al. (2019). A comparison of the effectiveness of resin infiltration and microabrasion treatments applied to developmental enamel defects in color masking. Dental Materials Journal, 38(2), 295-302.  Gevert, et al. (2022). How is the quality of the available evidence on molar-incisor hypomineralization treatment? An overview of systematic reviews. Clin Oral Investig, 26(10), 5989-6002.  Gevkaliuk, et al. (2024). Morphological evaluation of the effectiveness of the "Icon" resin infiltration method in acute and chronic superficial dental caries. Regulatory Mechanisms in Biosystems, 15(1), 142-147.  Gholamrezayi, et al. (2023). Systematic review of preventive and treatment measures regarding orthodontically induced white spot lesions. Dental and Medical Problems, 60(3), 527-535.  Giannetti, et al. (2018). Deep infiltration for the treatment of hypomineralized enamel lesions in a patient with molar incisor hypomineralization: a clinical case. Journal of Biological Regulators and Homeostatic Agents, 32(3), 751-754.  Gileva, et al. (2024). Integrated approach to the study of biodegradation of composite materials for the restoration of hard dental tissues. Permskij Medicinskij Zurnal, 41(1), 73-80.  Gileva, et al. (2021). Changes in Mechanical and Microstructural Characteristics of Demineralized Tooth Enamel after Conventional and Modified Infiltration Techniques (Vol. 1945). IOP Publishing Ltd. https://doi.org/doi:10.1088/1742-6596/1945/1/012007  Godenzi, et al. (2023). Remineralizing potential of the biomimetic P11-4 self-assembling peptide on noncavitated caries lesions A retrospective cohort study evaluating semistandardized before-and-after radiographs. Journal of the American Dental Association, 154(10), 885-+.  Goldschmidt, et al. (2017). An evaluation of the clinical and histological effects of high dose radiosurgery on the rat dorsal root ganglion. Clinical Neurosurgery, 64, 266-267.  Golz, et al. (2016). In vitro biocompatibility of ICON(R) and TEGDMA on human dental pulp stem cells. Dental Materials, 32(8), 1052-1064.  Gomes, et al. (2016). At-home tray whitening and resin infiltration after acid etching with HCl. Springer International Publishing. https://doi.org/doi:10.1007/978-3-319-38849-6_13  Gomes, et al. (2023). Clinical Case of At-Home Bleaching and Resin Infiltration. Springer International Publishing. https://doi.org/doi:10.1007/978-3-031-38244-4_12  Gray, et al. (2002). Infiltration of resin into white spot caries-like lesions of enamel: an in vitro study. The European journal of prosthodontics and restorative dentistry, 10(1), 27-32.  Greenwall. (2017). Tooth whitening, the microabrasion technique, and white spot eradication. CRC Press. https://doi.org/doi:10.1201/9781315365503  Grégoire, et al. (2013). Depletion of water molecules during ethanol wet-bonding with etch and rinse dental adhesives. Materials Science and Engineering C, 33(1), 21-27.  Gu, et al. (2010). Changes in stiffness of resin-infiltrated demineralized dentin after remineralization by a bottom-up biomimetic approach. Acta Biomaterialia, 6(4), 1453-1461.  Gu, et al. (2010). A chemical phosphorylation-inspired design for Type i collagen biomimetic remineralization. Dental Materials, 26(11), 1077-1089.  Gugnani, et al. (2014). Esthetic improvement of white spot lesions and non-pitted fluorosis using resin infiltration technique: Series of four clinical cases. Journal of Indian Society of Pedodontics and Preventive Dentistry, 32(2), 176-180.  Gugnani, et al. (2017). Comparative evaluation of esthetic changes in nonpitted fluorosis stains when treated with resin infiltration, in-office bleaching, and combination therapies. Journal of Esthetic and Restorative Dentistry, 29(5), 317-324.  Gugnani, et al. (2012). Caries infiltration of noncavitated white spot lesions: A novel approach for immediate esthetic improvement. Contemporary Clinical Dentistry, 3(6), S199-S202.  Guimaraes, et al. (2009). A shorter fixation protocol for transmission electron microscopy: an alternative to spend less time. Ultrastruct Pathol, 33(4), 169-174.  Guimarães, et al. (2022). Effects of Alternative Solvents in Experimental Enamel Infiltrants on Bond Strength and Selected Properties. Biomed Res Int, 2022, 4293975-4293975.  Gulec, et al. (2019). Assessment of the resin infiltration and CPP-ACP applications before orthodontic brackets bonding. Dental Materials Journal, 38(5), 854-860.  Guo, et al. (2014). Effect of Varnish XT and ICON resin infiltration treatment on the microhardness of white spot lesions. Chinese Journal of Tissue Engineering Research, 18(47), 7638-7642.  Guo, et al. (2019). Polymer conjugation optimizes EDTA as a calcium-chelating agent that exclusively removes extrafibrillar minerals from mineralized collagen. Acta Biomaterialia, 90, 424-440.  Gurdogan, et al. (2017). Evaluation of Surface Roughness Characteristics Using Atomic Force Microscopy and Inspection of Microhardness Following Resin Infiltration with Icon®. Journal of Esthetic and Restorative Dentistry, 29(3), 201-208.  Gutiérrez-Camacho, et al. (2022). In-Vitro Silanization of Dental Enamel to Prevent Demineralization. Odovtos - International Journal of Dental Sciences, 24(1), 76-86.  Gölz, et al. (2016). In vitro biocompatibility of ICON® and TEGDMA on human dental pulp stem cells. Dental Materials, 32(8), 1052-1064.  Halcomb, et al. (2020). Pediatric Dentists’ Educational Experiences, Attitudes, and Professional Behavior Concerning Resin Infiltration: Implications for Dental Education. Journal of Dental Education, 84(3), 290-300.  Hallgren, et al. (2016). Color Properties of Demineralized Enamel Surfaces Treated with a Resin Infiltration System. Journal of Esthetic and Restorative Dentistry, 28(5), 339-346.  Hammad, et al. (2012). Effect of resin infiltration on white spot lesions after debonding orthodontic brackets. American Journal of Dentistry, 25(1), 3-8.  Hammad, et al. (2020). Evaluation of color changes of white spot lesions treated with three different treatment approaches: An in-vitro study. Dental Press Journal of Orthodontics, 25(1), 27.e21-27.e27.  Hammad, et al. (2013). In vivo effects of two acidic soft drinks on shear bond strength of metal orthodontic brackets with and without resin infiltration treatment. Angle Orthodontist, 83(4), 648-652.  Hammad, et al. (2016). Efficacy of a new sealant to prevent white spot lesions during fixed orthodontic treatment. Journal of Orofacial Orthopedics-Fortschritte Der Kieferorthopadie, 77(6), 439-445.  Han, et al. (2000). WDX Study of Resin-dentin Interface on Wet vs. Dry Dentin. Dental Materials Journal, 19(3), 317-325.  Hanashiro. (2012). Avaliação in vitro das superfícies vestibulares desmineralizadas e restauradas com resina infiltrante. https://doi.org/doi:  Hannig, et al. (2001). Composite-to-dentin bond strength, micromorphology of the bonded dentin interface and marginal adaptation of class II composite resin restorations using self-etching primers. Operative Dentistry, 26(2), 157-165.  Harhash, et al. (2017). Radiographic evaluation of non-operative repair depths for various preventive approaches. Research Journal of Pharmaceutical, Biological and Chemical Sciences, 8(3), 290-299.  Hashemian, et al. (2021). A modified TEGDMA-based resin infiltrant using polyurethane acrylate oligomer and remineralising nano-fillers with improved physical properties and remineralisation potential. Journal of Dentistry, 113.  Hashimoto, et al. (2006). The effects of common errors on sealing ability of total-etch adhesives. Dental Materials, 22(6), 560-568.  Hedayet, et al. (2021). Colour of White Spot Lesions after Non-Invasive Treatment Modalities: An in-vitro study. Ain Shams Dental Journal (Egypt), 21(1), 41-48.  Hernández, et al. (2014). Guided Tissue Remineralisation of Partially Demineralised Human Dentin. Journal of Esthetic and Restorative Dentistry, 26(2), 141-142.  Hilgert, et al. (2016). Resin infiltration: A microinvasive treatment for carious and hypomineralised enamel lesions. Springer International Publishing. https://doi.org/doi:10.1007/978-3-319-40034-1_8  Hoan, et al. (2024). Effectiveness of resin infiltration in the management of anterior teeth affected by molar incisor hypomineralisation (MIH): A systematic review and meta-analysis. Journal of Dentistry, 149, 13.  Horuztepe, et al. (2017). Effect of resin infiltration on the color and microhardness of bleached white-spot lesions in bovine enamel (an in vitro study). Journal of Esthetic and Restorative Dentistry, 29(5), 378-385.  Horuztepe, et al. (2021). Comparison of Resin Infiltration Technique with Conventional Preventive Applications on Occlusal Fissures: EDS and SEM Analyses. Acta Stomatologica Croatica, 54(4), 382-391.  Huang, et al. (2022). Resin Infiltration May Be a Feasible Option to Esthetically Mask Enamel White Spot Lesions. The Journal of Evidencebased Dental Practice, 22(2), 101715.  Huang, et al. (2022). Novel dental resin infiltrant containing smart monomer dodecylmethylaminoethyl methacrylate. Frontiers in Cellular and Infection Microbiology, 12.  Hussein, et al. (2019). Effect of staining and brushing on the surface roughness of white spot lesions treated with resin infiltration technique. Indian Journal of Public Health Research and Development, 10(10), 2734-2739.  Höchli, et al. (2017). Interventions for orthodontically induced white spot lesions: a systematic review and meta-analysis. European journal of orthodontics, 39(2), 122-133.  Iatarola. (2024). Clareamento como complementação à infiltração resinosa na dissimulação de lesões de mancha branca em esmalte bovino escurecido. https://doi.org/doi:  Ibrahim, et al. (2023). Repeated Etching Cycles of Resin Infiltration up to Nine Cycles on Demineralized Enamel: Surface Roughness and Esthetic Outcomes—In Vitro Study. Children, 10(7).  Ibrahim, et al. (2024). Resin infiltration for the management of enamel opacity – a case series and literature review. Journal of Health and Translational Medicine, 27(1), 134-140.  Ibrahim, et al. (2023). Esthetic effects and color stability of resin infiltration on demineralized enamel lesions: A systematic review. Journal of International Society of Preventive and Community Dentistry, 13(4), 284-286.  Inagaki, et al. (2016). Effect of monomer blend and chlorhexidine-adding on physical, mechanical and biological properties of experimental infiltrants. Dental Materials, 32(12), e307-e313.  Inagaki, et al. (2016). Evaluation of sorption/solubility, softening, flexural strength and elastic modulus of experimental resin blends with chlorhexidine. Journal of Dentistry, 49, 40-45.  Insee, et al. (2024). Shear Bond Strength of Orthodontic Brackets to Resin-Infiltrated Enamel. European Journal of General Dentistry.  Ionta, et al. (2016). Penetration of Resin-Based Materials Into Initial Erosion Lesion: A Confocal Microscopic Study. Microscopy Research and Technique, 79(2), 72-80.  Irinoda, et al. (2000). Effect of sealant viscosity on the penetration of resin into etched human enamel. Operative dentistry, 25(4), 274-282.  Ismail, et al. (2015). The International Caries Classification and Management System (ICCMS ™) an example of a Caries Management Pathway. Bmc Oral Health, 15, 13.  Isrctn. (2022). Treatment of permanent front teeth affected by hypomineralisation (white and yellow spots). https://trialsearch.who.int/Trial2.aspx?TrialID=ISRCTN88218639.  Ivanović, et al. (2019). Microinvasive therapy of hipomineralized enamel by application of infiltration resin. Acta Stomatologica Croatica, 53(3), 282.  Iwasa, et al. (2011). pH Changes upon Mixing of Single-step Self-etching Adhesives with Powdered Dentin. Journal of Adhesive Dentistry, 13(3), 207-212.  Iziumov, et al. (2017). Biomechanical modelling of the caries-infiltration procedure of the photopolymer into the dental enamel. Russian Journal of Biomechanics, 21(4), 297-309.  Jacob, et al. (2023). Effect of bleaching on color and surface topography of teeth with enamel caries treated with resin infiltration (ICON®) and remineralization (casein phosphopeptide-amorphous calcium phosphate). Journal of Conservative Dentistry and Endodontics, 26(4), 377-382.  Jacques, et al. (2005). Effect of dentin conditioners on the microtensile bond strength of a conventional and a self-etching primer adhesive system. Dental Materials, 21(2), 103-109.  Jansen, et al. (2021). Do bleaching gels affect the stability of the masking and caries-arresting effects of caries infiltration-in vitro. Clinical Oral Investigations, 25(6), 4011-4021.  Jauković, et al. (2023). Resin infiltration as an aesthetic solution for a patient with enamel hypomineralization caused by dental fluorosis. Acta Stomatologica Croatica, 57(1), 88.  Jeronymo, et al. (2012). Effect of caries infiltration technique and fluoride therapy on the bond strength of the demineralized enamel. Braz. dent. sci, 15(3), 43-49.  Jia, et al. (2012). Effect of caries infiltrant application on shear bond strength of different adhesive systems to sound and demineralized enamel. Journal of Adhesive Dentistry, 14(6), 569-574.  Jiang, et al. (2023). The effect of calcium phosphate ion clusters in enhancing enamel conditions versus Duraphat and Icon. Australian Endodontic Journal, 49, 46-57.  Johannessen, et al. (1981). X-ray microanalytical studies of initial mineralization in induced heterotopic bone formation in Guinea pigs. Acta Odontologica Scandinavica, 39(4), 217-226.  Jorge. (2017). Eficácia da técnica de infiltração em lesões cariosas proximais de molares decíduos: ensaio clínico controlado randomizado com 24 meses de acompanhamento. https://doi.org/doi:  Jorge, et al. (2019). Randomized controlled clinical trial of resin infiltration in primary molars: 2 years follow-up. J Dent, 90, 103184-103184.  Jumanca, et al. (2018). Comparative study of acid etching on dental enamel. Revista de Chimie, 69(10), 2913-2915.  Kabeel, et al. (2024). The Effect of Resin Infiltration Technique on the Calculated Color Change of Demineralized Lesions of Different Severities. Operative dentistry, 49(4), 443-454.  Kalender, et al. (2024). Surface conditioning of artificial caries lesions with Er, Cr:YSGG laser before resin infiltration: an in vitro study. Lasers in Dental Science, 8(1).  Kantovitz, et al. (2010). Review of the effects of infiltrants and sealers on non-cavitated enamel lesions. Oral Health and Preventive Dentistry, 8(3), 295-305.  Karimi, et al. (2023). Polychromatic Composite and Resin Infiltration Restorations in the Esthetic Zone: A Five-year Clinical Report. Operative dentistry, 48(3), 245-250.  Kaviya, et al. (2020). Survey on management of white spot lesions. Indian Journal of Forensic Medicine and Toxicology, 14(4), 5054-5062.  Kerälä, et al. (2024). Dental Hygienists' Readiness to Perform Resin Infiltrations: A Qualitative Study From Finland. International Journal of Dental Hygiene, 10.  Khalid, et al. (2019). Differences in enamel surface roughness changes after debonding using resin infiltration system and nano-filled resin coating. Journal of International Dental and Medical Research, 12(1), 95-100.  Khan, et al. (2023). Application of laser on enamel surface with three types of bioactive glasses-based resin infiltrants: An in vitro study. Journal of the Mechanical Behavior of Biomedical Materials, 141.  Khan, et al. (2020). Technique Tips Resin Infiltration Technique on Enamel Opacities and White Spot Lesions (WSLs). Dental Update, 47(6), 536-537.  Khanna, et al. (2020). Quantitative evaluation of masking effect of resin infiltration on developmental defects of enamel. Quintessence International, 51(6), 448-455.  Khoroushi, et al. (2017). Prevention and treatment of white spot lesions in orthodontic patients. Contemporary Clinical Dentistry, 8(1), 11-19.  Kielbassa, et al. (2020). Ex vivo investigation on internal tunnel approach/internal resin infiltration and external nanosilver-modified resin infiltration of proximal caries exceeding into dentin. PLoS ONE, 15(1).  Kielbassa, et al. (2009). Closing the gap between oral hygiene and minimally invasive dentistry: A review on the resin infiltration technique of incipient (proximal) enamel lesions. Quintessence International, 40(8), 663-681.  Kielbassa, et al. (2017). Resin infiltration of deproteinised natural occlusal subsurface lesions improves initial quality of fissure sealing. International Journal of Oral Science, 9(2), 117-124.  Kielbassa, et al. (2017). External and internal resin infiltration of natural proximal subsurface caries lesions: A valuable enhancement of the internal tunnel restoration. Quintessence International, 48(5), 357-368.  Kim, et al. (2010). Functional biomimetic analogs help remineralize apatite-depleted demineralized resin-infiltrated dentin via a bottom-up approach. Acta Biomaterialia, 6(7), 2740-2750.  Kim, et al. (2019). High-Frequency Ultrasound Imaging for Examination of Early Dental Caries. Journal of Dental Research, 98(3), 363-367.  Kim, et al. (2011). The evaluation of resin infiltration for masking labial enamel white spot lesions. International Journal of Paediatric Dentistry, 21(4), 241-248.  Klaisiri, et al. (2020). Microleakage of resin infiltration in artificial white-spot lesions. Journal of Oral Science, 62(4), 427-429.  Klaisiri, et al. (2023). Methylene Blue Penetration of Resin Infiltration and Resin Sealant in Artificial White-Spot Lesions. European Journal of Dentistry, 17(3), 828-833.  Knösel, et al. (2019). Comparison of potential long-term costs for preventive dentistry treatment of post-orthodontic labial versus lingual enamel cavitations and esthetically relevant white-spot lesions: a simulation study with different scenarios. Head & Face Medicine, 15(1), 10.  Knösel, et al. (2017). Infiltration of white-spot-lesions and developmental enamel defects. Rev. clín. periodoncia implantol. rehabil. oral (Impr.), 10(2), 101-106.  Kobbe, et al. (2019). Evaluation of the value of re-wetting prior to resin infiltration of post-orthodontic caries lesions. Journal of Dentistry, 91.  Koc Vural, et al. (2022). The effects of resin infiltration on demineralized root surface: An experimental study. European Oral Research, 56(3), 117-123.  Kolumban, et al. (2022). Noninvasive and minimally invasive treatment methods of incipient dental caries used by Romanian dentists. International Journal of Medical Dentistry, 26(3), 411-416.  Krasniqi, et al. (2023). Application of Non-Invasive Methods in the Treatment of White Spot Lesions in Children: A Review Article. International Journal of Biomedicine, 13(4), 228-235.  Krikheli, et al. (2020). The study of infiltration and microabrasion effects on the tooth surface structure on chemical model of focal enamel demineralization. Clinical Dentistry (Russia)(2), 4-8.  Kugel, et al. (2009). Treatment modalities for caries management, including a new resin infiltration system. Compendium of continuing education in dentistry (Jamesburg, N.J. : 1995), 30, 1-10;quiz11.  Kumar, et al. (2012). Resin infiltration-taking the first steps to filling the holes in cheese molars. Annals of the Royal Australasian College of Dental Surgeons, 21, 120-123.  Kumar, et al. (2017). An investigation into the effect of a resin infiltrant on the micromechanical properties of hypomineralised enamel. International Journal of Paediatric Dentistry, 27(5), 399-411.  Kwong, et al. (2000). An ultrastructural study of the application of dentine adhesives to acid-conditioned sclerotic dentine. Journal of Dentistry, 28(7), 515-528.  Körner, et al. (2017). Margin integrity of conservative composite restorations after resin infiltration of demineralized enamel. Journal of Adhesive Dentistry, 19(6), 483-489.  Lasfargues, et al. (2013). Minimal intervention dentistry: Part 6. Caries inhibition by resin infiltration. British Dental Journal, 214(2), 53-59.  Latta. (2007). Shear bond strength and physicochemical interactions of XP bond. Journal of Adhesive Dentistry, 9, 245-248.  Lausch, et al. (2015). Resin infiltration of fissure caries with various techniques of pretreatment in vitro. Caries Research, 49(1), 50-55.  Laverde-Giraldo, et al. (2022). Microabrasión e infiltración como alternativa de tratamiento para Fluorosis Dental severa: reporte de caso. CES odontol, 35(1), 31-46.  Lazar, et al. (2023). White Spot Lesions (WSLs)-Post-Orthodontic Occurrence, Management and Treatment Alternatives: A Narrative Review. Journal of Clinical Medicine, 12(5), 15.  Lee, et al. (2016). Evaluation of stain penetration by beverages in demineralized enamel treated with resin infiltration. Operative Dentistry, 41(1), 93-102.  Lee, et al. (2020). Investigation of the esthetic outcomes of white spot lesion treatments. Nigerian Journal of Clinical Practice, 23(9), 1312-1317.  Lee, et al. (2013). Minimally invasive treatment for esthetic enhancement of white spot lesion in adjacent tooth. Journal of Advanced Prosthodontics, 5(3), 359-363.  Leland, et al. (2016). Evaluation of staining and color changes of a resin infiltration system. Angle Orthodontist, 86(6), 900-904.  Li, et al. (2024). A glycol chitosan derivative with extrafibrillar demineralization potential for self-etch dentin bonding. Dental Materials, 40(2), 327-339.  Li, et al. (2021). In vitro effect of resin infiltrant on resistance of sound enamel surfaces in permanent teeth to demineralization. PeerJ, 9.  Li, et al. (2017). Absence of decorin exacerbates DMM-induced post-traumatic osteoarthritis in mice. Journal of Orthopaedic Research, 35.  Li, et al. (2015). Color stability of carious incipient lesions in the enamel treated with resin infiltration. Chinese Journal of Tissue Engineering Research, 19(38), 6161-6166.  Limvisitsakul, et al. (2024). The color stability of artificial white spot lesions treated with resin infiltration after exposure to staining beverages. BMC Oral Health, 24(1).  Lin, et al. (2022). Effectiveness of resin infiltration in caries inhibition and aesthetic appearance improvement of white – spot lesions: an umbrella review.. Journal of Evidence-Based Dental Practice, 22(3).  Lindquist, et al. (2020). Sealing Proximal Non- and Micro-Cavitated Carious Lesions Using a One-Session Separator Technique: A 2-Year Randomised Clinical Study. Caries Research, 54(5), 483-490.  Liu, et al. (2024). Durability of infiltrated resin application on white spot lesions after different challenges: An ex vivo study. Journal of Prosthetic Dentistry, 131(3), 500-507.  Liu, et al. (2015). Effect of resin infiltration on microhardness of artificial caries lesions. Zhonghua kou qiang yi xue za zhi = Zhonghua kouqiang yixue zazhi = Chinese journal of stomatology, 50(12), 737-741.  Liu, et al. (2013). Comparative study on the penetration abilities of resin infiltration into proximal initial caries lesions in primary molars and permanent posterior teeth. Hua xi kou qiang yi xue za zhi = Huaxi kouqiang yixue zazhi = West China journal of stomatology, 31(2), 161-164.  Liu, et al. (2012). A study on the penetration abilities of natural initial caries lesions with resin infiltration. Hua xi kou qiang yi xue za zhi = Huaxi kouqiang yixue zazhi = West China journal of stomatology, 30(5), 483-486.  Liu, et al. (2011). Limitations in Bonding to Dentin and Experimental Strategies to Prevent Bond Degradation. Journal of Dental Research, 90(8), 953-968.  Liu, et al. (2012). An experimental study on the penetration abilities of resin infiltration into proximal caries lesions in primary molars. Zhonghua kou qiang yi xue za zhi = Zhonghua kouqiang yixue zazhi = Chinese journal of stomatology, 47(11), 684-688.  Lopes, et al. (2024). White spot lesions: diagnosis and treatment - a systematic review. Bmc Oral Health, 24(1), 18.  López, et al. (2019). Effect of conditioning protocols and ultrasonic application of an infiltrant resin in white spot lesions. Brazilian Dental Journal, 30(1), 58-65.  Lu, et al. (2014). Proteoglycans affect monomer infiltration in the etch-and-rinse bonding technique. Dental Materials, 30(11), e289-e299.  Luo, et al. (2023). Effect of polyhydroxy-terminated PAMAM dendrimer on dentin matrix metalloproteinases within the hybrid layers. Bmc Oral Health, 23(1), 12.  Mabrouk, et al. (2020). Erosion Infiltration in the Management of Molar-Incisor Hypomineralization (MIH) Defects. Case Reports in Dentistry, 2020.  Maheswari, et al. (2015). Caries management by risk assessment: A review on current strategies for caries prevention and management. Journal of Pharmacy and Bioallied Sciences, 7(6), S320-S324.  Mandava, et al. (2017). Microhardness and penetration of artificial white spot lesions treated with resin or colloidal silica infiltration. Journal of Clinical and Diagnostic Research, 11(4), JC05-JC09.  Manoharan, et al. (2019). Is Resin Infiltration a Microinvasive Approach to White Lesions of Calcified Tooth Structures?: A Systemic Review. International Journal of Clinical Pediatric Dentistry, 12(1), 53-58.  Mansur, et al. (2020). Effectiveness of enamel infiltration after radiation therapy in patients with oral cavity cancer.. Archiv Euromedica, 10(3), 114-117.  Marinelli, et al. (2021). White spot lesions in orthodontics: Prevention and treatment. a descriptive review. Journal of Biological Regulators and Homeostatic Agents, 35(2), 227-240.  Markowitz, et al. (2018). Assessing the appearance and fluorescence of resin-infiltrated white spot lesions with caries detection devices. Operative Dentistry, 43(1), 10-18.  Marouane, et al. (2020). Transillumination-aided infiltration: A diagnostic concept for treating enamel opacities. Journal of Esthetic and Restorative Dentistry, 32(5), 451-456.  Marouane, et al. (2021). The influence of lesion characteristics on application time of an infiltrate applied to MIH lesions on anterior teeth: An exploratory <i>in vivo</i> pilot study. Journal of Dentistry, 115, 6.  Marović. (2022). INCORPORATION OF ANTIMICROBIAL COPPER-DOPED MESOPOROUS GLASS INTO RESIN COMPOSITES. Acta Stomatologica Croatica, 56(2), 187.  Marró Freitte, et al. (2011). Tratamiento de lesiones de caries interproximales mediante el uso de infiltrantes. Rev. clín. periodoncia implantol. rehabil. oral (Impr.), 4(3), 134-137.  Marshall Jr, et al. (1998). Effect of citric acid concentration on dentin demineralization, dehydration, and rehydration: Atomic force microscopy study. Journal of Biomedical Materials Research, 42(4), 500-507.  Martinez, et al. (2014). Uso de dispositivo sônico para aplicação de sistema adesivo em lesões cervicais não-cariosas Application of an adhesive sys. Full dent. sci, 5(17), 171-178.  Massé, et al. (2023). Infiltrant resin and enamel infractions: two case reports of a novel and minimally invasive approach. Quintessence International, 54(3), 180-185.  Mathias, et al. (2019). Effect of diphenyliodonium hexafluorophosphate salt on experimental infiltrants containing different diluents. Odontology, 107(2), 202-208.  Mattos-Silveira. (2016). Diamino fluoreto de prata - uma nova proposta para o tratamento não operatório de lesões proximais em molares decíduos: estudo clínico randomizado. https://doi.org/doi:  Mattos-Silveira, et al. (2014). New proposal of silver diamine fluoride use in arresting approximal caries: Study protocol for a randomized controlled trial. Trials, 15(1).  Mattos-Silveira, et al. (2015). Children's discomfort may vary among different treatments for initial approximal caries lesions: Preliminary findings of a randomized controlled clinical trial. International Journal of Paediatric Dentistry, 25(4), 300-304.  Mattos-Silveira, et al. (2015). Visible biofilm on approximal surfaces: changes after minimally-invasive treatments. Journal of dental research, 94.  Mattos-Silveira, et al. (2017). Silver diamine fluoride for arresting proximal caries in children. Journal of dental research, 96.  Matuda, et al. (2016). Dental adhesives and strategies for displacement of water/solvents from collagen fibrils. Dental Materials, 32(6), 723-731.  Mazur, et al. (2018). Treatment of post-orthodontic white spot lesions by resin infiltration: A case series. Journal of Stomatology, 71(6), 490-496.  Mazzitelli, et al. (2022). An Insight into Enamel Resin Infiltrants with Experimental Compositions. Polymers, 14(24).  Mazzoni, et al. (2015). Role of dentin MMPs in caries progression and bond stability. Journal of Dental Research, 94(2), 241-251.  Memarpour, et al. (2022). Penetration ability and microhardness of infiltrant resin and two pit and fissure sealants in primary teeth with early enamel lesions. Scientific Reports, 12(1), 9.  Memis Ozgul, et al. (2015). Micro-computed tomographic analysis of progression of artificial enamel lesions in primary and permanent teeth after resin infiltration. Journal of Oral Science, 57(3), 177-183.  Mendes Soares, et al. (2021). Response of pulp cells to resin infiltration of enamel white spot-like lesions. Dental Materials, 37(6), e329-e340.  Meng, et al. (2024). The effect of combined use of resin infiltration with different bioactive calcium phosphate-based approaches on enamel white spot lesions: An in vitro study. Journal of Dentistry, 143.  Meng, et al. (2021). Bonding performance of universal adhesives applied to nano-hydroxyapatite desensitized dentin using etch-and-rinse or self-etch mode. Materials, 14(16).  Mews, et al. (2015). Shear bond strength of orthodontic brackets to enamel after application of a caries infiltrant. Angle Orthodontist, 85(4), 645-650.  Meyer-Lueckel, et al. (2008). Improved resin infiltration of natural caries lesions. Journal of Dental Research, 87(12), 1112-1116.  Meyer-Lueckel, et al. (2010). Infiltration of natural caries lesions with experimental resins differing in penetration coefficients and ethanol addition. Caries Research, 44(4), 408-414.  Meyer-Lueckel, et al. (2016). When and How to Intervene in the Caries Process. Operative Dentistry, 41, S35-S47.  Meyer-Lueckel, et al. (2007). Surface layer erosion of natural caries lesions with phosphoric and hydrochloric acid gels in preparation for resin infiltration. Caries Research, 41(3), 223-230.  Meyer-Lueckel, et al. (2006). Influence of the application time on the penetration of different dental adhesives and a fissure sealant into artificial subsurface lesions in bovine enamel. Dental Materials, 22(1), 22-28.  Miller, et al. (2016). Demineralized white spot lesions: An unmet challenge for orthodontists. Seminars in Orthodontics, 22(3), 193-204.  Min, et al. (2016). Evaluation of resin infiltration using quantitative light-induced fluorescence technology. Photodiagnosis and Photodynamic Therapy, 15, 6-10.  Min, et al. (2015). Evaluation of penetration effect of resin infiltrant using optical coherence tomography. Journal of Dentistry, 43(6), 720-725.  Mine, et al. (2010). Enamel-smear compromises bonding by mild self-etch adhesives. Journal of Dental Research, 89(12), 1505-1509.  Mohamad Ghazi, et al. (2002). Nanoleakage at the dentin adhesive interface: A new application for laser ablation-sector field-ICPMS. Journal of Analytical Atomic Spectrometry, 17(7), 682-687.  Mohamed, et al. (2018). In vitro study of white spot lesion: Maxilla and mandibular teeth. Saudi Dental Journal, 30(2), 142-150.  Mohamed, et al. (2019). A Comparative Study in Penetration Depth of Resin-based Materials into White Spot Lesion. Ain Shams Dental Journal (Egypt), 16(4), 203-211.  Mohamed, et al. (2024). Laser-assisted Icon and clinpro for restoring white spot lesions: an in vitro comparative study. Odontology, 8.  Mohanadass, et al. (2024). Comparative Evaluation of Icon Resin Infiltration and Clinpro XT Varnish Effects on Surface Characteristics in Artificially Induced White Spot Lesions. World Journal of Dentistry, 15(4), 348-355.  Montasser, et al. (2015). In vitro study of the potential protection of sound enamel against demineralization. Progress in Orthodontics, 16(1).  Moosavi, et al. (2022). The Effect of Minimally Invasive Treatments on Enamel Microhardness and Resistance to Further Demineralization. Cumhuriyet Dental Journal, 25(4), 285-290.  Moreira, et al. (2015). Resina infiltrativa para tratamento de lesão de mancha branca: revisão de literatura. Rev. Odontol. Araçatuba (Impr.), 36(1), 30-35.  Morrier. (2014). [White spot lesions and orthodontic treatment. Prevention and treatment]. Orthodontie Francaise, 85(3), 235-244.  Mourao, et al. (2004). Apatite remineralization:: <i>In vivo</i> long term study in dental tissue (Vol. 254). Trans Tech Publications Ltd. https://doi.org/doi:10.4028/www.scientific.net/KEM.254-256.695  Mourão, et al. (2004). Apatite Remineralization: In Vivo Long Term Study in Dental Tissue (Vol. 254). Trans Tech Publications Ltd. https://doi.org/doi:  Mueller, et al. (2011). Surface tridimensional topography analysis of materials and finishing procedures after resinous infiltration of subsurface bovine enamel lesions. Quintessence International, 42(2), 135-147.  Muhsin Yousif Al-Mayali, et al. (2020). Impact of food simulants material on orthodontic bond strength after application of caries infiltrant resin (In vitro study). Medico-Legal Update, 20(3), 979-984.  Mungee, et al. (2024). Minimally invasive resin infiltration with DMG Icon for white spot lesions: A case report. Health Sciences Investigations Journal, 6(1), 826-831.  Mungekar, et al. (2023). Comparison of Resin Infiltration and Pit and Fissure Sealants with Respect to Physical Properties of Demineralized Lesions: An In Vitro Study. World Journal of Dentistry, 14(8), 711-715.  Muñoz, et al. (2013). Alternative esthetic management of fluorosis and hypoplasia stains: Blending effect obtained with resin infiltration techniques. Journal of Esthetic and Restorative Dentistry, 25(1), 32-39.  Muthuvel, et al. (2017). Erosion infiltration technique': A novel alternative for masking enamel white spot lesion. Journal of Pharmacy and Bioallied Sciences, 9(5), S289-S291.  Nagasaki, et al. (2021). Effects of polishing with paste containing surface pre-reacted glass-ionomer fillers on enamel remineralization after orthodontic bracket debonding. Microscopy Research and Technique, 84(2), 171-179.  Nahuelhuaique Fuentealba, et al. (2017). Resin infiltration: An effective and minimally invasive treatment for the treatment of non-cavitated white lesions. narrative review. Avances en Odontoestomatologia, 33(3), 121-126.  Naidu, et al. (2013). Shear bond strength of orthodontic resins after caries infiltrant preconditioning. Angle Orthodontist, 83(2), 306-312.  Nakabayashi, et al. (1998). A tensile test to facilitate identification of defects in dentine bonded specimens. Journal of Dentistry, 26(4), 379-385.  Nakajima, et al. (2005). Elemental distributions and microtensile bond strength of the adhesive interface to normal and caries-affected dentin. Journal of Biomedical Materials Research - Part B Applied Biomaterials, 72(2), 268-275.  Nakajima, et al. (2002). Dimensional changes and ultimate tensile strengths of wet decalcified dentin applied with one-bottle adhesives. Dental Materials, 18(8), 603-608.  Natarajan, et al. (2021). Raman spectroscopic characterisation of resin-infiltrated hypomineralised enamel. Analytical and Bioanalytical Chemistry, 407(19).  Natera-Guarapo, et al. (2023). Typical esthetic outcomes using 5 different modalities to treat MIH affected teeth: a case series. Rev. Fac. Odontol. Univ. Antioq, 35(1), 76-86.  Nayak, et al. (2023). Microinvasive, Esthetic Management of White Spot Lesion Following Orthodontic Treatment Using Resin Infiltration: A Case Report. International Journal of Clinical Pediatric Dentistry, 16(2), 396-399.  Nct. (2011). Treatment of Initial Caries Lesions on Proximal Surfaces of Primary and Permanent Posterior Teeth. https://clinicaltrials.gov/show/NCT01477385.  Nct. (2012). Resin Infiltration and Resin Infiltration With Bleaching in Improving the Esthetics for Fluorosis Stains. https://clinicaltrials.gov/show/NCT01733888.  Nct. (2020). Clinical Evaluation Of Class I Composite Resin Restorations Using Three Different Adhesive Systems. https://clinicaltrials.gov/show/NCT04589416.  Nct. (2020). Non-invasive Caries Management: clinical Study. https://clinicaltrials.gov/ct2/show/NCT04373356.  Nct. (2021). Fluoride Varnish, Ozone, Octenidin and WSLs in Orthodontic Patients. https://clinicaltrials.gov/show/NCT04992481.  Nct. (2021). Resin Infiltration and Fluoride Varnish Lesion Arresting Efficacy on Non-cavitated Proximal Lesion. https://clinicaltrials.gov/show/NCT05202665.  Nct. (2022). Evaluation of the Effect of Self-Assembling Peptide P11-4. https://clinicaltrials.gov/show/NCT05667545.  Nct. (2023). Effect of Poly Amido Amine Dendrimer, Nano-hydroxyapatite. https://clinicaltrials.gov/show/NCT05771077.  Ndokaj, et al. (2021). Treatment of developmental defects of enamel. Clinica Terapeutica, 172(1), 55-56.  Nedeljkovic, et al. (2022). Novel hybrid-glass-based material for infiltration of early caries lesions. Dental Materials, 38(6), 1015-1023.  Nedeljkovic, et al. (2022). Cytotoxicity and anti-biofilm properties of novel hybrid-glass-based caries infiltrant. Dental Materials, 38(12), 2052-2061.  Neres, et al. (2017). Microhardness and roughness of infiltrated white spot lesions submitted to different challenges. Operative Dentistry, 42(4), 428-435.  Neuhaus, et al. (2010). Late Infiltration of Post-orthodontic White Spot Lesions. Journal of Orofacial Orthopedics-Fortschritte Der Kieferorthopadie, 71(6), 442-447.  Neves, et al. (2021). Resin infiltration for approximal caries lesions in primary and permanent teeth: case reports. Rev. Cient. CRO-RJ (Online), 6(1), 56-61.  Ngoc, et al. (2023). Management Of Clear Aligner-related Severe Enamel Demineralization With A Modified Resin Infiltration Technique: A Case Report. Dental Hypotheses, 14(2), 66-68.  Nikolaevna, et al. (2023). Resin infiltration for minimally invasive treatment of initial caries and non-carious spot lesion: literature review. Bulletin of Stomatology and Maxillofacial Surgery, 19(2), 112-123.  Nimbalkar, et al. (2021). Efficacy of Three Enamel Protecting Agents on Shear Bond Strength of Orthodontic Brackets Bonded to Demineralised Enamel with Conventional Adhesive. Journal of Clinical and Diagnostic Research, 15(12), 4.  Nowak-Wachol, et al. (2022). Yttrium Trifluoride as a Marker of Infiltration Rate of Decalcified Root Cementum: An In Vitro Study. Polymers, 14(4).  Nowak-Wachol, et al. (2022). Evaluation of Infiltrant Application in the Course of Root Cementum Caries with Different Methods of Surface Preparation—An In Vitro Study. Coatings, 12(5).  Ntovas, et al. (2018). A clinical guideline for caries infiltration of proximal enamel lesions with resins. British Dental Journal, 225(4), 299-304.  Obadiah, et al. (2020). A survey on the treatment timing and modalities for white spot lesions among various pediatric dentists in India. Drug Invention Today, 13(5), 755-758.  Obead, et al. (2024). The effect of non-invasive treatment techniques on the color masking ability and surface roughness of induced enamel lesions (An <i>in vitro</i> study). Journal of Pharmacy and Bioallied Sciences, 16, 1566-1573.  Obead, et al. (2024). The Effect of Non-Invasive Treatment Techniques on the Color Masking Ability and Surface Roughness of Induced Enamel Lesions (An in vitro Study). Journal of Pharmacy and Bioallied Sciences, 16, S1566-S1573.  Obeid, et al. (2022). Effects of hybrid inorganic-organic nanofibers on the properties of enamel resin infiltrants - An in vitro study. Journal of the Mechanical Behavior of Biomedical Materials, 126, 105067.  Ogodescu, et al. (2014). Micro-CT application for infiltration technology in paedodontics and orthodontics (Vol. 8925). SPIE. https://doi.org/doi:10.1117/12.2044116  Ogodescu, et al. (2012). Visualization of resin penetration into enamel caries lesions of temporary teeth - A confocal microscopic study. Revista de Chimie, 63(1), 82-85.  Ogodescu, et al. (2012). Investigation of an advanced material for the infiltration of white spot lesions in orthodontics (Vol. 188). Trans Tech Publications Ltd. https://doi.org/doi:10.4028/www.scientific.net/SSP.188.87  Oliveira, et al. (2019). Dental bleaching, microabrasion, and resin infiltration: Case report of minimally invasive treatment of enamel hypoplasia. International Journal of Prosthodontics, 33(1), 105-110.  Olmo-González, et al. (2020). Dental management strategies for Molar Incisor Hypomineralization. Pediatric Dental Journal, 30(3), 139-154.  Omoto, et al. (2023). An 8-year follow-up of resin infiltration on anterior white spot lesions. Journal of the Indian Society of Pedodontics and Preventive Dentistry, 41(1), 83-85.  Osorio, et al. (2014). Polymer nanocarriers for dentin adhesion. Journal of Dental Research, 93(12), 1258-1263.  Osorio, et al. (2016). Biomaterials for Catalysed Mineralization of Dental Hard Tissues. Elsevier Inc. https://doi.org/doi:10.1016/B978-1-78242-338-6.00013-2  Osorio, et al. (2018). Zinc is a bioactive element in restorative Dentistry. Annals of Medicine, 50, S4-S5.  Osorio, et al. (2011). Zinc-doped dentin adhesive for collagen protection at the hybrid layer. European Journal of Oral Sciences, 119(5), 401-410.  Osorio, et al. (2013). MMPs activity and bond strength in deciduous dentine-resin bonded interfaces. Journal of Dentistry, 41(6), 549-555.  Osorio, et al. (2012). Experimental resin cements containing bioactive fillers reduce matrix metalloproteinase-mediated dentin collagen degradation. Journal of Endodontics, 38(9), 1227-1232.  Osorio, et al. (2014). Zinc incorporation improves biological activity of beta-tricalcium silicate resin-based cement. Journal of Endodontics, 40(11), 1840-1845.  Palasuk. (2018). Degradation of resin-dentin bonded interface: A review. Journal of International Dental and Medical Research, 11(2), 537-542.  Paris, et al. (2011). Resin infiltration of proximal caries lesions differing in ICDAS codes. European Journal of Oral Sciences, 119(2), 182-186.  Paris, et al. (2009). Validation of two dual fluorescence techniques for confocal microscopic visualization of resin penetration into enamel caries lesions. Microscopy Research and Technique, 72(7), 489-494.  Paris, et al. (2009). Masking of labial enamel white spot lesions by resin infiltration--a clinical report. Quintessence International, 40(9), 713-718.  Paris, et al. (2010). Infiltrants inhibit progression of natural caries lesions in vitro. Journal of Dental Research, 89(11), 1276-1280.  Paris, et al. (2010). Inhibition of caries progression by resin infiltration in situ. Caries Research, 44(1), 47-54.  Paris, et al. (2012). The potential for resin infiltration technique in dental practice. Dental Update, 39(9), 623-628.  Paris, et al. (2016). Resin infiltration after enamel etching. Springer International Publishing. https://doi.org/doi:10.1007/978-3-319-38849-6_10  Paris, et al. (2023). Resin Infiltration. Springer International Publishing. https://doi.org/doi:10.1007/978-3-031-38244-4_11  Paris, et al. (2007). Resin infiltration of artificial enamel caries lesions with experimental light curing resins. Dental Materials Journal, 26(4), 582-588.  Paris, et al. (2007). Resin infiltration of natural caries lesions. Journal of Dental Research, 86(7), 662-666.  Paris, et al. (2013). Masking of white spot lesions by resin infiltration in vitro. Journal of Dentistry, 41, e28-e34.  Paris, et al. (2013). Micro-hardness and mineral loss of enamel lesions after infiltration with various resins: Influence of infiltrant composition and application frequency in vitro. Journal of Dentistry, 41(6), 543-548.  Park, et al. (2024). Resin infiltration for white spot lesions: An in vitro experimental trial. Journal of Orthodontics, 10.  Pashley, et al. (2003). Viscoelastic properties of demineralized dentin matrix. Dental Materials, 19(8), 700-706.  Pashley, et al. (1993). Permeability of dentin to adhesive agents. Quintessence International, 24(9), 618-631.  Pashley, et al. (2001). Aggressiveness of contemporary self-etching adhesives Part II: Etching effects on unground enamel. Dental Materials, 17(5), 430-444.  Patil, et al. (2024). Comparative Evaluation of Zwitterionic Material, Self-assembling Peptide, and Bioactive Glass Incorporated with MI Varnish for Fluoride, Calcium, and Phosphorus Ion Release, Enamel Remineralization, and Microhardness. International Journal of Clinical Pediatric Dentistry, 17, S37-S42.  Paula, et al. (2017). Therapies for White Spot Lesions-A Systematic Review. The Journal of Evidencebased Dental Practice, 17(1), 23-38.  Pecarevic, et al. (2022). Manejo estético de la fluorosis dental: Microabrasión, infiltración de resina y clareamiento externo. Int. j interdiscip. dent. (Print), 15(2), 157-160.  Pedreira, et al. (2023). Radiopacity and physical properties evaluation of infiltrants with Barium and Ytterbium addition. Braz Dent J, 34(4), 93-106.  Pedreira, et al. (2021). Influence of Incorporating Zirconium- and Barium-based Radiopaque Filler Into Experimental and Commercial Infiltrants. Operative Dentistry, 46(5), 566-576.  Pedreira, et al. (2021). Conservative Treatment of Interproximal Incipient Caries Lesions by Resin Infiltration. Brazilian Journal of Oral Sciences, 20, 1-8.  Peng, et al. (2016). The effect of resin infiltration vs. fluoride varnish in enhancing enamel surface conditions after interproximal reduction. Dental Materials Journal, 35(5), 756-761.  Perdigão. (2020). Resin infiltration of enamel white spot lesions: An ultramorphological analysis. Journal of Esthetic and Restorative Dentistry, 32(3), 317-324.  Perdigão, et al. (2017). Masking of enamel fluorosis discolorations and tooth misalignment with a combination of at-home whitening, resin infiltration, and direct composite restorations. Operative Dentistry, 42(4), 347-356.  Perdigäo, et al. (1999). Dentin bonding - Questions for the new millennium. Journal of Adhesive Dentistry, 1(3), 191-209.  Pereira Junior. (2015). Selamento de lesões de cárie proximal com infiltrante resinoso: acompanhamento de 3 anos de um estudo clínico randomizado. https://doi.org/doi:  Pereira, et al. (2024). Bleaching as a complement to fluoride-enhanced remineralization or resin infiltration in masking white spot lesions. J Appl Oral Sci, 32, e20240097-e20240097.  Pérez, et al. (2014). Confocal laser microscopy analysis of resin infiltration in fluorotic teeth. Rev. clín. periodoncia implantol. rehabil. oral (Impr.), 7(2), 53-58.  Pia̧tek-Jakubek, et al. (2014). Effectiveness of selected products in masking white spot lesions on smooth surfaces of teeth. in vitro studies. Journal of Stomatology, 67(3), 330-345.  Piątek-Jakubek, et al. (2017). Influence of infiltration technique and selected demineralization methods on the roughness of demineralized enamel: An in vitro study. Advances in Clinical and Experimental Medicine, 26(8), 1179-1188.  Pomacóndor-Hernández, et al. (2020). Infiltrantes para tratamiento estético de lesiones de manchas blancas por fluorosis: Reporte de caso. Odovtos (En línea), 22(3).  Pomacóndor-Hernández, et al. (2020). Infiltrants for Aesthetic Treatment of White Spots Lesions by Fluorosis: Case Report. Odovtos - International Journal of Dental Sciences, 22(3), 43-49.  Pongprueksa, et al. (2014). Effect of dentinal tubule orientation on the modulus of elasticity of resin-infiltrated demineralized dentin. Dental Materials Journal, 33(1), 54-58.  Pozos-Guillén, et al. (2021). Management Of Dental Caries Lesions In Latin American And Caribbean Countries. Brazilian Oral Research, 35, 1-22.  Prajapati, et al. (2017). Effect of Resin Infiltration on Artificial Caries: An in vitro Evaluation of Resin Penetration and Microhardness. Jaypees International Journal of Clinical Pediatric Dentistry, 10(3), 250-256.  Prasada, et al. (2018). Spectrophotometric evaluation of white spot lesion treatment using novel resin infiltration material (ICON ®). Journal of Conservative Dentistry, 21(5), 531-535.  Prati, et al. (1999). Thickness and morphology of resin-infiltrated dentin layer in young, old, and sclerotic dentin. Operative dentistry, 24(2), 66-72.  Prati, et al. (1998). Resin-infiltrated dentin layer formation of new bonding systems. Operative Dentistry, 23(4), 185-194.  Prati, et al. (1999). Effect of removal of surface collagen fibrils on resin-dentin bonding. Dental Materials, 15(5), 323-331.  Prati, et al. (1995). Dentine permeability and bond quality as affected by new bonding systems. Journal of Dentistry, 23(4), 217-226.  Predapramote, et al. (2024). Evaluation of resin infiltration for inhibiting initial caries progression: An in vitro study using Micro-Computed Tomographic analysis. Saudi Dental Journal, 36(5), 745-750.  Priyadarshini, et al. (2017). PLGA nanoparticles as chlorhexidine-delivery carrier to resin-dentin adhesive interface. Dental Materials, 33(7), 830-846.  Prodan, et al. (2022). Development of New Experimental Dental Enamel Resin Infiltrants-Synthesis and Characterization. Materials, 15(3), 16.  Provenzano, et al. (2023). In vitro effect of two resin based materials for treating initial caries lesion around braces, under cariogenic challenge. Journal of Clinical and Experimental Dentistry, 15(12), e991-e998.  Puleio, et al. (2022). Systematic Review on White Spot Lesions Treatments. European Journal of Dentistry, 16(1), 41-48.  Qibi, et al. (2023). Influence of resin infiltration pretreatment on the microleakage under orthodontic bracket (an in vitro study). Journal of Orthodontic Science, 12(43).  Rabiah, et al. (2019). Resin Infiltration Recent Trends in Aesthetic and Restorative Dentistry: A Systematic Review. International Journal of Medical Research & Health Sciences, 8(11), 85-89.  Radwan. (2023). Noninvasive Proximal Adhesive Restoration in the Treatment of Non-cavitated Interproximal Incipient Carious Lesions: A Case Report. Cureus Journal of Medical Science, 15(7), 8.  Rahimi, et al. (2017). Efficacy of fluoride varnish for prevention of white spot lesions during orthodontic treatment with fixed appliances: A systematic review study. Biomedical Research and Therapy, 4(8), 1513-1526.  Rahiotis, et al. (2015). Setting characteristics of a resin infiltration system for incipient caries treatment. Journal of Dentistry, 43(6), 715-719.  Rai, et al. (2016). Qualitative and quantitative effect of a protective chlorhexidine varnish layer over resin-infiltrated proximal carious lesions in primary teeth. Pediatric Dentistry, 38(4), E40-E45.  Raj, et al. (2023). Remineralization Agents in Orthodontics: Systematic Review. International Journal of Chemical and Biochemical Sciences, 24(4), 370-381.  Rajasekaran, et al. (2015). Resin infiltration technique for arresting white spot lesion: Case report. Biomedical and Pharmacology Journal, 8, 225-228.  Rana, et al. (2021). A comparative evaluation of penetration depth and surface microhardness of Resin Infiltrant, CPP-ACPF and Novamin on enamel demineralization after banding: an in vitro study. Biomaterial Investigations in Dentistry, 8(1), 64-71.  Reis, et al. (2016). Evaluation of bond strength of composite resin to enamel demineralized, exposed to remineralization and subjected to caries infiltration. Braz. dent. sci, 19(1), 48-54.  Resende, et al. (2024). Icon for the treatment of postorthodontic white spot lesions. 2-year follow-up. The International Journal of Esthetic Dentistry, 19(4), 336-347.  Revilla-León, et al. (2019). Silicone Additive Manufactured Indices Performed from a Virtual Diagnostic Waxing for Direct Composite Diastema Closure Combined with Resin Infiltration Technique on White Spot Lesions: A Case Report. Journal of Prosthodontics, 28(8), 855-860.  Rey Duro, et al. (2012). Current treatment of white spot lesions. Medicina Oral, Patologia Oral y Cirugia Bucal, 17.  Rey, et al. (2014). Evaluation Of the staining potential of a caries infiltrant in comparison to other products. Dental Materials Journal, 33(1), 86-91.  Ritwik, et al. (2016). Hydrolytic and color stability of resin infiltration: A preliminary in vitro trial. Journal of Contemporary Dental Practice, 17(5), 377-381.  Rocha, et al. (2020). Effectiveness of home bleaching treatment after resin infiltrant application. Oral Health and Preventive Dentistry, 18(3), 549-554.  Roig-Vanaclocha, et al. (2020). Dental treatment of white spots and a description of the technique and digital quantification of the loss of enamel volume. Applied Sciences (Switzerland), 10(12).  Rojas-Gómez, et al. (2021). Infiltración de resina y barniz de flúor para el tratamiento de caries interproximales no cavitadas en dentición temporal. Int. j interdiscip. dent. (Print), 14(1), 100-104.  Román-Rodríguez, et al. (2020). A tooth whitening and chemical abrasive protocol for the treatment of developmental enamel defects. Journal of Prosthetic Dentistry, 123(3), 379-383.  Rosianu, et al. (2017). Low viscosity resin penetration degree in incipient caries lesions. Revista de Chimie, 68(11), 2588-2592.  Ryou, et al. (2013). A characterization of the mechanical behavior of resin-infiltrated dentin using nanoscopic Dynamic Mechanical Analysis. Dental Materials, 29(7), 719-728.  Sabti, et al. (2024). Evaluating color stability and enamel surface roughness following resin infiltration treatment. Clinical and Experimental Dental Research, 10(1).  Saccucci, et al. (2022). Assessment of Enamel Color Stability of Resins Infiltration Treatment in Human Teeth: A Systematic Review. International Journal of Environmental Research and Public Health, 19(18), 19.  Sadikoglu, et al. (2022). Can the hydrogel form of sodium ascorbate be used to reverse compromised resin infiltrant penetration after bleaching? Nigerian Journal of Clinical Practice, 25(4), 509-515.  Sadikoglu. (2020). White Spot Lesions: Recent Detection and Treatment Methods. Cyprus Journal of Medical Sciences, 5(3), 260-266.  Sadikoǧlu. (2023). Using Resin Infiltration Technique and Direct Composite Restorations for the Treatment of Carious Lesions with Different Depths. Case Reports in Dentistry, 2023.  Sadyrin, et al. (2020). Characterization of Enamel and Dentine about a White Spot Lesion: Mechanical Properties, Mineral Density, Microstructure and Molecular Composition. Nanomaterials, 10(9), 17.  Sadyrin. (2023). Influence of a polymeric infiltrant on the density of enamel white spot lesions. Izvestiya of Saratov University Mathematics Mechanics Informatics, 23(1), 83-94.  Saitoh, et al. (2021). Molar incisor hypomineralization: A review and prevalence in Japan. Japanese Dental Science Review, 57, 71-77.  Salama, et al. (2021). Comparing the effect of two different remineralizing agents on shear bond strength of orthodontics brackets. International Journal of Dentistry and Oral Science, 8(4), 2134-2139.  Saluja, et al. (2022). Minimally invasive management of white spot lesion using resin infiltration technique: A case report. Gulhane Medical Journal, 64(1), 120-122.  Sammarco. (2019). Combined minimally invasive treatment of white and brown fluorotic discolorations in a teenager: a case report. The international journal of esthetic dentistry, 14(2), 148-155.  Sánchez Aguilera, et al. (2004). Control of the collagen network collapse: Self-etching bonding systems. Avances en Odontoestomatologia, 20(4), 175-183.  Sandoval, et al. (2016). Management of post-orthodontic white-spot-lesions: clinical handling of the resin infiltration technique (Icon®, DMG). Int. j. odontostomatol. (Print), 10(1), 29-33.  Sanfelice, et al. (2024). Short-term Results of the Masking Effect of an Infiltrant Resin on Mild Molar Incisor Hypomineralization Lesions in Anterior Teeth. Operative Dentistry, 49(1), 9.  Sano, et al. (1995). Tensile Properties of Resin-infiltrated Demineralized Human Dentin. Journal of Dental Research, 74(4), 1093-1102.  Sant'anna, et al. (2016). Infiltrante resinoso vs Microabrasão no manejo de lesões de mancha branca: relato de caso. Rev. Assoc. Paul. Cir. Dent, 70(2), 187-190.  Santi, et al. (2022). Evaluation of pretreatments on intra-radicular dentin bond strength of self-adhesive resin cements. J Esthet Restor Dent, 34(7), 1051-1059.  Sarti. (2015). Selamento de lesões cariosas proximais em molares decíduos com infiltrante resinoso: estudo clínico controlado de boca dividida. https://doi.org/doi:  Sarti, et al. (2020). Two-Year Split-Mouth Randomized Controlled Clinical Trial on the Progression of Proximal Carious Lesions on Primary Molars After Resin Infiltration. Pediatr Dent, 42(2), 110-115.  Sato, et al. (2005). Influence of NaOCl treatment of etched and dried dentin surface on bond strength and resin infiltration. Operative Dentistry, 30(3), 353-358.  Sato, et al. (2005). Comparison of depth of dentin etching and resin infiltration with single-step adhesive systems. Journal of Dentistry, 33(6), 475-484.  Sattabanasuk, et al. (2009). Resin Bonding to Dentine After Casein Phosphopeptide-Amorphous Calcium Phosphate (CPP-ACP) Treatments. Journal of Adhesion Science and Technology, 23(7), 1149-1161.  Sattabanasuk, et al. (2005). Bonding of resin to artificially carious dentin. Journal of Adhesive Dentistry, 7(3), 183-192.  Sauro, et al. (2010). Resin-dentin bonds to EDTA-treated vs. acid-etched dentin using ethanol wet-bonding. Dental Materials, 26(4), 368-379.  Schmidlin, et al. (2012). Protection of sound enamel and artificial enamel lesions against demineralisation: Caries infiltrant versus adhesive. Journal of Dentistry, 40(10), 851-856.  Schnabl, et al. (2019). Testing the Clinical Applicability of Resin Infiltration of Developmental Enamel Hypomineralization Lesions Using an In Vitro Model. International Journal of Clinical Pediatric Dentistry, 12(2), 126-132.  Schneider, et al. (2017). Imaging resin infiltration into non-cavitated carious lesions by optical coherence tomography. Journal of Dentistry, 60, 94-98.  Schoppmeier, et al. (2018). Power bleaching enhances resin infiltration masking effect of dental fluorosis. A randomized clinical trial. Journal of Dentistry, 79, 77-84.  Sevbitov, et al. (2020). Icon as a method of choice for injectable treatment of initial caries in patients with fibrodysplasia ossificans progressive: A clinical case. Journal of Global Pharma Technology, 12(2), 270-274.  Sevbitov, et al. (2019). Analysis of electronic microscopy results based on combining the infiltration method with different restoration technologies and in vitro investigation of enamel focal demineralization treatment at the defect stage. Periodico Tche Quimica, 16(33), 53-59.  Sfalcin, et al. (2017). Influence of bioactive particles on the chemical-mechanical properties of experimental enamel resin infiltrants. Clinical Oral Investigations, 21(6), 2143-2151.  Shahmoradi, et al. (2022). Mechanical failure of posterior teeth due to caries and occlusal wear- A modelling study. Journal of the Mechanical Behavior of Biomedical Materials, 125.  Shaik, et al. (2017). Quantitative analysis of remineralization of artificial carious lesions with commercially available newer remineralizing agents using SEM-EDX- in vitro study”. Journal of Clinical and Diagnostic Research, 11(4), ZC20-ZC23.  Shan, et al. (2022). Retention of Intrafibrillar Minerals Improves Resin–Dentin Bond Durability. Journal of Dental Research, 101(12), 1490-1498.  Shibatani, et al. (1989). Basic study of fluoride-releasing adhesive resin for prevention of root caries: 2. Enhancement of acid resistance of the dentin. The Journal of Osaka University Dental School, 29, 109-116.  Shimada, et al. (2020). 3D imaging of proximal caries in posterior teeth using optical coherence tomography. Scientific Reports, 10(1), 14.  Shivanna, et al. (2011). Novel treatment of white spot lesions: A report of two cases. Journal of Conservative Dentistry, 14(4), 423-426.  Shu, et al. (2024). Aesthetic impact of resin infiltration and its mechanical effect on ceramic bonding for white spot lesions. BMC Oral Health, 24(1).  Silva, et al. (2024). Effect of Toothbrushing with Different Dentifrices on the Surface of an Infiltrant Resin Used to Inactivate White Spot Lesions. Pesquisa Brasileira em Odontopediatria e Clinica Integrada, 24.  Silva, et al. (2020). Monitoring enamel caries on resin-treated occlusal surfaces using quantitative light-induced fluorescence: an in vitro study. Lasers in Medical Science, 35(7), 1629-1636.  Silva, et al. (2018). Color restoration and stability in two treatments for white spot lesions. The international journal of esthetic dentistry, 13(3), 394-403.  Silva, et al. (2018). Staining potential differences between an infiltrative resin and an esthetic, flowable composite. Journal of Esthetic and Restorative Dentistry, 30(5), 457-463.  Silva, et al. (2020). Sealing Carious Fissures with Resin Infiltrant in Association with a Flowable Composite Reduces Immediate Microleakage? Pesqui. bras. odontopediatria clín. integr, 20, e5114-e5114.  Sinanovic, et al. (2024). Effect of Bleaching on Resin-Infiltration-Masked Artificial White Spots In Vitro. Journal of Functional Biomaterials, 15(5).  Singh, et al. (2015). Viscoelastic properties of collagen-adhesive composites under water-saturated and dry conditions. Journal of Biomedical Materials Research Part A, 103(2), 646-657.  Skucha-Nowak. (2015). Attempt to assess the infiltration of enamel made with experimental preparation using a scanning electron microscope. Open Medicine (Poland), 10(1), 238-248.  Skucha-Nowak, et al. (2015). Natural and Controlled Demineralization for Study Purposes in Minimally Invasive Dentistry. Advances in Clinical and Experimental Medicine, 24(5), 891-898.  Skucha-Nowak, et al. (2016). Assessing the penetrating abilities of experimental preparation with dental infiltrant features using optical microscope: Preliminary study. Advances in Clinical and Experimental Medicine, 25(5), 961-969.  Skucha-Nowak, et al. (2016). Using an electron scanning microscope to assess the penetrating abilities of an experimental preparation with features of a dental infiltrant: Preliminary study. Advances in Clinical and Experimental Medicine, 25(6), 1293-1301.  Skucha-Nowak, et al. (2020). Use of ytterbium trifluoride in the field of microinvasive dentistry-An in vitro preliminary study. Coatings, 10(10).  Solanki, et al. (2023). Management of Turner's Hypoplasia Using Resin Infiltration: A Case Report. Cureus Journal of Medical Science, 15(11), 7.  Solomon, et al. (2022). Minimalistic Intervention of White Spot Lesions and Dental Fluorosis with Resin Infiltration Technique- A Report of Two Cases. Journal of Clinical and Diagnostic Research, 16(8), ZD1-ZD4.  Sonesson, et al. (2017). Management of post-orthodontic white spot lesions: an updated systematic review. European journal of orthodontics, 39(2), 116-121.  Souza, et al. (2023). Effects of the Incorporation of Bioactive Particles on Physical Properties, Bioactivity and Penetration of Resin Enamel Infiltrant. Clinical, Cosmetic and Investigational Dentistry, 15, 31-43.  Souza, et al. (2024). Brazilian dentist's knowledge of minimum intervention dentistry for caries management: application of a developed knowledge scale (MIDDeC-KS) and evaluation of its psychometric properties. Eur Arch Paediatr Dent, 25(1), 27-38.  Soveral, et al. (2021). Effect of resin infiltration on enamel: A systematic review and meta-analysis. Journal of Functional Biomaterials, 12(3).  Soxman. (2010). Improving caries diagnosis and early intervention in the primary and young permanent dentition. General Dentistry, 58(3), 188-193.  Splieth, et al. (2010). Sealants in Dentistry: Outcomes of the ORCA Saturday Afternoon Symposium 2007. Caries Research, 44(1), 3-13.  Splieth, et al. (2020). How to intervene in the caries process: proximal caries in adolescents and adults—a systematic review and meta-analysis. Clinical Oral Investigations, 24(5), 1623-1636.  Srikumar, et al. (2024). An in vitro evaluation of Icon resin infiltrant penetration into demineralized enamel lesions using an indirect staining technique with confocal laser scanning microscope analysis in dual fluorescence mode. Journal of Conservative Dentistry and Endodontics, 27(4), 366-372.  Stape, et al. (2018). A novel dry-bonding approach to reduce collagen degradation and optimize resin-dentin interfaces. Scientific Reports, 8(1).  Su, et al. (2021). Effect of Thermal Cycling Aging on the Surface Microhardness and Roughness of Resin-Infiltrated Enamel Lesions. Science of Advanced Materials, 13(3), 356-363.  Subramaniam, et al. (2014). Evaluation of penetration depth of a commercially available resin infiltrate into artificially created enamel lesions: An in vitro study. Journal of Conservative Dentistry, 17(2), 146-149.  Suda, et al. (2018). Application of the Self-Assembling Peptide P11-4 for Prevention of Acidic Erosion. Operative Dentistry, 43(4), E166-E172.  Swamy, et al. (2017). In vitro evaluation of resin infiltrant penetration into white spot lesions of deciduous molars. Journal of Clinical and Diagnostic Research, 11(9), ZC46-ZC49.  Takashino, et al. (2016). Effect of thermal cyclic stress on acid resistance of resin-infiltrated incipient enamel lesions in vitro. Dental Materials Journal, 35(3), 425-431.  Talungchit, et al. (2014). Ethanol-wet bonding and chlorhexidine improve resin-dentin bond durability: Quantitative analysis using Raman spectroscopy. Journal of Adhesive Dentistry, 16(5), 441-450.  Tanany, et al. (2022). Effect of resin infiltration and bleaching on surface roughness and microhardness of human enamel with induced white spot lesions. Brazilian Dental Science, 25(2).  Taraboanta, et al. (2019). Evaluation of some exogenous colorants effects on resin based materials used in incipient caries lesions therapy. Materiale Plastice, 56(3), 629-634.  Tavares, et al. (2021). Resin infiltration in white spot lesions caused by orthodontic hypomineralisation: a minimally invasive therapy. British Dental Journal, 231(7), 387-392.  Tawakoli, et al. (2016). Oral biofilm and caries-infiltrant interactions on enamel. Journal of Dentistry, 48, 40-45.  Tay, et al. (2000). Mechanical Disruption of Dentin Collagen Fibrils during Resin-Dentin Bond Testing. Journal of Adhesive Dentistry, 2(3), 175-192.  Tay, et al. (1996). Micromorphological spectrum from overdrying to overwetting acid-conditioned dentin in water-free, acetone-based, single-bottle primer/adhesives. Dental Materials, 12(4), 236-244.  Tay, et al. (2006). Ultrastructure of Intraradicular Dentin After Irrigation with BioPure MTAD. II. The Consequence of Obturation with an Epoxy Resin-Based Sealer. Journal of Endodontics, 32(5), 473-477.  Tay, et al. (1997). Ultrastructure of the resin-dentin interface following reversible and irreversible rewetting. American Journal of Dentistry, 10(2), 77-82.  Tay, et al. (2002). How can nanoleakage occur in self-etching adhesive systems that demineralize and infiltrate simultaneously? Journal of Adhesive Dentistry, 4(4), 255-269.  Tay, et al. (2005). Etched enamel structure and topography: Interface with materials. Springer Berlin Heidelberg. https://doi.org/doi:10.1007/3-540-28559-8_1  Tedesco, et al. (2022). Nonrestorative treatment of initial caries lesion in primary teeth: a systematic review and network meta-analysis. Acta Odontologica Scandinavica, 80(1), 1-8.  Tereza. (2015). Influência da remoção do excesso de materiais adesivos sobre o esmalte erodido, na resistência a desafio erosivo in vitro. https://doi.org/doi:  Tezvergil-Mutluay, et al. (2014). Zoledronate and ion-releasing resins impair dentin collagen degradation. Journal of Dental Research, 93(10), 999-1004.  Theodory, et al. (2019). Masking and penetration ability of various sealants and icon in artificial initial caries lesions in vitro. Journal of Adhesive Dentistry, 21(3), 265-272.  Tinanoff, et al. (2015). Evidence-based update of pediatric dental restorative procedures: Preventive strategies. Journal of Clinical Pediatric Dentistry, 39(3), 193-197.  Tirlet, et al. (2013). Infiltration, a new therapy for masking enamel white spots: a 19-month follow-up case series. The European journal of esthetic dentistry : official journal of the European Academy of Esthetic Dentistry, 8(2), 180-190.  Titley, et al. (1995). An SEM examination of etched dentin and the structure of the hybrid layer. Journal (Canadian Dental Association), 61(10), 887-894.  Tituana-Yupangui, et al. (2023). Rehabilitation Treatment in Pediatric Patients with Molar Incisor Hypomineralization: A Scoping Review. Pesqui. bras. odontopediatria clín. integr, 23, e220112-e220112.  Tiunova, et al. (2022). Rationale behind minimally invasive approach in the treatment of dental fluorosis. New Armenian Medical Journal, 16(1), 87-93.  Tiuraniemi, et al. (2021). Success of resin infiltration treatment on interproximal tooth surfaces in young adults-A practice-based follow-up study. Clinical & Experimental Dental Research, 7(2), 189-195.  Todorova, et al. (2020). Aesthetic Improvement of White Spot Fluorosis Lesions with Resin Infiltration. Folia Medica, 62(1), 208-213.  Tokuc, et al. (2024). An in vitro evaluation of the effects of fluoride, CPP-ACP, or resin infiltration on discoloration caused by pediatric supplements. Quintessence International, 55(2), 148-158.  Toledano, et al. (2013). In vitro load-induced dentin collagen-stabilization against MMPs degradation. Journal of the Mechanical Behavior of Biomedical Materials, 27, 10-18.  Toledano, et al. (2016). Nanoscopic dynamic mechanical analysis of resin-infiltrated dentine, under in vitro chewing and bruxism events. Journal of the Mechanical Behavior of Biomedical Materials, 54, 33-47.  Toledano, et al. (2023). Dexamethasone-doped nanoparticles improve mineralization, crystallinity and collagen structure of human dentin. Journal of Dentistry, 130.  Toledano, et al. (2017). Mechanical loading influences the viscoelastic performance of the resin-carious dentin complex. Biointerphases, 12(2), 11.  Toledano, et al. (2017). Ions-modified nanoparticles affect functional remineralization and energy dissipation through the resin-dentin interface. Journal of the Mechanical Behavior of Biomedical Materials, 68, 62-79.  Toledano, et al. (2011). Bleaching agents increase metalloproteinases-mediated collagen degradation in Dentin. Journal of Endodontics, 37(12), 1668-1672.  Toledano, et al. (2012). Zinc-inhibited MMP-mediated collagen degradation after different dentine demineralization procedures. Caries Research, 46(3), 201-207.  Toledano, et al. (2012). A ZnO-doped adhesive reduced collagen degradation favouring dentine remineralization. Journal of Dentistry, 40(9), 756-765.  Toledano-Osorio, et al. (2018). Improved reactive nanoparticles to treat dentin hypersensitivity. Acta Biomaterialia, 72, 371-380.  Topoliceanu, et al. (2013). Chemical Changes. of Enamel Occlusal Surfaces Affected by Incipient Dental Caries: an EDX Study. Revista De Chimie, 64(11), 1324-1328.  Tori. (2021). Resin InResin Infiltration Concept - Need of an Hourfiltration Concept - Need of an Hour. Journal of Research in Medical and Dental Science, 9(11), 1-5.  Torres, et al. (2015). Color masking of developmental enamel defects: A case series. Operative Dentistry, 40(1), 25-33.  Torres, et al. (2011). Effect of caries infiltration technique and fluoride therapy on the colour masking of white spot lesions. Journal of Dentistry, 39(3), 202-207.  Torres, et al. (2012). Effect of caries infiltration technique and fluoride therapy on microhardness of enamel carious lesions. Operative Dentistry, 37(4), 363-369.  Torres, et al. (2019). Fluorescence properties of demineralized enamel after resin infiltration and dental bleaching. American Journal of Dentistry, 32(1), 43-46.  Torres-Rabello, et al. (2021). Tratamiento estético de defecto de esmalte utilizando tratamiento combinado con resina infiltrante: Reporte de caso. Int. j interdiscip. dent. (Print), 14(2), 177-180.  Tostes, et al. (2014). Effect of resin infiltration on the nanomechanical properties of demineralized bovine enamel. Indian Journal of Dentistry, 5(3), 116-122.  Tóth, et al. (2011). Immunohistochemistry on epoxy resin-embedded bone marrow biopsy experience with 936 cases. Applied Immunohistochemistry and Molecular Morphology, 19(1), 15-20.  Toti, et al. (2022). White Spots Prevalence and Tooth Brush Habits during Orthodontic Treatment. Healthcare, 10(2), 8.  Triwardhani, et al. (2020). Effect of different white-spot lesion treatment on orthodontic shear strength and enamel morphology: In vitro study. Journal of International Oral Health, 12(2), 120-128.  Tulsani, et al. (2019). Knowledge and awareness about resin infiltration: A micro-invasive treatment for white spot lesions. International Journal of Dentistry and Oral Science, 2, 46-53.  Turska-Szybka, et al. (2014). Resin infiltration of natural caries lesions on smooth surfaces of primary teeth – In vitro studies. Dental and Medical Problems, 51(3), 308-317.  Ulrich, et al. (2015). Tridimensional surface roughness analysis after resin infiltration of (deproteinized) natural subsurface carious lesions. Clinical Oral Investigations, 19(6), 1473-1483.  Urquhart, et al. (2019). Nonrestorative Treatments for Caries: Systematic Review and Network Meta-analysis. Journal of Dental Research, 98(1), 14-26.  Van Meerbeek, et al. (1997). Epoxy-embedded versus nonembedded TEM examination of the resin-dentin interface. Journal of Biomedical Materials Research, 35(2), 191-197.  Van Meerbeek, et al. (1998). A TEM study of two water-based adhesive systems bonded to dry and wet dentin. Journal of Dental Research, 77(1), 50-59.  Vas, et al. (2024). Self-Assembling Peptide P11-4 For Management Of White Spot Lesions In Subjects Undergoing Orthodontic Treatment. - A Structured Review. International Journal of Orthodontic Rehabilitation, 15(2), 13-25.  Véguer, et al. (2025). Postoperative stability of aesthetic outcomes following resin infiltration of hypomineralised and demineralised enamel lesions: a systematic review. Journal of Evidence-Based Dental Practice, 25(1).  Veli, et al. (2016). Effects of different pre-treatment methods on the shear bond strength of orthodontic brackets to demineralized enamel. Acta Odontologica Scandinavica, 74(1), 7-13.  Vennat, et al. (2014). Three-dimensional pore-scale modelling of dentinal infiltration. Computer Methods in Biomechanics and Biomedical Engineering, 17(6), 632-642.  Vennat, et al. (2015). A natural biomimetic porous medium mimicking hypomineralized enamel. Dental Materials, 31(3), 225-234.  Venu Babu, et al. (2024). Nanoparticles Induced Biomimetic Remineralization of Acid-Etched Dentin. Journal of Dentistry (Iran), 25(4), 359-368.  Vianna, et al. (2016). Bonding brackets on white spot lesions pretreated by means of two methods. Dental Press Journal of Orthodontics, 21(2), 39-44.  Vinothkumar. (2021). Application of Near-infrared Light Transillumination in Restorative Dentistry: A Review. Journal of Contemporary Dental Practice, 22(11), 1355-1361.  Viteri García, et al. (2016). Estudio de la microdureza de dientes permanentes con fluorosis incipiente, tratados con resina infiltrante. Odontología (Ecuad.), 18(2), 6-11.  Vola. (2014). Influencia de los inhibidores de las metaloproteinasas, agentes reticuladores y remineralización biomimética en la longevidad de la unión adhesiva. Parte 1: Inhibidores de las metaloproteinasas. Actas odontol, 11(2), 10-21.  Wahba, et al. (2022). Preventing and arresting primary tooth enamel lesions using self-assembling peptide P11-4 in vitro. Journal of International Society of Preventive and Community Dentistry, 12(1), 58-70.  Walker, et al. (2000). Influence of additional acid etch treatment on resin cement dentin infiltration. Journal of Prosthodontics, 9(2), 77-81.  Wang, et al. (2017). Type III collagen is critical to the proper functioning of knee cartilage and meniscus. Journal of Orthopaedic Research, 35.  Wang, et al. (2021). Experimental self-etching resin infiltrants on the treatment of simulated carious white spot lesions. Journal of the Mechanical Behavior of Biomedical Materials, 113.  Wang, et al. (2023). Applications of collagen extrafibrillar demineralization in dentin bonding. Chinese Journal of Stomatology, 58(1), 81-85.  Wang, et al. (2020). Minimally invasive esthetic management of dental fluorosis: a case report. Journal of International Medical Research, 48(10), 7.  Wang, et al. (2007). Effect of solvent content on resin hybridization in wet dentin bonding. Journal of Biomedical Materials Research - Part A, 82(4), 975-983.  Wang, et al. (2023). The remineralization effect of GERM CLEAN on early human enamel caries lesions in vitro. Scientific Reports, 13(1), 8.  Wang, et al. (2024). STMP and PVPA as Templating Analogs of Noncollagenous Proteins Induce Intrafibrillar Mineralization of Type I Collagen via PCCP Process. Advanced Healthcare Materials, 13(20).  Warner, et al. (2022). Making white spots disappear! Do minimally invasive treatments improve incisor opacities in children with molar-incisor hypomineralisation? International Journal of Paediatric Dentistry, 32(4), 617-625.  Warreth. (2023). Dental Caries and Its Management. International Journal of Dentistry, 2023, 15.  Weyland, et al. (2022). Management of white spot lesions induced during orthodontic treatment with multibracket appliance: a national-based survey. Clinical Oral Investigations, 26(7), 4871-4883.  Wiedemeyer, et al. (2024). White Spot Lesions, Dental Root Resorption and Medications during Orthodontic Therapy. Informationen Aus Orthodontie Und Kieferorthopaedie, 56(3), 153-158.  Wiegand, et al. (2011). Adhesive performance of a caries infiltrant on sound and demineralised enamel. Journal of Dentistry, 39(2), 117-121.  Wierichs, et al. (2023). Short-Term efficacy of caries resin infiltration during treatment with orthodontic fixed appliances. A randomized controlled trial. European Journal of Orthodontics, 45(2), 115-121.  Wierichs, et al. (2021). Efficacy of a self-assembling peptide to remineralize initial caries lesions - A systematic review and meta-analysis. Journal of Dentistry, 109, 7.  Wierichs, et al. (2017). Effects of Self-Assembling Peptide P11-4, Fluorides, and Caries Infiltration on Artificial Enamel Caries Lesions in vitro. Caries Research, 51(5), 451-459.  Winter. (2016). Treating post-orthodontic white spots: A conservative resin infiltration technique. Dentistry Today, 35(10).  Wu, et al. (2020). Effects of different anti-caries procedures on microhardness and micromorphology of irradiated permanent enamel. Dental Materials Journal, 39(1), 118-125.  Xie, et al. (2023). Comparison of therapies of white spot lesions: a systematic review and network meta-analysis. BMC Oral Health, 23(1).  xsg. (2016). Monitoring of caries lesions among primary teeth after block with resin infiltrant. https://trialsearch.who.int/Trial2.aspx?TrialID=RBR-954xsg.  Yadav, et al. (2019). A comparative quantitative & qualitative assessment in orthodontic treatment of white spot lesion treated with 3 different commercially available materials - In vitro study. Journal of Clinical and Experimental Dentistry, 11(9), e776-e782.  Yang, et al. (2005). Effect of structural change of collagen fibrils on the durability of dentin bonding. Biomaterials, 26(24), 5021-5031.  Yang, et al. (2012). Surface substance loss of subsurface bovine enamel lesions after different steps of the resinous infiltration technique: A 3D topography analysis. Odontology, 100(2), 172-180.  Yang, et al. (2022). A novel dental infiltration resin based on isosorbide-derived dimethacrylate with high biocompatibility, hydrolysis resistance, and antibacterial effect. Frontiers in Bioengineering and Biotechnology, 10.  Yap, et al. (2014). Evaluation of a novel approach in the prevention of white spot lesions around orthodontic brackets. Australian Dental Journal, 59(1), 70-80.  Yasuda, et al. (2008). Changes in elastic modulus of adhesive and adhesive-infiltrated dentin during storage in water. Journal of oral science, 50(4), 481-486.  Yasuda, et al. (2007). Determination of elastic modulus of demineralized resin-infiltrated dentin by self-etch adhesives. European Journal of Oral Sciences, 115(1), 87-91.  Yazioǧlu, et al. (2014). The investigation of non-invasive techniques for treating early approximal carious lesions: An in vivo study. International Dental Journal, 64(1), 1-11.  Yazkan, et al. (2018). Effect of resin infiltration and microabrasion on the microhardness, surface roughness and morphology of incipient carious lesions. Acta Odontologica Scandinavica, 76(7), 473-481.  Yeslam, et al. (2022). Time-dependent effect of intense capsule-coffee and bleaching on the color of resin-infiltrated enamel white spot lesions: an in vitro study. PeerJ, 10.  Yetkiner, et al. (2014). Colour improvement and stability of white spot lesions following infiltration, micro-abrasion, or fluoride treatments in vitro. European Journal of Orthodontics, 36(5), 595-602.  Yetkiner, et al. (2013). Caries infiltrant combined with conventional adhesives for sealing sound enamel in vitro. Angle Orthodontist, 83(5), 858-863.  Yetkiner, et al. (2014). Stability of two resin combinations used as sealants against toothbrush abrasion and acid challenge in vitro. Acta odontologica Scandinavica, 72(8), 825-830.  Yetkiner, et al. (2013). Effect of a low-viscosity adhesive resin on the adhesion of metal brackets to enamel etched with hydrochloric or phosphoric acid combined with conventional adhesives. Journal of Adhesive Dentistry, 15(6), 575-581.  Yim, et al. (2014). Modification of surface pre-treatment for resin infiltration to mask natural white spot lesions. Journal of Dentistry, 42(5), 588-594.  Yim, et al. (2014). Modification of surface pretreatment of white spot lesions to improve the safety and efficacy of resin infiltration. Korean Journal of Orthodontics, 44(4), 195-202.  Youssef, et al. (2020). Improving oral health: a short-term split-mouth randomized clinical trial revealing the superiority of resin infiltration over remineralization of white spot lesions. Quintessence international (Berlin, Germany : 1985), 51(9), 696-709.  Youssef, et al. (2022). Comparison of bleaching effects when applied to white-spot lesions before or after resin infiltration: An in vitro study. Journal of the American Dental Association, 153(1), 39-49.  Yu, et al. (2020). Anti-caries effect of resin infiltrant modified by quaternary ammonium monomers. Journal of Dentistry, 97.  Yuan, et al. (2013). Effect of infiltration technique and polishing on the roughness of artificial carious enamel surfaces. Shanghai kou qiang yi xue = Shanghai journal of stomatology, 22(4), 402-406.  Yuan, et al. (2014). Esthetic comparison of white-spot lesion treatment modalities using spectrometry and fluorescence. Angle Orthodontist, 84(2), 343-349.  Zago, et al. (2023). Evaluation of experimental resin infiltrant containing nanohydroxyapatite on color stability and microhardness in demineralized enamel. Clinical Oral Investigations, 27(11), 6835-6845.  Zakizade, et al. (2020). Effect of Resin Infiltration Technique on Improving Surface Hardness of Enamel Lesions: A Systematic Review and Meta-analysis. Journal of Evidence-Based Dental Practice, 20(2).  Zamorano Pino, et al. (2015). Microhardness of artificial white-spot lesions infiltrated with low viscosity resins before and after thermocycling. Avances en Odontoestomatologia, 31(6), 371-378.  Zawaideh, et al. (2011). Bonding of resin composite to caries-affected dentin after Carisolv® treatment. Pediatric Dentistry, 33(3), 213-220.  Zeng, et al. (2021). Real-time monitoring and quantitative evaluation of resin in-filtrant repairing enamel white spot lesions based on optical coherence tomography. Diagnostics, 11(11).  Zhang, et al. (2024). The Application of Resin Infiltration Combined with Nano-Hydroxyapatite in the Treatment of Post-Orthodontic White Spot Lesions. Journal of Biomedical Nanotechnology, 20(7), 1092-1098.  Zhao, et al. (2014). Effect of resin infiltration treatment on the colour of white spot lesions. Hua xi kou qiang yi xue za zhi = Huaxi kouqiang yixue zazhi = West China journal of stomatology, 32(3), 306-309.  Zhao, et al. (2014). Surface roughness and staining stability of infiltrant resin for enamel white spot lesion. Beijing da xue xue bao. Yi xue ban = Journal of Peking University. Health sciences, 46(1), 53-57.  Zhao, et al. (2016). Surface properties and color stability of resin-infiltrated enamel lesions. Operative Dentistry, 41(6), 617-626.  Zhong, et al. (2015). Contemporary research findings on dentine remineralization. Journal of Tissue Engineering and Regenerative Medicine, 9(9), 1004-1016.  Zhou, et al. (2017). Evaluation of resin infiltration on demineralized root surface: An in vitro study. Dental Materials Journal, 36(2), 195-204.  Zielińska, et al. (2016). The evaluation of caries lesion progression after infiltration with a low-viscous resin: In vivo study. Dental and Medical Problems, 53(3), 358-364.  Zlatarić, et al. (2016). Infiltration of the white spot demineralization lesions with icon system - A case report. Acta Stomatologica Croatica, 50(1), 95.  Zotti, et al. (2021). Resin Infiltration in Dental Fluorosis Treatment-1-Year Follow-Up. Medicina-Lithuania, 57(1), 14. |

**Supplementary Material 4b.** Reports assessed for eligibility; excluded with reasons, search date 2025-12-05.

| **Author. (Year). Title. Journal.** | **Reason for exclusion** |
| --- | --- |
| (2021). Caries prevention around the orthodontic appliance. https://trialsearch.who.int/Trial2.aspx?TrialID=RBR-2b2vvf5. | Trial registration |
| (2016). Esthetic improvements following resin infiltration and microabrasion of postorthodontic white-spot lesions in vivo. https://trialsearch.who.int/Trial2.aspx?TrialID=ChiCTR-IOR-16009908. | Doublet to published trial |
| (2020). A 24-Month Randomized Controlled Study on Treatment of Orthodontic Cretaceous Spots With Resin Infiltration, CPP-ACP and Sodium Fluoride Coating. https://trialsearch.who.int/Trial2.aspx?TrialID=ChiCTR2000032516. | Doublet to Published trial |
| (2022). Evaluation of the effects of fluoride varnish, CPP-ACP and infiltrating resin on enamel demineralization in orthodontic patients. https://trialsearch.who.int/Trial2.aspx?TrialID=ChiCTR2200056584. | Doublet to published trial |
| (2017). A clinical trial to study the effects of IconÂ® resin infiltration and Clinpro XT varnish on white spot lesions present post orthodontic treatment. https://trialsearch.who.int/Trial2.aspx?TrialID=CTRI/2017/11/010368. | Doublet to published trial |
| (2018). Comparing two different products i.e. fluoride varnish and resin infiltration for treatment of white spot lesion after orthodontic treatment. https://trialsearch.who.int/Trial2.aspx?TrialID=CTRI/2018/11/016414. | Trial registration |
| (2019). Evaluation of reduction in white spot lesions after three different treatment modalities. https:/trialsearch.who.int/Trial2.aspx?TrialID=CTRI/2019/09/021433. | Trial registration |
| (2019). To study the effects of commercially available product called ICON DMG, in management of white spots on permanent teeth. http://www.who.int/trialsearch/Trial2.aspx?TrialID=CTRI/2019/07/020115. | Trial registration |
| (2022). Infiltration of White-spot lesions during multibracket appliance treatment. https://trialsearch.who.int/Trial2.aspx?TrialID=DRKS00027344. | Trial registration |
| (2015). Effect of resin infiltration in color stability of white spot lesions in teeth. https://trialsearch.who.int/Trial2.aspx?TrialID=IRCT2014112720116N1. | Doublet to published trial |
| (2018). Effect of Resin Infiltration Technique on White Spot Lesions. https://trialsearch.who.int/Trial2.aspx?TrialID=IRCT20180624040223N1. | Doublet to published trial |
| (2023). The effects of ICON on early caries prevention in orthodontic patients. https://trialsearch.who.int/Trial2.aspx?TrialID=JPRN-UMIN000051407. | Trial registration |
| (2013). Randomized Trial on Resin Infiltration in Deciduous Teeth. https://clinicaltrials.gov/show/NCT01881100. | Trial registration |
| (2015). Enamel Damages Following De-bracketing From Infiltrated Surfaces. https://clinicaltrials.gov/show/NCT02359318. | Trial registration |
| (2016). One Year Clinical Evaluation of White Spot Lesions Treated With Newly Introduced Resin Modified Glass Ionomer in Comparison to Resin Infiltration in Anterior Teeth Split Mouth Technique. https://clinicaltrials.gov/show/NCT02912741. | Doublet to published trial |
| (2020). Performance of Different Resin Infiltration Materials on White Spot Lesions: clinical and Laboratory Assessments. https://clinicaltrials.gov/show/NCT04673097. | Trial registration |
| (2022). Clinical Evaluation of White Spot Lesions Treated by S-PRG Material and Composite Sealants in Comparison With ICON. https://clinicaltrials.gov/show/NCT05562856. | Trial registration |
| (2022). Efficacy of Different Agents in Treatment of Initial Caries on Smooth Surfaces in Permanent Teeth in Children. https://clinicaltrials.gov/ct2/show/NCT05206539. | Trial registration |
| N2023). Comparison of MI Paste Plus and Resin Infiltration in White Spot Lesions Following Orthodontic Treatment. https://clinicaltrials.gov/ct2/show/NCT05733676. | Trial registration |
| (2023). White Spot Lesions Treatment in Orthodontic. https://clinicaltrials.gov/ct2/show/NCT06051981. | Trial registration |
| Croll, et al. (2013). White spot lesions... [comment on] Senestraro SV, Crowe JJ, Wang M, et al. Minimally invasive resin infiltration of arrested white-spot lesions: a randomized clinical trial, JADA 2013;144[9]: 997-1005. Journal of the American Dental Association (1939), 144(12), 1332‐1334. | Commentary on without importance |
| Baafif, et al. (2020). The efficacy of resin infiltrant and casein phosphopeptide-amorphous calcium fluoride phosphate in treatment of white spot lesions (comparative study). Journal of International Society of Preventive and Community Dentistry, 10(4), 438-444. | Anterior tooth involvement not specified |
| Dhamija, et al. (2022). Efficacy of Resin Infiltration and Fluoride Casein Phosphopeptide Amorphous Calcium Phosphate Varnish on Non-cavitated Active White Spot Lesions in Children: A Randomized Clinical Trial. Pesquisa Brasileira em Odontopediatria e Clinica Integrada, 22. | No immediate post-treatment outcome |
| Feher, et al. (2016). The treatment of white spot lesions in pediatric dentistry-infltration with Icon®. Clujul Medical, 89, S57. | Conference abstract |
| Jumanca, et al. (2012). Infiltration Therapy - An Alternative to Fluoride Varnish Application for Treatment of White Spot Lesion after Fixed Orthodontic Treatment. Revista de Chimie, 63(8), 783-786. | Anterior tooth involvement not specified |
| Kannan, et al. (2019). Correction to: Comparative evaluation of Icon R resin infiltration and Clinpro TM XT varnish on colour and fluorescence changes of white spot lesions: a randomized controlled trial. Progress in Orthodontics, 20(1), 31. | A correction to without importance |
| Kraus, et al. (2014). Enamel demineralization in patients treated with fixed orthodontic appliances – Pre-evaluation of the effectiveness of resin infiltration treatment. Forum Ortodontyczne, 10(4), 246-254. | Anterior tooth involvement not specified |
| Leon, et al. (2019). Micro-invasive aesthetic treatment of non-cavitated white-spot lesions. Romanian Journal of Oral Rehabilitation, 11(1), 96-100. | Case report |
| Mazur, et al. (2018). Objective and subjective aesthetic performance of icon® treatment for enamel hypomineralization lesions in young adolescents: A retrospective single center study. Journal of Dentistry, 68, 104-108. | Follow-up < 3 months |
| Mazur, et al. (2022). In-vivo colour stability of enamel after ICON? treatment at 6 years of follow-up: A prospective single center study. Journal of Dentistry, 122, 6. | No immediate post-treatment outcome |
| Ogodescu, et al. (2011). [Resin infiltration of white spot lesions during the fixed orthodontic appliance therapy]. Revista medico-chirurgicalǎ̌ a Societǎ̌ţaii de Medici ş̧i Naturaliş̧ti din Iaş̧i, 115(4), 1251-1257. | No immediate post-treatment outcome |
| Rocha. (2021). Resolução estética de lesões de mancha branca pós-tratamento ortodôntico. https://doi.org/doi: | Follow-up < 3 months |
| Senestraro, et al. (2013). Minimally invasive resin infiltration of arrested white-spot lesions: A randomized clinical trial. Journal of the American Dental Association, 144(9), 997-1005. | Follow-up < 3 months |
| Sezici, et al. (2020). Low-viscosity resin infiltration efficacy on postorthodontic white spot lesions: A quantitative light-induced fluorescence evaluation. Turkish Journal of Orthodontics, 33(2), 92-97. | Follow-up < 3 months |
| Shan, et al. (2021). A comparison of resin infiltration and microabrasion for postorthodontic white spot lesion. American Journal of Orthodontics and Dentofacial Orthopedics, 160(4), 516-522. | No immediate post-treatment outcome |
| Souza. (2021). TRA associado ou não ao Brix3000, tendências mundiais e o conhecimento sobre técnicas de mínima intervenção para o controle da cárie dentária. https://doi.org/doi: | Not eligible diagnosis and intervention |
| Thirumoorthy, et al. (2024). Management of white spot enamel lesions with resin infiltration: potentials and future research directions. Evidence-Based Dentistry, 25(3), 160-161 | No clinical study |
| Thirumoorthy, et al. (2024). White spot enamel lesions – is treatment or prevention the answer to this ‘no win scenario’? Evidence-Based Dentistry, 25(1), 51-52. | No clinical study |
| Turska-Szybkaa, et al. (2016). Randomised clinical trial on resin infiltration and fluoride varnish vs fluoride varnish treatment only of smooth-surface early caries lesions in deciduous teeth. Oral Health and Preventive Dentistry, 14(6), 485-491. | No eligible intervention |
| Wang, et al. (2013). Efficiency of resin infiltration versus fluoride varnish for treatment of post-orthodontic white spot lesions. Chinese Journal of Tissue Engineering Research, 17(29), 5303-5308. | No immediate post-treatment outcome |
| Wang, et al. (2023). Comparative evaluation of four treatments for postorthodontic white spot lesions: a randomized controlled trial. Clinical Oral Investigations, 27(10), 5957-5968. | No immediate post-treatment outcome |

**Supplementary Material 4c.** Reports assessed for eligibility; included, search date 2025-12-05

| **Author. (Year). Title. Journal.** |
| --- |
| Ciftci, et al. (2018). The efficacy of resin infiltrate on the treatment of white spot lesions and developmental opacities. Nigerian Journal of Clinical Practice, 21(11), 1444-1449. |
| Ding, et al. (2024). In-vitro and in-vivo comparative studies of treatment effects on enamel demineralization during orthodontic therapy: implications for clinical early-intervention strategy. Clinical Oral Investigations, 28(10). |
| Eckstein, et al. (2015). Camouflage effects following resin infiltration of postorthodontic white-spot lesions in vivo: One-year follow-up. Angle Orthodontist, 85(3), 374-380. |
| ElSayed, et al. (2021). Resin Infiltration Versus Acid Micro-Abrasion In The Treatment Of White Spot Lesions In Fixed Orthodontic Patients. Future, 7(2), 90-94. |
| Feng, et al. (2013). [Efficacy of one year treatment of icon infiltration resin on post-orthodontic white spots]. Beijing da xue xue bao. Yi xue ban = Journal of Peking University. Health sciences, 45(1), 40-43. |
| Gabr, et al. (2024). Comparative evaluation of Giomer coating material Versus Icon resin infiltration in masking of White Spot Lesions (An In Vivo Study). Al-Azhar Journal of Dental Science, 27(3), 331-337. |
| Gabr, et al. (2022). Clinical Evaluation of White Spot Lesions Treated by S-PRG and ICON. https://clinicaltrials.gov/show/NCT05550116. |
| Gholami, et al. (2023). Effect of Extent of White Spot Lesions on the Esthetic Outcome After Treatment by the Resin Infiltration Technique: A Clinical Trial. Frontiers in Dentistry, 20. |
| Giannetti, et al. (2018). Superficial infiltration to treat white hypomineralized defects of enamel: Clinical trial with 12-month follow-up. Journal of Biological Regulators and Homeostatic Agents, 32(5), 1335-1338. |
| Giray, et al. (2018). Resin infiltration technique and fluoride varnish on white spot lesions in children: Preliminary findings of a randomized clinical trial. Nigerian Journal of Clinical Practice, 21(12), 1564-1569. |
| Giudice, et al. (2020). Spectrophotometric evaluation of enamel color variation using infiltration resin treatment of white spot lesions at one year follow-up. Dentistry Journal, 8(2). |
| Goda, et al. (2018). An in vivo study on the durability of resin infiltration technique on color masking of white spot lesions. Al-Azhar Journal of Dental Science, 21(2), 121-126. |
| Gu, et al. (2019). Esthetic improvements of postorthodontic white-spot lesions treated with resin infiltration and microabrasion: A split-mouth, randomized clinical trial. Angle Orthodontist, 89(3), 372-377. |
| Gözetici, et al. (2019). Comparative evaluation of resin infiltration and remineralisation of noncavitated smooth surface caries lesions: 6-month results. Oral Health and Preventive Dentistry, 17(2), 99-106. |
| Kabaktchieva, et al. (2014). The role of light-induced fluorescence in the treatment of smooth surface carious lesions with icon infiltration and the results after 1 year. Acta Medica Bulgarica, 41(2), 36-42. |
| Kannan, et al. (2019). Comparative evaluation of Icon® resin infiltration and Clinpro™ XT varnish on colour and fluorescence changes of white spot lesions: a randomized controlled trial. Progress in Orthodontics, 20(1). |
| Kashash, et al. (2024). Resin infiltration versus fluoride varnish for visual improvement of white spot lesions during multibracket treatment. A randomized-controlled clinical trial. Clinical Oral Investigations, 28(6). |
| Knaup, et al. (2023). Correlation of quantitative light-induced fluorescence and qualitative visual rating in infiltrated post-orthodontic white spot lesions. European Journal of Orthodontics, 45(2), 133-141. |
| Knösel, et al. (2013). Durability of esthetic improvement following Icon resin infiltration of multibracket-induced white spot lesions compared with no therapy over 6 months: A single-center, split-mouth, randomized clinical trial. American Journal of Orthodontics and Dentofacial Orthopedics, 144(1), 86-96. |
| Knösel, et al. (2019). Long-term follow-up of camouflage effects following resin infiltration of post orthodontic white-spot lesions in vivo. Angle Orthodontist, 89(1), 33-39. |
| Krasniqi, et al. (2024). Comparative Evaluation of Resin Infiltration and Bifluoride Varnish in White Spots in Children between the Ages of 8 and 15 Years. International Journal of Biomedicine, 14(2), 329-334. |
| Krikheli, et al. (2020). Comparison of resin infiltration and microabrasion in the treatment of enamel caries. Clinical Dentistry (Russia)(3), 10-13. |
| Ozgur, et al. (2023). Effectiveness and Color Stability of Resin Infiltration on Demineralized and Hypomineralized (MIH) Enamel in Children: Six-month Results of a Prospective Trial. Operative dentistry, 48(3), 258-267. |
| Puleio, et al. (2023). Long-Term Chromatic Durability of White Spot Lesions through Employment of Infiltration Resin Treatment. Medicina (Lithuania), 59(4). |
| Rashid, et al. (2018). The effect of resin infiltration on the color of white spot lesions after ort hodontic treatment over one year period (in vivo study). Egyptian Dental Journal, 64(4-October (Orthodontics, Pediatric & Preventive Dentistry)), 3069-3073. |
| Rohym, et al. (2021). One year clinical evaluation of white spot lesions with newly introduced resin modified glass-ionomer in comparison to resin infiltration in anterior teeth: A split mouth randomized controlled clinical trial from egypt. Brazilian Dental Science, 24(1), 1-13. |
| Simon, et al. (2022). Management of Post Orthodontic White Spot Lesions Using Resin Infiltration and CPP-ACP Materials- A Clinical Study. Journal of Clinical Pediatric Dentistry, 46(1), 70-73. |
| Tinghong, et al. (2019). Infiltration resin versus microabrasion technique for repairing white spot lesions after orthodontics. Chinese Journal of Tissue Engineering Research, 23(22), 3522-3529. |
| Wierichs, et al. (2023). Evaluation of the masking efficacy of caries infiltration in post-orthodontic initial caries lesions: 1-year follow-up. Clinical Oral Investigations, 27(5), 1945-1952. |
| Wierichs, et al. (2023). Aesthetic caries infiltration – Long-term masking efficacy after 6 years. Journal of Dentistry, 132. |
| Wierichs, et al. (2023). Masking-efficacy and caries arrestment after resin infiltration or fluoridation of initial caries lesions in adolescents during orthodontic treatment–A randomised controlled trial. Journal of Dentistry, 138. |
| Zaazou, et al. (2024). Effectiveness of low-viscosity resin infiltration (Icon) on color change of enamel white spot lesions: 1-year follow-up clinical study. Bulletin of the National Research Centre, 48(1), 62. |

**Supplementary Material 4d.** Reports identified from the updated literature search with their inclusion/exclusion status, search date 2025-09-29

| **Author. (Year). Title. Journal.** | **Inclusion/exclusion status** |
| --- | --- |
| Evaluation of Resin Infiltrate As Pit and Fissure Sealant in Permanent Molars (In Vivo& in Vitro). (2024). https://doi.org/doi:  Ahmed, et al. (2025). Spectrophotometry Comparison and Microleakage Analysis of Bioactive Glass-Reinforced Resin Infiltrants: An In Vitro Study. European Journal of General Dentistry.  Ahmed, et al. (2025). In-vitro comparative thermo-chemical aging and penetration analyses of bioactive glass-based dental resin infiltrates. PeerJ, 13(1).  Ahmed, et al. (2024). Physicochemical assessments of ion-doped bioactive-glass-based resin infiltrants. Bioinspired, Biomimetic and Nanobiomaterials, 13(3), 45-57.  Albar, et al. (2024). Color Masking Ability of Guided Enamel Regeneration with a Novel Self-Assembling Peptide and Resin Infiltration on Artificial Enamel Lesions Under Various Challenges: An In Vitro Spectrophotometric Analysis. Biomimetics, 9(12).  Almosa, et al. (2024). Assessment of Change in Enamel Color and Surface Hardness Following the Use of ICON Resin Infiltration and Remineralizing Agent: An In Vitro Study. Materials, 17(24).  Alqahtani, et al. (2025). Advancements in Minimally Invasive Techniques in Pediatric Dentistry: A Review. Cureus, 17(1), e76929.  Alshahrani, et al. (2024). Effect of Bleaching on Surface Roughness of White Spot Lesions Treated with Different Modalities of Resin Infiltration: An Vitro Study. Journal of Pharmacy and Bioallied Sciences, 16, S3871-S3873.  Alwadai, et al. (2025). Multi-modal management of dental fluorosis in a family A case series. Medicine (United States), 104(15), e42082.  Baccolini, et al. (2025). The Role of Casein Phosphopeptide-Amorphous Calcium Phosphate (CPP-ACP) in White Spot Lesion Remineralization-A Systematic Review. Journal of Functional Biomaterials, 16(8), 13.  Bakry, et al. (2025). In-vitro comparative study for three different strategies to treat enamel demineralized white spot lesion. Journal of Dentistry, 156.  Barrionuevo, et al. (2024). Determination of enamel microhardness in white spot lesions treated with infiltrating resins. Revista de la Asociacion Odontologica Argentina, 112(2).  Bjelović, et al. (2025). Minimally invasive treatment for esthetic enhancement of enamel hypoplasia in upper central incisors: clinical case reports. Experimental and Applied Biomedical Research (EABR).  Borelli Neto, et al. (2025). Does the Transillumination Technique Using a Diagnostic White Tip Influence the Degree of Conversion of the Infiltrant Resin? A Case Report With In Vitro Insights : A New Technique to Improve the Predictability During the Application of Resin Infiltration: Transillumination Using a Diagnostic Whi. Journal of Esthetic and Restorative Dentistry, 37(8), 1985-1993.  Cai, et al. (2025). Plasma-assisted extraction of high-purity chitin from crab shells: Eliminating the need for chemical decolorization. Carbohydrate Polymers, 369.  Camargo, et al. (2025). Resin infiltration for the esthetic treatment of molar-incisor hypomineralization: 1-year follow-up. Quintessence International, 56(6), 472-479.  Carneiro, et al. (2025). Resin infiltration and in-office dental bleaching on different substrates: hydrogen peroxide penetration, color change, and enamel morphology- an in vitro study. Clinical Oral Investigations, 29(10), 10.  Cheng. (2024). Resin infiltration may help mask enamel white spot lesions or fluorosis. Journal of Evidence-Based Dental Practice, 24(4).  Dallavilla, et al. (2025). Early enamel affected lesions: benefits and limitations of simplified resin techniques. Brazilian Dental Science, 28(2).  de Oliveira Iatarola, et al. (2025). Modified microabrasion protocol associated with resin infiltration Two case reports. International Journal of Esthetic Dentistry, 20(3), 268-283.  Eden. (2025). *Evidence-based caries prevention: Second edition*. Springer. https://doi.org/doi:10.1007/978-3-031-79152-9  El-Embaby, et al. (2024). Impact of silica nanoparticles incorporation on the properties of resin infiltration: an in vitro study. BMC Oral Health, 24(1).  Farghal, et al. (2025). Evaluation of icon resin infiltration and CPP-ACP alone or with Er: YAG laser (2940 nm) on surface morphology and hardness of enamel white spot lesions (in vitro study). Lasers in Dental Science, 9(1).  Feng, et al. (2025). The effect of novel solvent formula of primer containing functional monomer on improving the durability of dentin adhesion. Journal of Dentistry, 161.  Haro Párraga, et al. (2024). Infiltrating resin in orthodontic white spot lesions in patients at the San Gregorio de Portoviejo University. Salud, Ciencia y Tecnologia, 4.  Hasan, et al. (2025). A novel radiopaque resin infiltrant for managing early enamel caries. Journal of Dentistry, 161.  Hilgert, et al. (2025). Resin infiltration: A microinvasive treatment for carious and hypomineralised enamel lesions. Springer. https://doi.org/doi:10.1007/978-3-031-79152-9_8  Inna, et al. (2024). The Staining Susceptibility and Surface Roughness of Teeth Restored by Microabrasion and Resin Infiltration: An In Vitro Study. Polymers, 16(24).  Jazam, et al. (2025). Comparative Evaluation of Enamel Surface Roughness after Minimally Invasive Treatment of White Spot Lesions: An In-vitro Experimental Study. Journal of Clinical and Diagnostic Research, 19(5), ZC28-ZC33.  Khan, et al. (2025). Effect of resin infiltration system and pit-fissure sealant on white spot lesion: An in vitro study. Bioinformation, 21(5), 1169-1175.  Kiryk, et al. (2025). The Influence of Resin Infiltration on the Shear Bond Strength of Orthodontic Brackets: A Systematic Review and Meta-Analysis. Journal of Functional Biomaterials, 16(1).  Lamorgese, et al. (2025). White Spot Lesion Treatment Options: A Systematic Review of Different Techniques for Masking These Lesions. Gels, 11(5).  Landmayer, et al. (2025). Effect of EGCG-Methacrylate-Functionalized Resin Infiltrant on White Spot Lesions: An In Vitro Study. Journal of Functional Biomaterials, 16(1), 15.  Lferde, et al. (2025). Aesthetic Management of Molar-Incisor Hypomineralization With Deep Resin Infiltration: A Case Report. Cureus, 17(6), e85800.  Maksymiuk, et al. (2025). Experimental laser fluorescence characteristics of initial caries lesions after resin infiltrant application. Journal of Dentistry, 162.  Manav Özen, et al. (2025). Evaluation of NaOCl application prior to resin infiltrant or fluoride-containing resin varnish in the treatment of white spot lesions: An in vitro study. Journal of Dentistry, 156.  Manna, et al. (2025). Etching on the edge: enamel loss under repeated and active HCl applications as a resin infiltration pretreatment. Journal of Applied Oral Science, 33, e20250103.  Marouane, et al. (2025). In vivo comparison of resin infiltration outcomes under different light conditions: A randomized controlled clinical trial. Journal of Dentistry, 153, 9.  Nct. (2024). Resin Infiltrate As Pit and Fissure Sealant in Permanent Molars. https://clinicaltrials.gov/ct2/show/NCT06729788.  Nct. (2025). "Comparison of the Effects of Resin Infiltrant, Fluoride Varnish, and the Combined Application of Resin Infiltrant and Fluoride Varnish on Primary Molars in the Treatment of Proximal Non-Cavitated Carious Lesions in Children.". https://clinicaltrials.gov/ct2/show/NCT07112963.  Ni, et al. (2024). Effect of sodium hypochlorite and EDTA pretreatment on the resin infiltration efficacy and acid resistance of enamel white spot lesions: an in vitro study. BMC Oral Health, 24(1).  Novozhilova, et al. (2025). Color Change and Color Stability of White Spot Lesions Treated with Resin Infiltration, Microabrasion, or Nano-Hydroxyapatite Remineralization: An In Vitro Study. Dentistry Journal, 13(3).  Pattamalai, et al. (2024). Effects of Enamel Pretreatment Methods and Timing of Applications on Orthodontic Bond Strength. Journal of International Dental and Medical Research, 17(4), 1393-1400.  Prada, et al. (2024). A Review of White Spot Lesions: Development and Treatment with Resin Infiltration. Dentistry Journal, 12(12).  Prado, et al. (2025). Does the application protocol influence the masking effect of resin infiltration on MIH opacities? Systematic review and meta-analysis. Journal of Dentistry, 155, 14.  Ramos, et al. (2025). What are the global trends in research on resin infiltration in dentistry? An altmetric and bibliometric analysis. Brazilian Oral Research, 39.  Salah, et al. (2025). In vitro evaluation of shear bond strength of ceramic bonded to remineralized enamel. Tanta Dental Journal, 22(1), 63-70.  Schoppmeier, et al. (2025). Influence of home or in-office tooth bleaching on the color stability of white-spot lesions after resin infiltration: An in vitro comparison. Journal of the American Dental Association, 156(7), 517-529.e513.  Shi, et al. (2025). Application of quantitative light-induced fluorescence technology in early caries detection and resin infiltration treatment. Journal of Dentistry, 162.  Singh, et al. (2024). Scale dependent nanomechanical properties of dentin adhesive and adhesive-collagen composite. Frontiers in Dental Medicine, 5, 9.  Souza, et al. (2024). Resin infiltrant with antibacterial activity: effects of incorporation of DMAHDM monomer and NACP on physical and antimicrobial properties. Journal of Applied Oral Science, 32, m.  Sravya, et al. (2025). Effectiveness of ei-nRsnIfilrtoitain in Masking White Spot Lesions and Enamel Defects, Color Stability and Inhibition of Caries Progression: A Systematic Review. Journal of Dentistry Indonesia, 32(2), 10.  Tahmasbi, et al. (2024). Surface Microhardness, Masking, and Color Stability of white Spot Lesions Infiltrated by Icon versus an Experimental Resin Containing MA-POSS Nanoparticles. Journal of Research in Dental and Maxillofacial Sciences, 9(4), 297-304.  Todorova, et al. (2025). One- and Two-Year Efficacy of Resin Infiltration and Remineralization for the Treatment of Initial Proximal Caries. Journal of Functional Biomaterials, 16(7).  Torres, et al. (2024). Optimizing Resin Infiltration Procedure in Molar Incisor Hypomineralization Lesions. J Esthet Restor Dent.  Ulrich, et al. (2025). An ex vivo study on the correlation between lesion size and resin infiltration area in natural proximal subsurface carious lesions deproteinized with sodium hypochlorite. Journal of Dentistry, 160, 11.  Wang, et al. (2025). Fabricating a novel bioactive resin Infiltrant to treat white spot lesions of enamel. Journal of Dentistry, 158.  Xia, et al. (2025). Expert consensus on the prevention and treatment of enamel demineralization in orthodontic treatment. International Journal of Oral Science, 17(1), 11.  Yerkibayeva, et al. (2025). Non-invasive esthetic treatment of initial caries with resin infiltration in a patient with autism spectrum disorder. Georgian Medical News, 361(4), 121-126. | Excluded by title and/or abstract |
| Clinical Outcome of Resin Infiltration Vs Self-Assembling Peptide (P11-4) in Treating White Spot Lesion - a Randomized Clinical Trial. (2024). https://doi.org/doi: | Excluded; Trial registration |
| The Color Masking Potentials of a Bioactive Glass Adhesive and the Resin ICON on the White Spot Lesions. A Comparative Clinical Trial. (2024). https://doi.org/doi: | Excluded; Doublet to published trial |
| Aesthetic Efficacy of Resin Infiltration Using Different Conditioning Methods: 24-Month Results. (2024). https://doi.org/doi: | Excluded; Trial registration |
| Clinical and Laboratory Evaluation of Icon® Resin Infiltration and Vanish™ XT Varnish, on Color Changes of White Spot Lesions. (2024). https://doi.org/doi: | Excluded; Trial registration |
| Albashaireh, et al. (2025). Comparative evaluation of ICON resin infiltration and bioactive glass adhesive for managing initial caries lesions using quantitative light-induced fluorescence: a randomized clinical trial. Journal of Dentistry, 159. | Excluded; No immediate post-treatment outcome |
| Ctri. (2025). To compare minimally invasive approaches of Self assembling peptide (SAP) with Resin infiltration (RI) for management for initial smooth surface carious lesions. https://trialsearch.who.int/Trial2.aspx?TrialID=CTRI/2025/04/085123. | Excluded; Trial registration |
| Elrashid, et al. (2025). Comparing the Aesthetic Outcomes of Orthodontically Induced White Spot Lesion Using Icon R Resin Infiltration, Fluoride Varnish and Micro-Abrasion: A Pilot Study. Journal of Pharmacy and Bioallied Sciences., 17, S814-S818. | Excluded; Follow-up < 3 months |
| Golia, et al. (2025). Comparative non-invasive strategies for managing dental white spot lesions: Contribution to the study of enamel remineralization. Acta Marisiensis - Seria Medica, 71(2), 147-152. | Included |
| Wakwak, et al. (2025). Comparative evaluation of different resinous infiltrants for masking efficacy of non-cavitated enamel lesions: a one-year randomized controlled trial. Journal of Stomatology, 78(1), 32-41. | Included |

**Supplementary Material 5.** Author communications.

| **Study**  **(Author year)** | **Purpose of contact** | **Date of first attempt (Year-month)** | **Response received** | **Summary of response** | **Follow-up attempts** |
| --- | --- | --- | --- | --- | --- |
| Baafif 2020 | Localization of WSL and orthodontic history | 2025-01 | Partially | Author intended to check data; no further reply | 2 additional attempts (no reply) |
| Ding 2024 | Localization of WSL | 2025-01 | Yes | Group C (RI): 10 patients, 50 tooth surfaces (27 anterior, 15 premolars, 8 molars) |  |
| Knösel 2013; Eckstein 2015; Knösel 2019 | Localization of WSL | 2025-01 | Yes | Treated teeth included both jaws, incisors, and canines |  |
| Giannetti 2018 | Number of patients | 2025-01 | No | - | 2 additional attempts (no reply) |
| Giudice 2020 | Localization of WSL | 2025-01 | Yes | Included all teeth with WSLs in both jaws |  |
| Jumanca 2012 | Localization of WSL | 2025-01 | No | - | 2 additional attempts (no reply) |
| Kraus 2013 | Localization of WSL | 2025-01 | No | - | 2 additional attempts (no reply) |
| Puleio 2023 | Localization of WSL and number of patients | 2025-01 | Yes | 40 patients; teeth randomly distributed (anterior/posterior; maxillary/mandibular) |  |
| Simon 2022 | Number of WSLs treated with RI | 2025-03 | Yes | One tooth from each child was treated |  |
| Wierichs 2023 | Clarification of follow-up period | 2025-09 | Yes | Confirmed follow-up = 6 months |  |
| Gabr 2024; Wakwak 2025 | Clarification of results and shared sample | 2025-11 | Yes | Provided clarification; confirmed same sample |  |

RI, resin infiltration; WSL, white spot lesion.

**Supplementary Material 6.** Studies excluded from quantitative synthesis and reasons for exclusion.

| **Study (Author year)** | **Reason for Exclusion** |
| --- | --- |
| Ding 2024 | Missing data |
| Giannetti 2018 | No data available |
| Gholami 2023 | White spot lesion analyzed together with other enamel opacities |
| Goda 2018 | Missing data |
| Golia 2025 | Missing data |
| Kabaktchieva 2014 | No data available |
| Krikheli 2020 | No relevant outcome reported |

**Supplementary Material 7.** Additional experimental interventions (apart from resin infiltration) tested in the included studies and omitted from this review. Empty rows indicate that the study did not include a comparator.

| Study  (Author year) | Type of intervention for WSL  Patients (teeth) |
| --- | --- |
| Çiftçi 2018 | FV: 18 (40) |
| Ding 2024 | FV: 11 (53)  CPP-ACP: 12 (60) |
| Elsayed 2021 | MA: 12 (45) |
| Feng 2013 | - |
| Gabr 2022 | SPRG: 20 (20) |
| Gabr 2024; Wakwak 2025 | SPRG: 20 (20), CRS: 20 (40) |
| Gholami 2023 | - |
| Giannetti 2018 | - |
| Giray 2018 | FV: 11 (36) |
| Giudice 2020 | - |
| Goda 2018 | DA: 20 (60) |
| Golia 2025 | FV: 24 (NR) |
| Gu 2019 | MA: 20 (64) |
| Gözetici 2019 | SAP P11-14: 21 (21)  FV: 21 (21) |
| Kabaktchieva 2014 | - |
| Kannan 2019 | FRC: 6 (91) |
| Kashash 2024 | FV: 19 (64) |
| Knaup 2023; Wierichs 2023a,b | - |
| Knösel 2013; Eckstein 2015; Knösel 2019 | - |
| Krasniqi 2024 | FV: 30 (88) |
| Krikheli 2020 | MA: 40 (NR) |
| Ozgur 2023 | - |
| Puleio 2023 | - |
| Rashid 2018 | - |
| Rohym 2021 | FRC: 6 (36) |
| Simon 2022 | CCP-ACP: 30 (30) |
| Tinghong 2019 | MA: 11 (44) |
| Wierichs 2023 | FV: 17(38) |
| Zaazou 2024 | - |

CPP-ACP, casein phosphopeptide–amorphous calcium phosphate; CRS, composite resin sealants; DA, dental adhesive; FRC, fluoride releasing coating; FV, fluoride varnish; MA, micro abrasion; SAP P11-14, self-assembling peptides P11–14; SPRG, surface pre-reacted glass-ionomer

**Supplementary Material 8.** Risk of bias of the included studies; modified JBI checklist for cohort studies.

| **Nr** | **Author year** | **Sample size sufficient ≥80 teeth** | **RI protocol described** | **Protocol similar for all WSLs** | **Exclusion and inclusion criteria described** | **Analysis of confounding factors** | **Outcomes measured in a reliable way** | **Outcome assessors blinded** | **Same measurement at different time points** | **Sufficient follow-up time** | **Difference in characteristics between time points** | **Loss of follow-up of concern** | **Reasons of loss at follow-up described** | **Strategies to address incomplete follow-up** | **Appropriate statistical analysis** | **Clustering considered** | **Conflict of interest** |
| --- | --- | --- | --- | --- | --- | --- | --- | --- | --- | --- | --- | --- | --- | --- | --- | --- | --- |
| 1 | Çiftçi 2018 | No | Yes | Yes | Yes | No | Yes | Yes | Yes | No | No | No | NA | NA | Yes | No | No |
| 2 | Ding 2024 | No | No | NR | Yes | No | Yes | No | Yes | No | No | No | NA | NA | Yes | No | No |
| 3 | Elsayed 2021 | No | Yes | Yes | Yes | No | Yes | No | Yes | No | No | No | NA | NA | Yes | No | NR |
| 4 | Feng 2013 | No | Yes | Yes | Yes | No | Yes | Yes | Yes | Yes | No | No | NA | NA | Yes | No | No |
| 5 | Gabr 2022 | No | Yes | Yes | Yes | No | Yes | Yes | Yes | Yes | NR | Yes | Yes | No | Yes | Yes | No |
| 6 | Gabr 2024; Wakwak 2025 | No | Yes | Yes | Yes | No | Yes | Yes | Yes | Yes | NR | Yes | No | No | Yes | Yes | No |
| 7 | Gholami 2023 | No | Yes | Yes | Yes | Partially | Yes | No | Yes | No | No | No | NA | NA | Yes | No | No |
| 8 | Giannetti 2018 | No | Yes | No | No | No | Yes | No | Yes | Yes | No | No | NA | NA | No | No | No |
| 9 | Giray 2018 | No | Yes | Yes | Yes | No | Yes | Yes | Yes | No | No | No | NA | NA | Yes | No | No |
| 10 | Giudice 2020 | No | Yes | No | Yes | Partially | Yes | No | Yes | Yes | No | No | NA | NA | Yes | Yes | No |
| 11 | Goda 2018 | No | Yes | Yes | Yes | No | Yes | No | Yes | Yes | No | No | NA | NA | Yes | No | NR |
| 12 | Golia 2025 | No | Yes | NR | Yes | No | No | No | Yes | No | No | No | NA | NA | Yes | No | No |
| 13 | Gözetici 2019 | No | Yes | Yes | Yes | No | Yes | Yes | Yes | No | NR | No | NA | NA | Yes | Yes | No |
| 14 | Gu 2019 | No | Yes | No | Yes | No | Yes | No | Yes | Yes | NR | Yes | No | No | Yes | No | NR |
| 15 | Kabaktchieva 2014 | No | Yes | Yes | No | No | Yes | No | Yes | Yes | No | No | NA | NA | No | No | NR |
| 16 | Kannan 2019 | Yes | Yes | Yes | Yes | No | Yes | No | Yes | No | No | No | NA | NA | Yes | No | No |
| 17 | Kashash 2024 | No | Yes | No | Yes | No | Yes | Yes | Yes | No | NR | No | NA | NA | Yes | No | No |
| 18 | Knaup 2023; Wierichs 2023a,b | Yes | Yes | No | Yes | Partially | Yes | Yes | Yes | Yes | NR | Yes | Partially | Partially | Yes | No | Yes |
| 19 | Knösel 2013; Eckstein 2015; Knösel 2019 | Yes | Yes | No | Yes | No | Yes | No | Yes | Yes | NR | Yes | Partially | No | Yes | Yes* | Yes |
| 20 | Krasniqi 2024 | Yes | Yes | Yes | Yes | No | Yes | No | Yes | No | No | No | NA | NA | Yes | No | No |
| 21 | Krikheli 2020 | NR | Yes | Yes | Yes | No | No | No | Yes | Yes | No | No | NA | NA | Yes | No | NR |
| 22 | Ozgur 2023 | No | Yes | Yes | Yes | No | Yes | Yes | Yes | No | NR | No | NA | NA | Yes | No | No |
| 23 | Puleio 2023 | No | Yes | No | Yes | No | Yes | Yes | Yes | Yes | No | No | NA | NA | Yes | Yes | No |
| 24 | Rashid 2018 | NR | Yes | Yes | Partially | No | Partially | No | Yes | Yes | No | No | NA | NA | Yes | No | NR |
| 25 | Rohym 2021 | No | Yes | Yes | No | No | Yes | No | Yes | Yes | No | No | NA | NA | Yes | No | No |
| 26 | Simon 2022 | No | No | NR | Yes | No | Yes | Yes | Yes | Yes | No | No | NA | NA | Yes | Yes | NR |
| 27 | Tinghong 2019 | No | Yes | Yes | Yes | No | Yes | Yes | Yes | Yes | No | No | NA | NA | Yes | No | No |
| 28 | Wierichs, 2023 | No | Yes | No | Yes | No | Yes | Yes | Yes | No | NR | No | NA | NA | Yes | No | Yes |
| 39 | Zaazou 2024 | Yes | Yes | No | Yes | No | Yes | No | Yes | Yes | No | No | NA | NA | Yes | No | NR |

NA, not applicable; NR, not reported; RI, resin infiltration; WSL, white spot lesion.

*clustering accounted for only in Knösel 2013

**Supplementary Material 9.** Outcomes assessed by single studies.

| **Nr** | **Outcome** | **Time period** | **Change (95% CI)** | **P** | **Statistically significant** |
| --- | --- | --- | --- | --- | --- |
| 1 | L_WSL_ | Post-1 mo | -0.30 (-0.64, 0.04) | 0.08 | **No** |
| 2 | ICDAS II | Post-1 mo | -0.02 (-0.14, 0.10) | 0.74 | **No** |
| 3 | Tx efficiency | Post-1 mo | 13.77 (9.57, 17.97) | <0.001 | **Yes** |
| 4 | WSL area | Post-1 mo | -1.60 (-2.50, -0.70) | 0.001 | **Yes** |
| 5 | WSL area ratio | Post-1 mo | -5.34 (-7.87, -2.82) | <0.001 | **Yes** |
| 6 | a_WSL_ | Post-3 mos | -0.08 (-0.24, 0.08) | 0.34 | **No** |
| 7 | b_WSL_ | Post-3 mos | -0.06 (-0.92, 0.80) | 0.89 | **No** |
| 8 | Q_WSL_ | Post-3 mos | -0.78 (-0.96, -0.60) | <0.001 | **Yes** |
| 9 | Tx efficiency | Post-3 mos | 18.34 (14.15, 22.53) | <0.001 | **Yes** |
| 10 | WSL area | Post-3 mos | -2.44 (-3.39, -1.49) | <0.001 | **Yes** |
| 11 | WSL area ratio | Post-3 mos | -7.11 (-9.96, -4.43) | <0.001 | **Yes** |
| 12 | L_WSL/SAE_ | Post-6 mos | 0 (-0.58, 0.58) | 1.00 | **No** |
| 13 | a_WSL/SAE_ | Post-6 mos | 0.13 (-0.43, 0.69) | 0.65 | **No** |
| 14 | b_WSL/SAE_ | Post-6 mos | 0.26 (-0.41, 0.93) | 0.45 | **No** |
| 15 | Q_WSL_ | Post-6 mos | -1.36 (-1.55, -1.17) | <0.001 | **Yes** |
| 16 | Grey value | Post-6 mos | -6.87 (-8.20, -5.54) | <0.001 | **Yes** |
| 17 | Tx efficiency | Post-6 mos | 17.80 (13.61, 21.99) | <0.001 | **Yes** |
| 18 | WSL area | Post-6 mos | -2.58 (-3.53, -1.63) | <0.001 | **Yes** |
| 19 | L_WSL_ | Post-12 mos | 1.30 (0.92, 1.68) | <0.001 | **Yes** |
| 20 | L_WSL/SAE_ | Post-12 mos | 0.85 (-0.60, 2.30) | 0.25 | **No** |
| 21 | F_WSL_ | Post-12 mos | -0.07 (-0.19, 0.05) | 0.26 | **No** |
| 22 | F_WSL/SAE_ | Post-12 mos | -0.30 (-0.52, -0.08) | 0.006 | **Yes** |
| 23 | Q_WSL_ | Post-12 mos | 0.06 (-0.06, 0.18) | 0.32 | **No** |
| 24 | Q_WSL/SAE_ | Post-12 mos | -0.50 (-1.01, 0.01) | 0.05 | **No** |
| 25 | Grey value | Post-12 mos | -3.63 (-5.02, -2.24) | <0.001 | **Yes** |
| 26 | Tx efficiency | Post-12 mos | 17.59 (13.40, 21.78) | <0.001 | **Yes** |
| 27 | ICDAS II | Post-12 mos | -0.18 (-0.31, -0.06) | 0.005 | **Yes** |
| 28 | Visual score | Post-12 mos | -0.20 (-0.36, -0.05) | 0.01 | **Yes** |
| 29 | DE_WSL/SAE_ | Post-48 mos | 0.22 (-0.22, 0.66) | 0.33 | **No** |
| 30 | WSL area | Post-72 mos | 0.05 (0, 0.10) | 0.04 | **Yes** |
| 31 | DE_WSL/SAE_ | Post-72 mos | -0.80 (-1.23, -0.37) | <0.001 | **Yes** |
| 32 | F_WSL/SAE_ | Post-72 mos | -0.30 (-0.52, -0.08) | 0.006 | **Yes** |
| 33 | Q_WSL/SAE_ | Post-72 mos | -0.50 (-1.01, 0.01) | 0.05 | **No** |
| 34 | ICDAS II | Post-72 mos | 0.08 (-0.06, 0.22) | 0.25 | **No** |
| 35 | Visual score | Post-72 mos | -0.20 (-0.36, -0.05) | 0.01 | **Yes** |
| 36 | L_WSL_ | 1 mo-3 mos | 0.40 (0.20, 0.60) | <0.001 | **Yes** |
| 37 | ICDAS II | 1 mo-3 mos | 0 (-0.11, 0.11) | 1.00 | **No** |
| 38 | Tx efficiency | 1 mo-3 mos | 4.57 (1.35, 7.79) | 0.005 | **Yes** |
| 39 | WSL area | 1 mo-3 mos | -0.84 (-1.56, -0.13) | 0.02 | **Yes** |
| 40 | WSL area ratio | 1 mo-3 mos | -1.77 (-3.05, -0.49) | 0.007 | **Yes** |
| 41 | a_WSL_ | 3 mos-6 mos | -0.20 (-0.40, 0) | 0.05 | **No** |
| 42 | b_WSL_ | 3 mos-6 mos | 1.93 (0.61, 3.26) | 0.004 | **Yes** |
| 43 | Q_WSL_ | 3 mos-6 mos | -0.58 (-0.70, -0.46) | <0.001 | **Yes** |
| 44 | ICDAS II | 3 mos-6 mos | 0.10 (-0.06, 0.26) | 0.22 | **No** |
| 45 | Tx efficiency | 3 mos-6 mos | -0.54 (-3.81, 2.73) | 0.75 | **No** |
| 46 | WSL area | 3 mos-6 mos | -0.14 (-0.65, 0.37) | 0.59 | **No** |
| 47 | WSL area ratio | 3 mos-6 mos | 0.21 (-0.63, 1.05) | 0.62 | **No** |
| 48 | a_WSL_ | 6 mos-12 mos | 0.79 (0.28, 1.30) | 0.003 | **Yes** |
| 49 | b_WSL_ | 6 mos-12 mos | 1.37 (0.56, 2.18) | 0.001 | **Yes** |
| 50 | Grey value | 6 mos-12 mos | 3.24 (2.39, 4.10) | <0.001 | **Yes** |
| 51 | Tx efficiency | 6 mos-12 mos | -0.21 (-3.54, 3.12) | 0.90 | **No** |
| 52 | WSL area | 6 mos-12 mos | -0.03 (-0.54, 0.48) | 0.91 | **No** |
| 53 | L_WSL_ | 12 mos-24 mos | 0.39 (-0.42, 1.20) | 0.35 | **No** |
| 54 | L_WSL/SAE_ | 12 mos-24 mos | 0.34 (-0.37, 1.05) | 0.35 | **No** |
| 55 | a_WSL_ | 12 mos-24 mos | -0.57 (-1.07, -0.07) | 0.02 | **Yes** |
| 56 | a_WSL/SAE_ | 12 mos-24 mos | -0.95 (-1.52, -0.38) | 0.001 | **Yes** |
| 57 | b_WSL_ | 12 mos-24 mos | -1.35 (-2.14, -0.56) | 0.001 | **Yes** |
| 58 | b_WSL/SAE_ | 12 mos-24 mos | -0.53 (-1.25, 0.19) | 0.15 | **No** |
| 59 | DE_WSL/SAE_ | 12 mos-24 mos | 0.37 (-0.51, 1.25) | 0.41 | **No** |
| 60 | DE_WSL/SAE_ | 12 mos-48 mos | -0.01 (-0.35, 0.33) | 0.95 | **No** |
| 61 | Visual score | 12 mos-72 mos | 0 (-0.15, 0.15) | 1.00 | **No** |

DE, L, a, b, CIE Commission Internationale de l’Éclairage L*a*b* color system, color difference, lightness (L), green–red axis (a), blue–yellow axis (b); CI, confidence interval; ICDASII, International Caries Detection and Assessment System II; mo, month; NC, non-calculable; SAE, sound adjacent enamel; ; Tx efficiency, treatment efficiency (for WSL area ratio); VAS, visual analogue scale; WSL, white spot lesion.

**Supplementary Material 10.** Sensitivity analysis: Outcomes assessed by single studies without the study of Gabr 2024.

| **Nr** | **Outcome** | **Time period** | **Change (95% CI)** | **P** | **Statistically significant** |
| --- | --- | --- | --- | --- | --- |
| 1 | L_WSL_ | Post-1 mo | -0.30 (-0.64, 0.04) | 0.08 | **No** |
| 2 | ICDAS II | Post-1 mo | -0.02 (-0.14, 0.10) | 0.74 | **No** |
| 3 | Tx efficiency | Post-1 mo | 13.77 (9.57, 17.97) | <0.001 | **Yes** |
| 4 | WSL area | Post-1 mo | -1.60 (-2.50, -0.70) | 0.001 | **Yes** |
| 5 | WSL area ratio | Post-1 mo | -5.34 (-7.87, -2.82) | <0.001 | **Yes** |
| 6 | DE_WSL_ | Post-3 mos | -0.61 (-1.11, -0.11) | 0.01 | **Yes** |
| 7 | a_WSL_ | Post-3 mos | -0.08 (-0.24, 0.08) | 0.34 | **No** |
| 8 | b_WSL_ | Post-3 mos | -0.06 (-0.92, 0.80) | 0.89 | **No** |
| 9 | Q_WSL_ | Post-3 mos | -0.78 (-0.96, -0.60) | <0.001 | **Yes** |
| 10 | Tx efficiency | Post-3 mos | 18.34 (14.15, 22.53) | <0.001 | **Yes** |
| 11 | WSL area | Post-3 mos | -2.44 (-3.39, -1.49) | <0.001 | **Yes** |
| 12 | WSL area ratio | Post-3 mos | -7.11 (-9.96, -4.43) | <0.001 | **Yes** |
| 13 | L_WSL/SAE_ | Post-6 mos | 0 (-0.58, 0.58) | 1.00 | **No** |
| 14 | a_WSL/SAE_ | Post-6 mos | 0.13 (-0.43, 0.69) | 0.65 | **No** |
| 15 | b_WSL/SAE_ | Post-6 mos | 0.26 (-0.41, 0.93) | 0.45 | **No** |
| 16 | Q_WSL_ | Post-6 mos | -1.36 (-1.55, -1.17) | <0.001 | **Yes** |
| 17 | Grey value | Post-6 mos | -6.87 (-8.20, -5.54) | <0.001 | **Yes** |
| 18 | Tx efficiency | Post-6 mos | 17.80 (13.61, 21.99) | <0.001 | **Yes** |
| 19 | WSL area | Post-6 mos | -2.58 (-3.53, -1.63) | <0.001 | **Yes** |
| 20 | L_WSL_ | Post-12 mos | 1.30 (0.92, 1.68) | <0.001 | **Yes** |
| 21 | L_WSL/SAE_ | Post-12 mos | 0.85 (-0.60, 2.30) | 0.25 | **No** |
| 22 | F_WSL_ | Post-12 mos | -0.07 (-0.19, 0.05) | 0.26 | **No** |
| 23 | F_WSL/SAE_ | Post-12 mos | -0.30 (-0.52, -0.08) | 0.006 | **Yes** |
| 24 | Q_WSL_ | Post-12 mos | 0.06 (-0.06, 0.18) | 0.32 | **No** |
| 25 | Q_WSL/SAE_ | Post-12 mos | -0.50 (-1.01, 0.01) | 0.05 | **No** |
| 26 | Grey value | Post-12 mos | -3.63 (-5.02, -2.24) | <0.001 | **Yes** |
| 27 | Tx efficiency | Post-12 mos | 17.59 (13.40, 21.78) | <0.001 | **Yes** |
| 28 | ICDAS II | Post-12 mos | -0.18 (-0.31, -0.06) | 0.005 | **Yes** |
| 29 | Visual score | Post-12 mos | -0.20 (-0.36, -0.05) | 0.01 | **Yes** |
| 30 | DE_WSL/SAE_ | Post-48 mos | 0.22 (-0.22, 0.66) | 0.33 | **No** |
| 31 | WSL area | Post-72 mos | 0.05 (0, 0.10) | 0.04 | **Yes** |
| 32 | DE_WSL/SAE_ | Post-72 mos | -0.80 (-1.23, -0.37) | <0.001 | **Yes** |
| 33 | F_WSL/SAE_ | Post-72 mos | -0.30 (-0.52, -0.08) | 0.006 | **Yes** |
| 34 | Q_WSL/SAE_ | Post-72 mos | -0.50 (-1.01, 0.01) | 0.05 | **No** |
| 35 | ICDAS II | Post-72 mos | 0.08 (-0.06, 0.22) | 0.25 | **No** |
| 36 | Visual score | Post-72 mos | -0.20 (-0.36, -0.05) | 0.01 | **Yes** |
| 37 | L_WSL_ | 1 mo-3 mos | 0.40 (0.20, 0.60) | <0.001 | **Yes** |
| 38 | ICDAS II | 1 mo-3 mos | 0 (-0.11, 0.11) | 1.00 | **No** |
| 39 | Tx efficiency | 1 mo-3 mos | 4.57 (1.35, 7.79) | 0.005 | **Yes** |
| 40 | WSL area | 1 mo-3 mos | -0.84 (-1.56, -0.13) | 0.02 | **Yes** |
| 41 | WSL area ratio | 1 mo-3 mos | -1.77 (-3.05, -0.49) | 0.007 | **Yes** |
| 42 | DE_WSL_ | 3 mos-6 mos | -0.57 (-1.09, -0.05) | 0.03 | **Yes** |
| 43 | a_WSL_ | 3 mos-6 mos | -0.20 (-0.40, 0) | 0.05 | **No** |
| 44 | b_WSL_ | 3 mos-6 mos | 1.93 (0.61, 3.26) | 0.004 | **Yes** |
| 45 | Q_WSL_ | 3 mos-6 mos | -0.58 (-0.70, -0.46) | <0.001 | **Yes** |
| 46 | ICDAS II | 3 mos-6 mos | 0.10 (-0.06, 0.26) | 0.22 | **No** |
| 47 | Tx efficiency | 3 mos-6 mos | -0.54 (-3.81, 2.73) | 0.75 | **No** |
| 48 | WSL area | 3 mos-6 mos | -0.14 (-0.65, 0.37) | 0.59 | **No** |
| 49 | WSL area ratio | 3 mos-6 mos | 0.21 (-0.63, 1.05) | 0.62 | **No** |
| 50 | a_WSL_ | 6 mos-12 mos | 0.79 (0.28, 1.30) | 0.003 | **Yes** |
| 51 | b_WSL_ | 6 mos-12 mos | 1.37 (0.56, 2.18) | 0.001 | **Yes** |
| 52 | Grey value | 6 mos-12 mos | 3.24 (2.39, 4.10) | <0.001 | **Yes** |
| 53 | Tx efficiency | 6 mos-12 mos | -0.21 (-3.54, 3.12) | 0.90 | **No** |
| 54 | WSL area | 6 mos-12 mos | -0.03 (-0.54, 0.48) | 0.91 | **No** |
| 55 | L_WSL_ | 12 mos-24 mos | 0.39 (-0.42, 1.20) | 0.35 | **No** |
| 56 | L_WSL/SAE_ | 12 mos-24 mos | 0.34 (-0.37, 1.05) | 0.35 | **No** |
| 57 | a_WSL_ | 12 mos-24 mos | -0.57 (-1.07, -0.07) | 0.02 | **Yes** |
| 58 | a_WSL/SAE_ | 12 mos-24 mos | -0.95 (-1.52, -0.38) | 0.001 | **Yes** |
| 59 | b_WSL_ | 12 mos-24 mos | -1.35 (-2.14, -0.56) | 0.001 | **Yes** |
| 60 | b_WSL/SAE_ | 12 mos-24 mos | -0.53 (-1.25, 0.19) | 0.15 | **No** |
| 61 | DE_WSL/SAE_ | 12 mos-24 mos | 0.37 (-0.51, 1.25) | 0.41 | **No** |
| 62 | DE_WSL/SAE_ | 12 mos-48 mos | -0.01 (-0.35, 0.33) | 0.95 | **No** |
| 63 | Visual score | 12 mos-72 mos | 0 (-0.15, 0.15) | 1.00 | **No** |

DE, L, a, b, CIE Commission Internationale de l’Éclairage L*a*b* color system, color difference, lightness (L), green–red axis (a), blue–yellow axis (b); CI, confidence interval; ICDASII, International Caries Detection and Assessment System II; mo, month; NC, non-calculable; SAE, sound adjacent enamel; Tx efficiency, treatment efficiency (for WSL area ratio); VAS, visual analogue scale; WSL, white spot lesion.

**Supplementary Material 11.** Sensitivity analysis: Meta-analyses performed without the study of Gabr 2024.

| **Nr** | **Outcome** | **Time period** | **Studies** | **Change (95% CI)** | **P** | **tau^2^ (95% CI)** | **I^2^ (95% CI)** | **Prediction** |
| --- | --- | --- | --- | --- | --- | --- | --- | --- |
| 1 | DE_WSL/SAE_ | Post-1 mo | 3 | -0.11 (-1.19, 0.98) | 0.71 | 0.17 (0.03, 7.65) | 91% (76%, 96%) | -2.18, 1.96 |
| 2 | LF | Post-1 mo | 2 | -0.12 (-0.42, 0.19) | 0.45 | 0 (-) | 0% (-) | - |
| 3 | Satisfaction_VAS_ | Post-1 mo | 2 | 11.88 (5.93, 17.84) | 0.02 | 0 (-) | 0% (-) | - |
| 4 | L_WSL_ | Post-3 mos | 2 | 0.87 (-0.71, 2.44) | 0.28 | 1.20 (-) | 93% (-) | - |
| 5 | DE_WSL/SAE_ | Post-3 mos | 3 | -0.14 (-0.93, 0.64) | 0.51 | 0.08 (0.01, 4.00) | 84% (51%, 95%) | -1.60, 1.31 |
| 6 | LF | Post-3 mos | 3 | 0.35 (-1.81, 2.50) | 0.75 | 3.08 (0.53, >100) | 98% (96%, 99%) | -25.97, 26.66 |
| 7 | ICDAS II | Post-3 mos | 2 | 0.04 (-0.09, 0.18) | 0.53 | 0.01 (-) | 55% (-) | - |
| 8 | Satisfaction_VAS_ | Post-3 mos | 3 | 9.58 (-4.38, 23.55) | 0.09 | 25.53 (0.94, >100) | 76% (22%, 93%) | -16.79, 35.95 |
| 9 | L_WSL_ | Post-6 mos | 4 | 1.17 (-1.67, 4.01) | 0.28 | 3.07 (0.90, 43.89) | 97% (94%, 98%) | -7.30, 9.63 |
| 10 | a_WSL_ | Post-6 mos | 3 | 0.09 (-0.51, 0.69) | 0.76 | 0.22 (0.03, 8.37) | 89% (71%, 96%) | -7.02, 7.20 |
| 11 | b_WSL_ | Post-6 mos | 3 | 1.43 (-0.21, 3.08) | 0.08 | 1.79 (0.24, 86.16) | 85% (55%, 95%) | -18.64, 21.51 |
| 12 | DE_WSL_ | Post-6 mos | 3 | 1.66 (-4.85, 8.17) | 0.39 | 6.79 (1.79, >100) | 99% (98%, 99%) | -11.30, 14.63 |
| 13 | DE_WSL/SAE_ | Post-6 mos | 6 | 0 (-0.42, 0.41) | 0.98 | 0.13 (0.03, 0.88) | 84% (67%, 92%) | -1.10, 1.01 |
| 14 | LF | Post-6 mos | 4 | 2.20 (-3.30, 7.71) | 0.29 | 11.73 (3.50, >100) | 99% (99%, 99%) | -10.05, 14.45 |
| 15 | ICDAS II | Post-6 mos | 3 | -0.26 (-0.80, 0.28) | 0.35 | 0.22 (0.05, 9.11) | 97% (93%, 98%) | -7.19, 6.68 |
| 16 | Satisfaction_VAS_ | Post-6 mos | 3 | 6.98 (-9.34, 23.30) | 0.20 | 36.94 (4.17, >100) | 84% (50%, 95%) | -24.25, 38.21 |
| 17 | WSL area ratio | Post-6 mos | 2 | -4.04 (-9.29, 1.20) | 0.13 | 13.23 (-) | 92% (-) | - |
| 18 | DE_WSL_ | Post-12 mos | 2 | 2.18 (-5.07, 9.43) | 0.56 | 27.30 (-) | 100% (-) | - |
| 19 | DE_WSL/SAE_ | Post-12 mos | 4 | -0.04 (-0.50, 0.42) | 0.80 | 0.06 (0.01, 1.20) | 79% (42%, 92%) | -0.97, 0.89 |
| 20 | LF | Post-12 mos | 2 | 4.98 (-2.52, 12.48) | 0.19 | 28.15 (-) | 96% (-) | - |
| 21 | Satisfaction_VAS_ | Post-12 mos | 2 | -1.06 (-170.65, 168.53) | 0.94 | 346.78 (-) | 97% (-) | - |
| 22 | WSL area | Post-12 mos | 2 | -1.26 (-3.81, 1.28) | 0.33 | 3.26 (-) | 97% (-) | - |
| 23 | WSL area ratio | Post-12 mos | 2 | -3.99 (-9.16, 1.18) | 0.13 | 12.82 (-) | 92% (-) | - |
| 24 | DE_WSL/SAE_ | 1 mo-3 mos | 3 | 0 (-0.06, 0.05) | 0.87 | 0 (0, 0.58) | 0% (0%, 90%) | -0.35, 0.34 |
| 25 | LF | 1 mo-3 mos | 2 | -1.17 (-4.08, 1.75) | 0.43 | 3.66 (-) | 79% (-) | - |
| 26 | Satisfaction_VAS_ | 1 mo-3 mos | 2 | 0.78 (-1.57, 3.13) | 0.52 | 1.47 (-) | 51% (-) | - |
| 27 | L_WSL_ | 3 mos-6 mos | 2 | 1.19 (0.12, 2.26) | 0.02 | 0.51 (-) | 85% (-) | - |
| 28 | DE_WSL/SAE_ | 3 mos-6 mos | 3 | 0 (-0.06, 0.05) | 0.90 | 0 (0, 0.06) | 0% (0%, 90%) | -0.36, 0.35 |
| 29 | LF | 3 mos-6 mos | 2 | 4.45 (2.95, 5.94) | <0.001 | 0.67 (-) | 36% (-) | - |
| 30 | Satisfaction_VAS_ | 3 mos-6 mos | 3 | -2.13 (-4.33, 0.08) | 0.05 | 0.27 (0, 82.85) | 0% (0%, 90%) | -17.85, 13.60 |
| 31 | L_WSL_ | 6 mos-12 mos | 2 | 0.42 (0.08, 0.76) | 0.01 | 0 (-) | 0% (-) | - |
| 32 | L_WSL/SAE_ | 6 mos-12 mos | 2 | -0.06 (-0.53, 0.41) | 0.81 | 0 (-) | 0% (-) | - |
| 33 | a_WSL/SAE_ | 6 mos-12 mos | 2 | 0.61 (0.03, 1.18) | 0.03 | 0.11 (-) | 64% (-) | - |
| 34 | b_WSL/SAE_ | 6 mos-12 mos | 2 | 0.76 (0, 1.53) | 0.05 | 0.14 (-) | 45% (-) | - |
| 35 | DE_WSL_ | 6 mos-12 mos | 2 | 1.67 (-2.27, 5.61) | 0.40 | 8.00 (-) | 99% (-) | - |
| 36 | DE_WSL/SAE_ | 6 mos-12 mos | 4 | -0.11 (-0.41, 0.19) | 0.34 | 0.01 (0, 1.47) | 59% (0%, 86%) | -0.76, 0.54 |
| 37 | LF | 6 mos-12 mos | 2 | -0.06 (-5.40, 5.27) | 0.98 | 13.71 (-) | 92% (-) | - |
| 38 | Satisfaction_VAS_ | 6 mos-12 mos | 2 | -10.61 (-29.62, 8.40) | 0.27 | 184.16 (-) | 98% (-) | - |
| 39 | WSL area ratio | 6 mos-12 mos | 2 | 0.04 (-0.48, 0.56) | 0.89 | 0 (-) | 0% (-) | - |

DE, L, a, b, CIE Commission Internationale de l’Éclairage L*a*b* color system, color difference, lightness (L), green–red axis (a), blue–yellow axis (b); CI, confidence interval; ICDASII, International Caries Detection and Assessment System II; LF, Laser Fluorescence; mo, month; NC, non-calculable; SAE, sound adjacent enamel; VAS, visual analogue scale; WSL, white spot lesion.

**Supplementary Material 12**. Sensitivity analysis: forest plot for ΔΕ_WSL_ without the study of Gabr 2024.


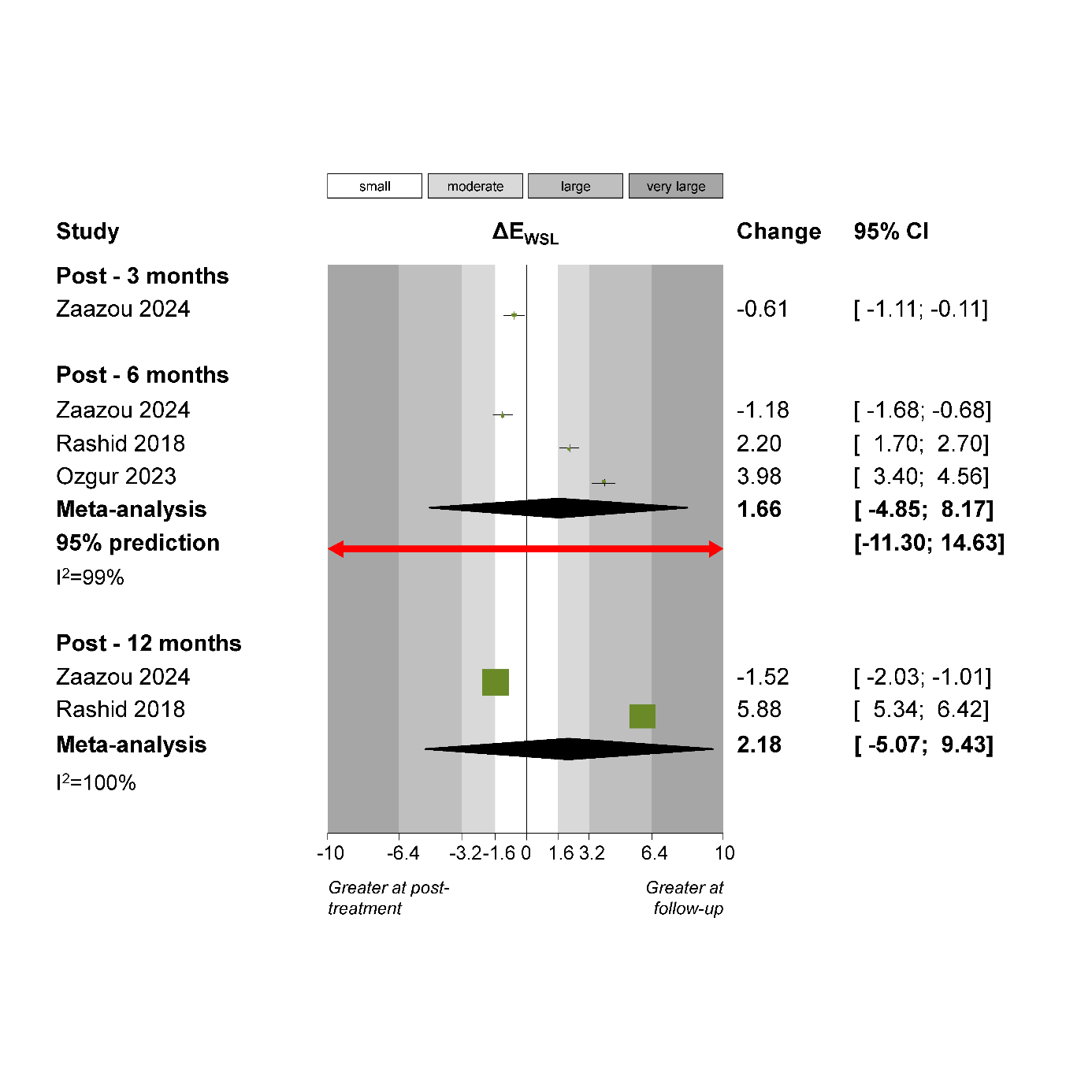


**Supplementary Material 13**. Sensitivity analysis: ΔΕ_WSL/SAE_ post-treatment to 6 months according to study design and sample size.

| **Analysis** | **Subset** | **Studies** | **Change (95% CI)** | **P_between-subsets_** |
| --- | --- | --- | --- | --- |
| By design | Randomized trial | 5 | -0.13 (-0.38, 0.11) | P=0.001 |
|  | Non-randomized study | 1 | 0.66 (0.24, 1.08) |  |
|  |  |  |  |  |
| By sample size | Up to 80 lesions | 4 | -0.07 (-0.33, 0.19) | 0.76 |
|  | More than 80 lesions | 2 | 0.10 (-0.98, 1.19) |  |

CI, confidence interval
